# Novel loci and biomedical consequences of iron homoeostasis variation

**DOI:** 10.1038/s42003-024-07115-3

**Published:** 2024-12-06

**Authors:** Elias Allara, Steven Bell, Rebecca Smith, Spencer J. Keene, Dipender Gill, Liam Gaziano, Deisy Morselli Gysi, Feiyi Wang, Vinicius Tragante, Amy Mason, Savita Karthikeyan, R. Thomas Lumbers, Emmanuela Bonglack, Willem Ouwehand, David J. Roberts, Joseph Dowsett, Sisse Rye Ostrowski, Margit Hørup Larsen, Henrik Ullum, Ole Birger Pedersen, Søren Brunak, Karina Banasik, Christian Erikstrup, DBDS Genomic Consortium, Joseph Dowsett, Sisse Rye Ostrowski, Henrik Ullum, Ole Birger Pedersen, Søren Brunak, Karina Banasik, Jakob Bay, Jens Kjærgaard Boldsen, Thorsten Brodersen, Alfonso Buil Demur, Lea Arregui Nordahl Christoffersen, Maria Didriksen, Khoa Manh Dinh, Christian Erikstrup, Bjarke Feenstra, Frank Geller, Daniel Gudbjartsson, Thomas Folkmann Hansen, Dorte Helenius Mikkelsen, Lotte Hindhede, Henrik Hjalgrim, Jakob Hjorth von Stemann, Bitten Aagaard Jensen, Andrew Joseph Schork, Kathrine Kaspersen, Bertram Dalskov Kjerulff, Mette Kongstad, Susan Mikkelsen, Christina Mikkelsen, Janna Nissen, Mette Nyegaard, Liam James Elgaard Quinn, Þórunn Rafnar, Palle Duun Rohde, Klaus Rostgaard, Michael Schwinn, Erik Sørensen, Kari Stefansson, Hreinn Stefánsson, Lise Wegner Thørner, Unnur Þorsteinsdóttir, Mie Topholm Bruun, Thomas Werge, David Westergaard, Jonathan Mitchell, Christian Fuchsberger, Cristian Pattaro, Peter P. Pramstaller, Domenico Girelli, Mikko Arvas, Jarkko Toivonen, Sophie Molnos, Annette Peters, Ozren Polasek, Igor Rudan, Caroline Hayward, Ciara McDonnell, Nicola Pirastu, James F. Wilson, Katja van den Hurk, Franke Quee, Luigi Ferrucci, Stefania Bandinelli, Toshiko Tanaka, Giorgia Girotto, Maria Pina Concas, Alessandro Pecori, Niek Verweij, Pim van der Harst, Yordi J. van de Vegte, Lambertus A. Kiemeney, Fred C. Sweep, Tessel E. Galesloot, Patrick Sulem, Daniel Gudbjartsson, Egil Ferkingstad, FinnGen Consortium, Aarno Palotie, Mark Daly, Bridget Riley-Gills, Howard Jacob, Dirk Paul, Slavé Petrovski, Chia-Yen Chen, Sally John, George Okafo, Robert Plenge, Joseph Maranville, Mark McCarthy, Rion Pendergrass, Margaret G. Ehm, Kirsi Auro, Simonne Longerich, Anders Mälarstig, Anna Vlahiotis, Katherine Klinger, Clement Chatelain, Matthias Gossel, Karol Estrada, Robert Graham, Dawn Waterworth, Chris O’Donnell, Nicole Renaud, Tomi P. Mäkelä, Jaakko Kaprio, Minna Ruddock, Petri Virolainen, Antti Hakanen, Terhi Kilpi, Markus Perola, Jukka Partanen, Taneli Raivio, Jani Tikkanen, Raisa Serpi, Tarja Laitinen, Veli-Matti Kosma, Jari Laukkanen, Marco Hautalahti, Outi Tuovila, Raimo Pakkanen, Jeffrey Waring, Fedik Rahimov, Ioanna Tachmazidou, Zhihao Ding, Marc Jung, Hanati Tuoken, Shameek Biswas, David Pulford, Neha Raghavan, Adriana Huertas-Vazquez, Jae-Hoon Sul, Xinli Hu, Åsa Hedman, Ma´en Obeidat, Jonathan Chung, Jonas Zierer, Mari Niemi, Samuli Ripatti, Johanna Schleutker, Mikko Arvas, Olli Carpén, Reetta Hinttala, Johannes Kettunen, Arto Mannermaa, Katriina Aalto-Setälä, Mika Kähönen, Johanna Mäkelä, Reetta Kälviäinen, Valtteri Julkunen, Hilkka Soininen, Anne Remes, Mikko Hiltunen, Jukka Peltola, Minna Raivio, Pentti Tienari, Juha Rinne, Roosa Kallionpää, Juulia Partanen, Adam Ziemann, Nizar Smaoui, Anne Lehtonen, Susan Eaton, Heiko Runz, Sanni Lahdenperä, Natalie Bowers, Edmond Teng, Fanli Xu, Laura Addis, John Eicher, Qingqin S. Li, Karen He, Ekaterina Khramtsova, Martti Färkkilä, Jukka Koskela, Sampsa Pikkarainen, Airi Jussila, Katri Kaukinen, Timo Blomster, Mikko Kiviniemi, Markku Voutilainen, Tim Lu, Linda McCarthy, Amy Hart, Meijian Guan, Jason Miller, Kirsi Kalpala, Melissa Miller, Kari Eklund, Antti Palomäki, Pia Isomäki, Laura Pirilä, Oili Kaipiainen-Seppänen, Johanna Huhtakangas, Nina Mars, Apinya Lertratanakul, Coralie Viollet, Marla Hochfeld, Jorge Esparza Gordillo, Fabiana Farias, Nan Bing, Margit Pelkonen, Paula Kauppi, Hannu Kankaanranta, Terttu Harju, Riitta Lahesmaa, Hubert Chen, Joanna Betts, Rajashree Mishra, Majd Mouded, Debby Ngo, Teemu Niiranen, Felix Vaura, Veikko Salomaa, Kaj Metsärinne, Jenni Aittokallio, Jussi Hernesniemi, Daniel Gordin, Juha Sinisalo, Marja-Riitta Taskinen, Tiinamaija Tuomi, Timo Hiltunen, Amanda Elliott, Mary Pat Reeve, Sanni Ruotsalainen, Audrey Chu, Dermot Reilly, Mike Mendelson, Jaakko Parkkinen, Tuomo Meretoja, Heikki Joensuu, Johanna Mattson, Eveliina Salminen, Annika Auranen, Peeter Karihtala, Päivi Auvinen, Klaus Elenius, Esa Pitkänen, Nina Mars, Relja Popovic, Margarete Fabre, Jennifer Schutzman, Diptee Kulkarni, Alessandro Porello, Andrey Loboda, Heli Lehtonen, Stefan McDonough, Sauli Vuoti, Kai Kaarniranta, Joni A. Turunen, Terhi Ollila, Hannu Uusitalo, Juha Karjalainen, Mengzhen Liu, Stephanie Loomis, Erich Strauss, Hao Chen, Kaisa Tasanen, Laura Huilaja, Katariina Hannula-Jouppi, Teea Salmi, Sirkku Peltonen, Leena Koulu, David Choy, Ying Wu, Pirkko Pussinen, Aino Salminen, Tuula Salo, David Rice, Pekka Nieminen, Ulla Palotie, Maria Siponen, Liisa Suominen, Päivi Mäntylä, Ulvi Gursoy, Vuokko Anttonen, Kirsi Sipilä, Hannele Laivuori, Venla Kurra, Laura Kotaniemi-Talonen, Oskari Heikinheimo, Ilkka Kalliala, Lauri Aaltonen, Varpu Jokimaa, Marja Vääräsmäki, Outi Uimari, Laure Morin-Papunen, Maarit Niinimäki, Terhi Piltonen, Katja Kivinen, Elisabeth Widen, Taru Tukiainen, Niko Välimäki, Eija Laakkonen, Jaakko Tyrmi, Heidi Silven, Eeva Sliz, Riikka Arffman, Susanna Savukoski, Triin Laisk, Natalia Pujol, Janet Kumar, Iiris Hovatta, Erkki Isometsä, Hanna Ollila, Jaana Suvisaari, Antti Mäkitie, Argyro Bizaki-Vallaskangas, Sanna Toppila-Salmi, Tytti Willberg, Elmo Saarentaus, Antti Aarnisalo, Elisa Rahikkala, Kristiina Aittomäki, Fredrik Åberg, Mitja Kurki, Aki Havulinna, Juha Mehtonen, Priit Palta, Shabbeer Hassan, Pietro Della Briotta Parolo, Wei Zhou, Mutaamba Maasha, Susanna Lemmelä, Manuel Rivas, Aoxing Liu, Arto Lehisto, Andrea Ganna, Vincent Llorens, Henrike Heyne, Joel Rämö, Rodos Rodosthenous, Satu Strausz, Tuula Palotie, Kimmo Palin, Javier Garcia-Tabuenca, Harri Siirtola, Tuomo Kiiskinen, Jiwoo Lee, Kristin Tsuo, Kati Kristiansson, Kati Hyvärinen, Jarmo Ritari, Katri Pylkäs, Minna Karjalainen, Tuomo Mantere, Eeva Kangasniemi, Sami Heikkinen, Nina Pitkänen, Samuel Lessard, Lila Kallio, Tiina Wahlfors, Eero Punkka, Sanna Siltanen, Teijo Kuopio, Anu Jalanko, Huei-Yi Shen, Risto Kajanne, Mervi Aavikko, Helen Cooper, Denise Öller, Rasko Leinonen, Henna Palin, Malla-Maria Linna, Masahiro Kanai, Zhili Zheng, L. Elisa Lahtela, Mari Kaunisto, Elina Kilpeläinen, Timo P. Sipilä, Oluwaseun Alexander Dada, Awaisa Ghazal, Anastasia Kytölä, Rigbe Weldatsadik, Kati Donner, Anu Loukola, Päivi Laiho, Tuuli Sistonen, Essi Kaiharju, Markku Laukkanen, Elina Järvensivu, Sini Lähteenmäki, Lotta Männikkö, Regis Wong, Auli Toivola, Minna Brunfeldt, Hannele Mattsson, Sami Koskelainen, Tero Hiekkalinna, Teemu Paajanen, Shuang Luo, Shanmukha Sampath Padmanabhuni, Marianna Niemi, Javier Gracia-Tabuenca, Mika Helminen, Tiina Luukkaala, Iida Vähätalo, Jyrki Tammerluoto, Sarah Smith, Tom Southerington, Petri Lehto, Luc Djousse, Kelly Cho, Michael Inouye, Stephen Burgess, Beben Benyamin, Konrad Oexle, Dorine Swinkels, Kari Stefansson, Magnus Magnusson, Andrea Ganna, Michael Gaziano, Kerry Ivey, John Danesh, Alexandre Pereira, Angela M. Wood, Adam S. Butterworth, Emanuele Di Angelantonio

**Affiliations:** 1 BHF Cardiovascular Epidemiology Unit, Department of Public Health and Primary Care, University of Cambridge, Cambridge, UK; 2 NIHR Blood and Transplant Research Unit in Donor Health and Behaviour, Cambridge, UK; 3 Victor Phillip Dahdaleh Heart and Lung Research Institute, University of Cambridge, Cambridge, UK; 4 Precision Breast Cancer Institute, Department of Oncology, University of Cambridge, Cambridge, UK; 5 Cancer Research UK Cambridge Centre, Li Ka Shing Centre, University of Cambridge, Cambridge, UK; 6 Department of Epidemiology and Biostatistics, School of Public Health, Imperial College London, London, UK; 7 Massachusetts Veterans Epidemiology Research and Information Center (MAVERIC), VA Boston Healthcare System, Boston, MA USA; 8 Department of Medicine, Harvard Medical School, Boston, MA USA; 9 Department of Statistics, Federal University of Parana, Curitiba, Brazil; 10 Division of Aging, Department of Medicine, Brigham and Women’s Hospital, Harvard Medical School, Boston, MA USA; 11 Genetic Epidemiology Lab, Institute for Molecular Medicine Finland, Helsinki, Finland; 12 deCODE genetics/Amgen Inc., Reykjavik, Iceland; 13 UCL Institute of Health Informatics, London, UK; 14 BHF Centre of Research Excellence, School of Clinical Medicine, Addenbrooke’s Hospital, Cambridge, UK; 15 Department of Haematology, University of Cambridge, Cambridge, UK; 16 NHS Blood and Transplant, Cambridge Biomedical Campus, Cambridge, UK; 17 Department of Haematology, Cambridge University Hospitals NHS Trust, Cambridge, UK; 18 Department of Haematology, University College London Hospitals NHS Trust, London, UK; 19 Radcliffe Department of Medicine, University of Oxford, John Radcliffe Hospital, Oxford, UK; 20 Department of Haematology, Churchill Hospital, Headington, Oxford, UK; 21 Department of Clinical Immunology, Copenhagen University Hospital, Rigshospitalet, Copenhagen, Denmark; 22 Department of Clinical Medicine, Faculty of Health and Medical Sciences, University of Copenhagen, Copenhagen, Denmark; 23 Statens Serum Institut, Copenhagen, Denmark; 24 Department of Immunology, Zealand University Hospital, Køge, Denmark; 25 Novo Nordisk Foundation Center for Protein Research, Faculty of Health and Medical Sciences, University of Copenhagen, Copenhagen, Denmark; 26 Department of Immunology, Aarhus University Hospital, Aarhus, Denmark; 27 Eurac Research, Institute for Biomedicine, Bolzano, Italy; 28 Department of Neurology, General Central Hospital, Bolzano, Italy; 29 Department of Medicine, Section of Internal Medicine, EuroBloodNet Referral Center, University Hospital of Verona, Verona, Italy; 30 Finnish Red Cross Blood Service, Helsinki, Finland; 31 msg life central europe gmbh, München, Germany; 32 Institute of Epidemiology, Helmholtz Munich, Neuherberg, Germany; 33 German Center for Diabetes Research (DZD), Neuherberg, Germany; 34 Institute for Medical Information Processing, Biometry and Epidemiology, Medical Faculty, Ludwig-Maximilians-Universität München, München, Germany; 35 Faculty of Medicine, University of Split, Split, Croatia; 36 Centre for Global Health Research, Usher Institute, University of Edinburgh, Edinburgh, Scotland Edinburgh, UK; 37 Medical Research Council Human Genetics Unit, Institute of Genetics and Cancer, University of Edinburgh, Edinburgh, UK; 38 Centre for Cardiovascular Sciences, Queens Medical Research Institute, University of Edinburgh, Edinburgh, Scotland UK; 39 Genomics Research Centre, Human Technopole, Milan, Italy; 40 Donor Studies, Department of Donor Medicine Research, Sanquin Research, Amsterdam, The Netherlands; 41 Department of Public and Occupational Health, Amsterdam Public Health Research Institute, Amsterdam UMC, Amsterdam, The Netherlands; 42 Longitudinal studies section, National Institute on Aging, Baltimore, MD USA; 43 Geriatric Unit, Azienda Sanitaria Firenze (ASF), Florence, Italy; 44 Institute for Maternal and Child Health - IRCCS, Burlo Garofolo, Trieste, Italy; 45 Department of Medicine, Surgery and Health Sciences, University of Trieste, Trieste, Italy; 46 Department of Cardiology, University of Groningen, University Medical Center Groningen, Groningen, The Netherlands; 47 Regeneron Genetics Center, Tarrytown, NY USA; 48 Department of Cardiology, University Medical Center Utrecht, Utrecht, The Netherlands; 49 IQ Health, Radboud University Medical Center, Nijmegen, The Netherlands; 50 Department of Urology, Radboud University Medical Center, Nijmegen, The Netherlands; 51 Department of Laboratory Medicine, Radboud University Medical Center, Nijmegen, The Netherlands; 52 School of Engineering and Natural Sciences, University of Iceland, Reykjavik, Iceland; 53 Department of Nutrition, Harvard T. H. Chan School of Public Health, Boston, MA USA; 54 Cambridge Baker Systems Genomics Initiative, Baker Heart and Diabetes Institute, Melbourne, Australia; 55 Health Data Research UK Cambridge, Wellcome Genome Campus and University of Cambridge, Cambridge, UK; 56 Cambridge Baker Systems Genomics Initiative, Department of Public Health and Primary Care, University of Cambridge, Cambridge, UK; 57 Medical Research Council Biostatistics Unit, Cambridge, UK; 58 Australian Centre for Precision Health & Allied Health and Human Performance, University of South Australia, Adelaide, Australia; 59 South Australian Health and Medical Research Institute, Adelaide, Australia; 60 Neurogenetic Systems Analysis Group, Institute of Neurogenomics, Helmholtz Munich, Neuherberg, Germany; 61 Institute of Human Genetics, School of Medicine, Technical University of Munich, München, Germany; 62 Sanquin Blood Bank, Amsterdam, The Netherlands; 63 Faculty of Medicine, School of Health Sciences, University of Iceland, Reykjavik, Iceland; 64 Department of Human Genetics, Wellcome Sanger Institute, Hinxton, UK; 65 Cambridge Centre of Artificial Intelligence in Medicine, Cambridge, UK; 66 Health Data Science Centre, Human Technopole, Milan, Italy; 67 Institute of Biological Psychiatry, Mental Health Centre, Sct. Hans, Copenhagen University Hospital, Roskilde, Denmark; 68 Department of Clinical Medicine, Health, Aarhus University, Aarhus, Denmark; 69 Department of Epidemiology Research, Statens Serum Institut, Copenhagen, Denmark; 70 deCODE Genetics, Reykjavik, Iceland; 71 Danish Headache Center, Department of Neurology, Copenhagen University Hospital, Rigshospitalet-Glostrup, Copenhagen, Denmark; 72 Danish Cancer Society Research Center, Copenhagen, Denmark; 73 Department of Clinical Immunology, Aalborg University Hospital, Aalborg, Denmark; 74 Department of Health Science and Technology, Faculty of Medicine, Aalborg Univeristy, Aalborg, Denmark; 75 Department of Clinical Immunology, Odense University Hospital, Odense, Denmark; 76 Institute for Molecular Medicine Finland (FIMM), HiLIFE, University of Helsinki, Helsinki, Finland; 77 Broad Institute of MIT and Harvard, Boston, MA USA; 78 Massachusetts General Hospital, New Boston, NY USA; 79 Abbvie, Chicago, IL USA; 80 Astra Zeneca, Cambridge, United Kingdom; 81 Biogen, Cambridge, MA USA; 82 Boehringer Ingelheim, Ingelheim am Rhein, Germany; 83 Bristol Myers Squibb, New York, NY USA; 84 Genentech, San Francisco, CA USA; 85 GlaxoSmithKline, Collegeville, PA USA; 86 GlaxoSmithKline, Espoo, Finland; 87 Merck, Kenilworth, NJ USA; 88 Pfizer, New York, NY USA; 89 Translational Sciences, Sanofi R&D, Framingham, MA USA; 90 Maze Therapeutics, San Francisco, CA USA; 91 Janssen Research & Development, LLC, Spring House, PA USA; 92 Novartis Institutes for BioMedical Research, Cambridge, MA USA; 93 HiLIFE, University of Helsinki, Helsinki, Finland; 94 Arctic biobank/University of Oulu, Oulu, Finland; 95 Auria Biobank/University of Turku/Hospital District of Southwest Finland, Turku, Finland; 96 THL Biobank/Finnish Institute for Health and Welfare (THL), Helsinki, Finland; 97 Finnish Red Cross Blood Service/Finnish Hematology Registry and Clinical Biobank, Helsinki, Finland; 98 Helsinki Biobank/Helsinki University and Hospital District of Helsinki and Uusimaa, Helsinki, Finland; 99 Northern Finland Biobank Borealis/University of Oulu/Northern Ostrobothnia Hospital District, Oulu, Finland; 100 Finnish Clinical Biobank Tampere/University of Tampere/Pirkanmaa Hospital District, Tampere, Finland; 101 Pirkanmaa Hospital District, Tampere, Finland; 102 Biobank of Eastern Finland/University of Eastern Finland/Northern Savo Hospital District, Kuopio, Finland; 103 Central Finland Biobank/University of Jyväskylä/Central Finland Health Care District, Jyväskylä, Finland; 104 Central Finland Health Care District, Jyväskylä, Finland; 105 FINBB - Finnish biobank cooperative, Kuopio, Finland; 106 Business Finland, Helsinki, Finland; 107 GlaxoSmithKline, Stevenage, United Kingdom; 108 University of Gothenburg, Gothenburg, Sweden; 109 Seinäjoki Central Hospital, Seinäjoki, Finland; 110 Finnish Institute for Health and Welfare (THL), Helsinki, Finland; 111 Northern Ostrobothnia Hospital District, Oulu, Finland; 112 Faculty of Medicine and Health Technology, Tampere University, Tampere, Finland; 113 Department of Oncology, Helsinki University Hospital Comprehensive Cancer Center and University of Helsinki, Helsinki, Finland; 114 Research Unit of Oral Health Sciences Faculty of Medicine, University of Oulu, Oulu, Finland; 115 Hospital District of Southwest Finland, Turku, Finland; 116 Institute for Molecular Medicine Finland, HiLIFE, University of Helsinki, Helsinki, Finland; 117 Hospital District of Helsinki and Uusimaa, Helsinki, Finland; 118 Department of Molecular Genetics, University of Lodz, Lodz, Poland; 119 Helsinki University Hospital and University of Helsinki, Helsinki, Finland; 120 Northern Savo Hospital District, Kuopio, Finland; 121 University of Eastern Finland, Kuopio, Finland; 122 Novartis, Boston, MA USA; 123 GlaxoSmithKline, Brentford, United Kingdom; 124 Department of Medical Genetics, Helsinki University Central Hospital, Helsinki, Finland; 125 Tampere University, Tampere, Finland; 126 Janssen Research & Development, LLC, Titusville, NJ 08560 USA; 127 Broad Institute, Cambridge, MA USA; 128 Janssen Research & Development, LLC, Boston, MA USA; 129 Novartis, Basel, Switzerland; 130 Department of Breast Surgery, Helsinki University Hospital Comprehensive Cancer Center and University of Helsinki, Helsinki, Finland; 131 Janssen-Cilag Oy, Espoo, Finland; 132 Department of Otorhinolaryngology - Head and Neck Surgery, University of Helsinki and Helsinki University Hospital, Helsinki, Finland; 133 Estonian biobank, Tartu, Estonia; 134 Eye Genetics Group, Folkhälsan Research Center, Helsinki, Finland; 135 Medical Research Center, Oulu, Oulu University Hospital and University of Oulu, Oulu, Finland; 136 University of Eastern Finland and Kuopio University Hospital, Department of Otorhinolaryngology, Kuopio, Finland; 137 University of Helsinki, Helsinki, Finland; 138 University of Jyväskylä, Jyväskylä, Finland; 139 University of Tampere, Tampere, Finland; 140 University of Oulu, Oulu, Finland; 141 Department of Allergy, Helsinki University Hospital and University of Helsinki, Helsinki, Finland; 142 Transplantation and Liver Surgery Clinic, Helsinki University Hospital, Helsinki University, Helsinki, Finland; 143 University of Stanford, Stanford, CA USA; 144 University of Helsinki and Hospital District of Helsinki and Uusimaa, Helsinki, Finland; 145 European Molecular Biology Laboratory, European Bioinformatics Institute, Cambridge, UK

**Keywords:** Epidemiology, Genomics

## Abstract

Iron homoeostasis is tightly regulated, with hepcidin and soluble transferrin receptor (sTfR) playing significant roles. However, the genetic determinants of these traits and the biomedical consequences of iron homoeostasis variation are unclear. In a meta-analysis of 12 cohorts involving 91,675 participants, we found 43 genomic loci associated with either hepcidin or sTfR concentration, of which 15 previously unreported. Mapping to putative genes indicated involvement in iron-trait expression, erythropoiesis, immune response and cellular trafficking. Mendelian randomisation of 292 disease outcomes in 1,492,717 participants revealed associations of iron-related loci and iron status with selected health outcomes across multiple domains. These associations were largely driven by *HFE*, which was associated with the largest iron variation. Our findings enhance understanding of iron homoeostasis and its biomedical consequences, suggesting that lifelong exposure to higher iron levels is likely associated with lower risk of anaemia-related disorders and higher risk of genitourinary, musculoskeletal, infectious and neoplastic diseases.

## Abstract (web summary)

The largest meta-analysis of GWASs of hepcidin and sTfR identified 43 genomic loci, of which 15 new. Mendelian randomisation revealed associations of systemic iron status and iron-related loci with selected health outcomes across multiple domains.

## Introduction

Iron is essential for various biological functions, including respiration, energy production, DNA synthesis, and cell proliferation^1,2^. Iron homoeostasis in healthy individuals is tightly regulated, with hepcidin and soluble transferrin receptor (sTfR) playing significant roles. Hepcidin, a liver-produced peptide hormone, regulates systemic iron levels by suppressing dietary iron absorption and recycling in response to elevated iron levels^1,3^. sTfR, the circulating extracellular part of transferrin receptor 1, serves as a biomarker indicating iron demand relative to supply, although its biological function is largely unknown^1,4^. Despite their relevance to iron homoeostasis and potential clinical utility for assessing iron status in adults^1^, the genetic determinants of hepcidin and sTfR concentrations remain poorly understood^5,6^, with previous large-scale genome-wide association studies (GWASs) primarily focusing on conventional clinical biomarkers such as serum iron, ferritin, transferrin saturation (TSAT), and either transferrin or total iron-binding capacity (TIBC)^7–9^.

Disruption in iron homoeostasis can cause iron deficiency and iron overload. Iron deficiency affects over two billion people worldwide^1^, which underscores the need to understand its long-term consequences on population health. Although iron overload is less prevalent, its extreme form—hemochromatosis—can lead to severe clinical manifestations^10^. Previous research on iron-regulating pathways has primarily focused on exploring the long-term biomedical consequences of perturbations in *HFE*^11^, a genetic locus involved in the aetiology of hemochromatosis. The long-term clinical associations of systemic iron status have been assessed in multiple observational studies^12–19^, Mendelian randomisation (MR) investigations^20–25^, and randomised trials^26–30^, with uncertainty mainly arising from residual bias in observational studies, limited statistical power and pleiotropy in MR investigations, and the breadth of health outcomes analysed in randomised trials.

To enhance the understanding of the genetic regulation of hepcidin and sTfR, we combined data from 12 original GWASs. We identified and described 43 genomic loci, including 2 new loci associated with hepcidin and 13 new loci associated with sTfR that had not been reported in any previous GWAS of iron-related biomarkers. To address the uncertainties related to the long-term consequences of individual iron-regulating pathways and systemic iron status on health outcomes, we performed locus-based and polygenic phenome-wide MR analyses on 292 clinical outcomes in up to 1,492,717 participants from deCODE, FinnGen, the Million Veteran Programme (MVP) and UK Biobank (UKBB), and 47 biomedical traits in up to 860,060 participants from MVP and UKBB.

## Results

### Genetic predictors of hepcidin and sTfR

**Fig. 1:**
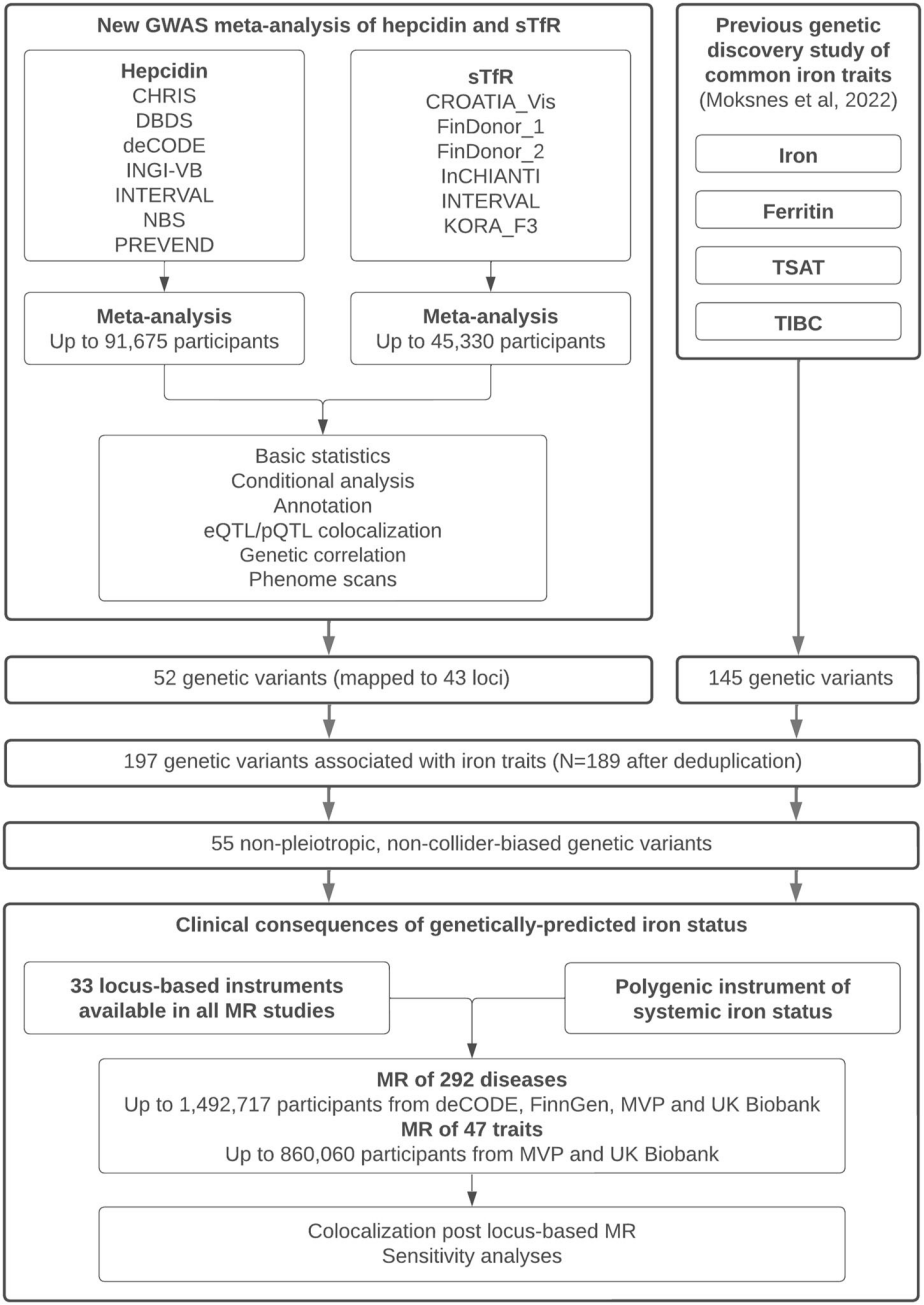
Study overview. GWAS, genome-wide association study. sTfR soluble transferrin receptor, TSAT transferrin saturation, TIBC total iron-binding capacity, eQTL expression quantitative trait loci, pQTL protein quantitative trait loci, MR Mendelian randomisation, MVP Million Veteran Programme. The genetic variants from Moksnes 2022 were obtained from the paper’s Supplementary Data 1.

**Fig. 2:**
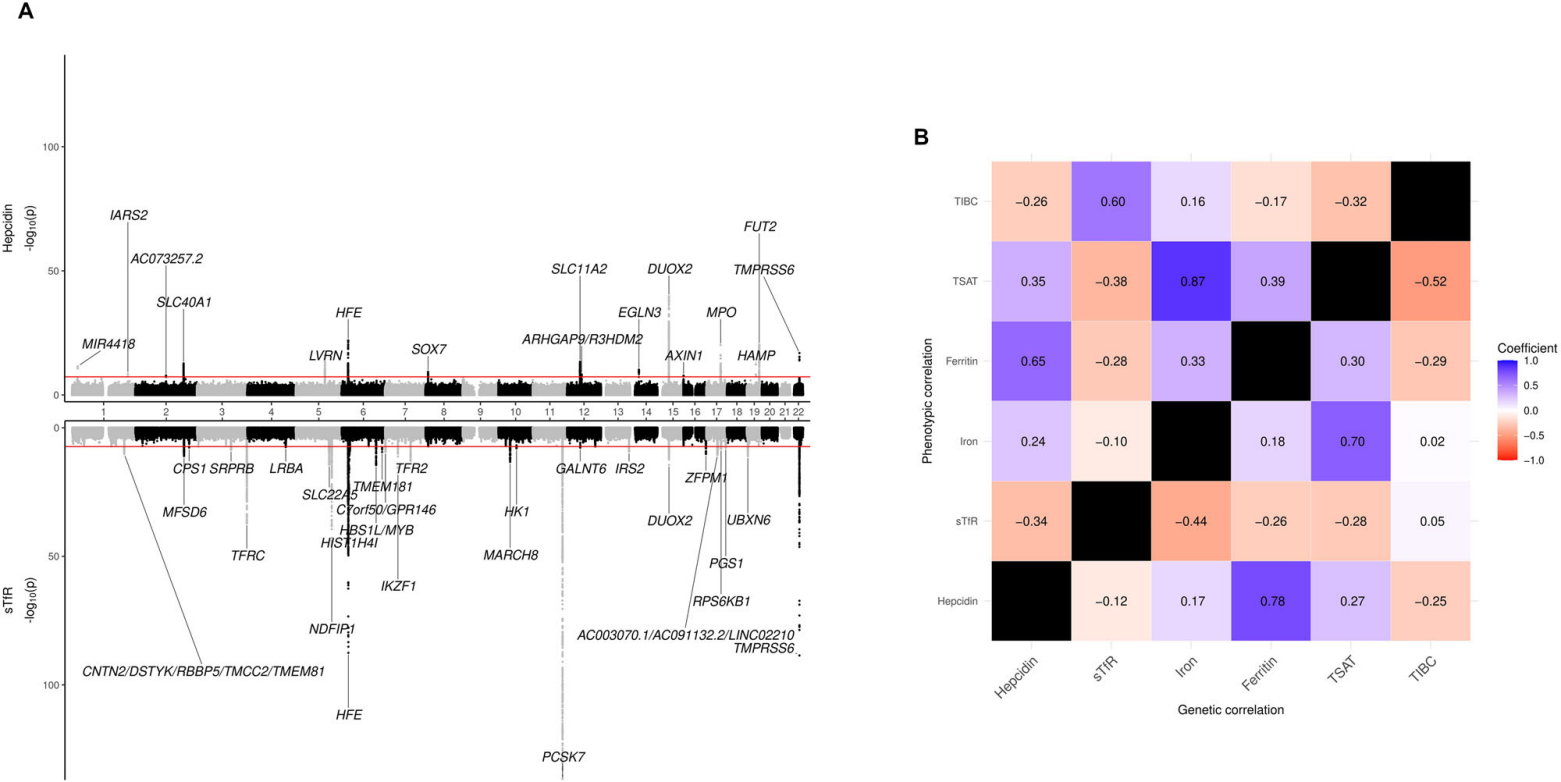
Results of GWAS meta-analysis of hepcidin and sTfR and correlations with common iron traits. **A** Miami plot for hepcidin (upper plot, *N* = 91,675 participants) and sTfR (lower plot, *N* = 45,330 participants). For each locus (*N* = 16 loci for hepcidin, *N* = 27 for sTfR), we show the candidate gene name for the sentinel variant with the lowest *p* value. **B** Genetic and phenotypic correlations between the iron traits analysed in this study (hepcidin, sTfR) and those investigated in previous studies (ferritin, iron, TIBC, TSAT). Phenotypic correlations were estimated in the INTERVAL study (up to 40,197 participants). Genetic correlations were estimated using associations from the present study (hepcidin, sTfR; up to 91,675 participants) and Moksnes et al. 2022 (ferritin, iron, TIBC, TSAT; up to 257,953 participants).

We included 12 cohorts with imputed genotype array data comprising up to 91,675 participants and 16,261,412 variants with assessments of hepcidin concentration and up to 45,330 participants and 13,606,859 variants with measurements of sTfR concentration (Fig. 1; Supplementary Data 1). Across participating cohorts, the mean age ranged between 40 and 67 years, and the percentage of female participants ranged between 47% and 61% (Supplementary Data 1). All studies included admixed European-ancestry participants. Using LD Score regression and the 1000 G EUR reference panel, common SNP-based heritability estimates were 4.1% for hepcidin and 16.5% for sTfR (by comparison, heritability ranged between 15–48% in recent GWASs of conventional iron biomarkers^8,9^), and genetic associations were typically weaker for hepcidin compared to sTfR (Fig. 2A). Please note that we provide definitions of common genetic terminology in Table 1. Sensitivity analyses only adjusted for age and sex show similar results (Supplementary Information, page 9). Sensitivity analyses adjusted for C-reactive protein, in addition to the other covariates included in the main analysis, also show results similar to the main model (Supplementary Information, pages 9–10). Genetic and phenotypic correlations between hepcidin, sTfR and other iron traits (serum iron, ferritin, TSAT, TIBC) were broadly concordant (Fig. 2B; Supplementary Data 2).

**Table 1:** Glossary of genetic terms

Term	Acronym	Description
Colocalization	-	Statistical method used to determine whether two traits share a causal variant within a specific locus.
Expression quantitative trait locus	eQTL	Genetic loci that explain variation in mRNA expression levels.
Genome-wide association study	GWAS	Research approach for identifying genomic variants that are statistically linked to a particular trait.
Linkage disequilibrium	LD	Non-random association of alleles at different loci. It can be estimated statistically using the correlation coefficient between pairs of loci (*r*^2^).
Locus	-	Specific position on a chromosome where a particular gene or genetic marker is located.
Mendelian randomization	MR	Statistical method for assessing causal relationships that tests whether genetic variants associated with an exposure (e.g., iron status) are also associated with an outcome (e.g., diseases or intermediate traits).
Minor allele frequency	MAF	Frequency at which the second most common allele occurs in a given population. Common alleles typically have a MAF ≥ 0.1.
Protein quantitative trait locus	pQTL	Genetic loci that explain variation in protein levels.
Single nucleotide polymorphism	SNP	Genomic variant occurring at a single nucleotide position in the DNA sequence.
Variant	-	A change in the most common DNA nucleotide sequence.

We found 52 genome-wide significant (*P* < 5 × 10^−8^), conditionally independent and uncorrelated (*r*^2^ < 0.01) signals mapped to 43 loci (Table 2; Supplementary Data 3; Supplementary Data 4). Of these, we found 20 associations with hepcidin (mapped to 16 loci) and 32 associations with sTfR (27 loci). All these 16 loci are associated with hepcidin for the first time, and two of them have not been reported in previous GWASs of iron biomarkers^7–9^. Twenty-four loci are associated with sTfR for the first time and 13 are previously unreported in GWASs of iron biomarkers. In Supplementary Data 3, we annotate the studies where the loci were previously reported. Three loci (*DUOX2*, *HFE*, *TMPRSS6*) contained signals for both hepcidin and sTfR, suggesting some shared genetic aetiology for these two biomarkers. We found 13 out of 52 sentinel variants with the strongest evidence for association (*P* < 1 × 10^−15^) in known loci such as *DUOX2*, *HFE* and *PCSK7*, and two variants in new loci (rs116816795, *P* = 3.13 × 10^−46^, nearest gene: *NDFIP1;* rs885122, *P* = 2.28 × 10^−18^, *LVRN*) (Table 2; Supplementary Data 3; Supplementary Data 4), suggesting potential involvement of immune response (*LVRN*) in affecting hepcidin and a potential connection between iron import regulation (*NDFIP1*) and sTfR.

**Table 2:** Conditionally independent and uncorrelated sentinel variants associated with hepcidin (*N* = 20) and sTfR (*N* = 32)

Trait	Chr	Position	RsID	EA	NEA	EAF	Beta	SE	*P* value	Het. *P* value	*N*	Nearest gene	Candidate gene	Locus novelty (hepcidin and sTfR)	Variant novelty (all iron traits)	Locus novelty (all iron traits)
Hepcidin	1	22,584,002	rs75965181	A	T	0.023	−0.118	0.017	2.76E-12	2.13E-01	91675	*MIR4418*	*MIR4418*	Novel	Known	Known
	1	220,291,414	rs201255457	A	ATC	0.989	−0.156	0.026	3.15E-09	3.77E-01	75972	*IARS2*	*IARS2*	Novel	Known	Known
	2	121,310,269	rs6706968	A	C	0.424	0.030	0.005	1.43E-08	6.11E-01	82690	*AC073257.1*	*AC073257.2*	Novel	Known	Known
	2	190,390,963	rs149682241	C	G	0.026	0.101	0.015	4.33E-11	8.47E-01	91675	*SLC40A1*	*SLC40A1*	Novel	Novel	Known
	2	190,521,054	rs7568449	T	C	0.762	0.043	0.006	1.88E-13	2.24E-01	89099	*ASNSD1*	*SLC40A1*	Novel	Novel	Known
	5	115,331,335	rs885122	A	G	0.598	−0.043	0.005	2.28E-18	8.07E-09	91675	*LVRN*	*LVRN*	Novel	Novel	Novel
	6	26,098,474	rs79220007	T	C	0.930	0.094	0.010	1.12E-22	1.25E-03	91675	*HFE*	*HFE*	Novel	Known	Known
	8	10,577,987	rs10090558	A	G	0.918	−0.060	0.010	4.64E-10	2.32E-03	78548	*SOX7*	*SOX7*	Novel	Known	Known
	12	51,439,858	rs9739943	T	C	0.059	0.079	0.010	3.78E-14	2.04E-01	91675	*LETMD1*	*SLC11A2*	Novel	Known	Known
	12	57,735,045	rs11609805	A	G	0.270	−0.032	0.006	5.24E-09	4.48E-01	91675	*R3HDM2*	*ARHGAP9/R3HDM2*	Novel	Novel	Novel
	14	34,410,892	rs996347	T	C	0.645	−0.033	0.005	6.06E-11	2.04E-02	91675	*EGLN3*	*EGLN3*	Novel	Known	Known
	15	45,274,530	rs549876436	T	C	0.907	0.082	0.011	7.30E-15	8.99E-02	83099	*TERB2*	*DUOX2*	Novel	Novel	Known
	15	45,319,959	rs2600895	A	G	0.913	0.082	0.012	2.73E-11	7.53E-02	88174	*SORD*	*DUOX2*	Novel	Novel	Known
	15	45,320,493	rs113000093	C	G	0.089	−0.078	0.013	7.01E-10	4.66E-02	85598	*SORD*	*DUOX2*	Novel	Novel	Known
	15	45,387,550	rs199138	A	G	0.076	−0.122	0.009	1.55E-40	3.41E-03	91675	*DUOX2*	*DUOX2*	Novel	Known	Known
	16	353,122	rs71378510	A	G	0.907	0.050	0.009	1.62E-08	5.31E-01	91675	*AXIN1*	*AXIN1*	Novel	Novel	Known
	17	56,436,109	rs34523089	T	C	0.162	0.063	0.007	8.15E-21	8.61E-01	89817	*RNF43*	*MPO*	Novel	Known	Known
	19	35,775,902	rs104894696	A	G	0.003	−0.387	0.054	4.90E-13	2.09E-04	76690	*HAMP*	*HAMP*	Novel	Novel	Known
	19	49,207,255	rs485073	A	G	0.453	0.046	0.005	4.64E-21	5.91E-01	91675	*FUT2*	*FUT2*	Novel	Known	Known
	22	37,462,936	rs855791	A	G	0.429	0.042	0.005	1.34E-17	8.89E-02	91675	*TMPRSS6*	*TMPRSS6*	Novel	Known	Known
sTfR	1	205,041,952	rs6696846	T	C	0.484	−0.043	0.007	6.22E-11	7.35E-01	45330	*CNTN2*	*CNTN2/DSTYK/RBBP5/TMCC2/TMEM81*	Novel	Novel	Novel
	2	191,357,694	rs35095338	A	T	0.556	−0.046	0.007	9.67E-12	8.77E-01	44341	*TMEM194B*	*MFSD6*	Novel	Novel	Novel
	2	211,543,055	rs715	T	C	0.688	−0.041	0.007	2.28E-08	4.12E-01	45330	*CPS1*	*CPS1*	Novel	Known	Known
	3	133,539,500	rs11371594	G	GA	0.845	0.058	0.010	2.54E-09	3.95E-01	40837	*SRPRB*	*SRPRB*	Novel	Novel	Known
	3	195,795,618	rs112856048	A	G	0.243	−0.100	0.008	5.93E-38	5.28E-01	45330	*TFRC*	*TFRC*	Novel	Known	Known
	3	195,921,311	rs9325434	A	G	0.141	−0.062	0.010	2.27E-10	8.47E-01	45330	*ZDHHC19*	*TFRC*	Novel	Novel	Known
	4	151,199,080	rs2290846	A	G	0.277	−0.041	0.007	3.11E-08	9.61E-01	45330	*LRBA*	*LRBA*	Novel	Novel	Novel
	5	131,784,393	rs12521868	T	G	0.427	0.052	0.007	4.41E-15	7.83E-01	45330	*C5orf56*	*SLC22A5*	Novel	Novel	Novel
	5	141,482,333	rs116816795	T	C	0.168	0.127	0.009	3.13E-46	7.55E-01	45330	*NDFIP1*	*NDFIP1*	Novel	Novel	Novel
	5	141,602,204	rs2906082	T	C	0.783	0.057	0.008	5.34E-12	5.65E-01	44584	*NDFIP1*	*NDFIP1*	Novel	Novel	Novel
	6	25,957,426	rs72832593	T	C	0.897	0.130	0.011	8.09E-33	5.91E-01	45330	*TRIM38*	*HIST1H4I*	Novel	Known	Known
	6	26,093,141	rs1800562	A	G	0.075	−0.253	0.013	2.88E-88	2.95E-02	45330	*HFE*	*HFE*	Known	Known	Known
	6	135,427,159	rs9389269	T	C	0.729	0.059	0.007	3.10E-15	6.84E-01	45330	*HBS1L*	*HBS1L/MYB*	Novel	Known	Known
	6	159,020,121	rs143437464	A	G	0.009	−0.234	0.037	3.81E-10	5.08E-01	43595	*TMEM181*	*TMEM181*	Novel	Novel	Novel
	6	159,026,327	rs200307986	A	G	0.003	0.358	0.065	2.69E-08	1.29E-01	42671	*TMEM181*	*TMEM181*	Novel	Novel	Novel
	7	1,080,897	rs186044114	T	C	0.852	−0.061	0.010	4.19E-10	4.27E-01	41188	*C7orf50*	*C7orf50/GPR146*	Novel	Novel	Novel
	7	50,427,982	rs6592965	A	G	0.458	−0.058	0.007	2.43E-18	9.60E-02	45330	*IKZF1*	*IKZF1*	Novel	Known	Known
	7	100,235,970	rs7385804	A	C	0.627	−0.051	0.007	1.49E-13	8.85E-01	45330	*TFR2*	*TFR2*	Novel	Known	Known
	10	45,953,767	rs7908745	A	G	0.687	−0.054	0.007	5.61E-14	1.38E-01	45330	*MARCH8*	*MARCH8*	Novel	Novel	Novel
	10	71,093,392	rs16926246	T	C	0.133	0.056	0.010	1.05E-08	9.71E-01	45330	*HK1*	*HK1*	Novel	Known	Known
	11	117,021,097	rs187669805	C	G	0.994	0.407	0.046	1.94E-18	3.86E-01	43487	*PCSK7*	*PCSK7*	Known	Novel	Known
	11	117,081,500	rs11216316	A	C	0.892	−0.273	0.011	1.77E-137	5.68E-02	45330	*PCSK7*	*PCSK7*	Known	Novel	Known
	11	117,088,082	rs2238005	T	C	0.061	0.118	0.014	2.71E-17	7.23E-01	45330	*PCSK7*	*PCSK7*	Known	Novel	Known
	12	51,783,420	rs10876169	A	T	0.410	0.039	0.007	1.85E-08	1.15E-01	44584	*GALNT6*	*GALNT6*	Novel	Novel	Known
	13	110,401,304	rs76944188	T	C	0.942	−0.090	0.015	5.45E-10	4.07E-01	45330	*IRS2*	*IRS2*	Novel	Novel	Novel
	15	45,395,901	rs75922593	G	GT	0.066	0.112	0.014	3.64E-15	5.67E-01	40837	*DUOX2*	*DUOX2*	Novel	Known	Known
	16	88,567,333	rs74035509	T	C	0.083	0.083	0.013	6.36E-11	6.39E-01	44584	*ZFPM1*	*ZFPM1*	Novel	Novel	Novel
	17	43,556,807	rs55925547	T	C	0.801	−0.057	0.009	2.46E-11	7.60E-01	43244	*PLEKHM1*	*AC003070.1/AC091132.2/LINC02210*	Novel	Novel	Novel
	17	57,925,649	rs1292072	A	G	0.794	−0.048	0.008	7.13E-09	9.06E-01	45330	*RNU6-450P*	*RPS6KB1*	Novel	Known	Known
	17	76,401,328	rs1976703	T	C	0.493	0.039	0.007	1.57E-08	1.08E-01	41826	*PGS1*	*PGS1*	Novel	Novel	Novel
	19	4,502,282	rs13041	T	C	0.518	0.048	0.007	8.53E-12	8.38E-01	44584	*PLIN4*	*UBXN6*	Novel	Novel	Novel
	22	37,462,936	rs855791	A	G	0.434	0.134	0.007	2.70E-89	8.92E-01	45330	*TMPRSS6*	*TMPRSS6*	Known	Known	Known

Chromosomal positions are in GRCh37 assembly. Beta estimates are per-allele dosage increase of the effect allele (EA). NEA indicates non-effect allele. EAF is EA frequency. SE, standard error. ‘Locus novelty’ indicates whether the variant is within a 500-Kb window from a previously reported genetic variant associated with other iron traits^7–9^; loci that are novel with respect to previous GWASs of hepcidin and sTfR^5,6^ are indicated in the ‘Locus novelty (hepcidin and sTfR)’ column. ‘Variant novelty’ indicates whether a variant is in moderate or high linkage disequilibrium (*r*^2^ ≥ 0.2) with a previously reported genetic variant associated with iron traits^7–9^. Variants that are novel according to both criteria across all iron traits are highlighted in red on grey background. Candidate genes are assigned based on colocalization, manual curation and proximity (see Supplementary Methods, Supplementary Data 7 and Supplementary Data 8).

Among 17 of the 52 sentinel variants, or their strong proxies (*r*^2^ > 0.7) at novel loci (Supplementary Data 3), 7 were missense, 6 were intronic, 2 were intergenic and 2 were downstream (Supplementary Data 5). Two of these variants at novel loci had a minor allele frequency (MAF) of <0.01 and both were associated with sTfR (rs143437464, intronic, and rs200307986, missense, near *TMEM181*).

Phenome-wide scans using PhenoScanner v.2 showed associations of multiple variants with a wide array of phenotypic traits across multiple domains. In addition to associations with haematologic traits (e.g., haemoglobin concentration, erythrocyte count), we also observed strong associations with traits relating to the cardiovascular system, autoimmune activity, infectious diseases, respiratory and hepatorenal function. Taken together, these results indicate involvement in multiple biological functions across several human body systems for nearly all the genetic variants associated with hepcidin and/or sTfR concentrations (Supplementary Data 6).

**Fig. 3:**
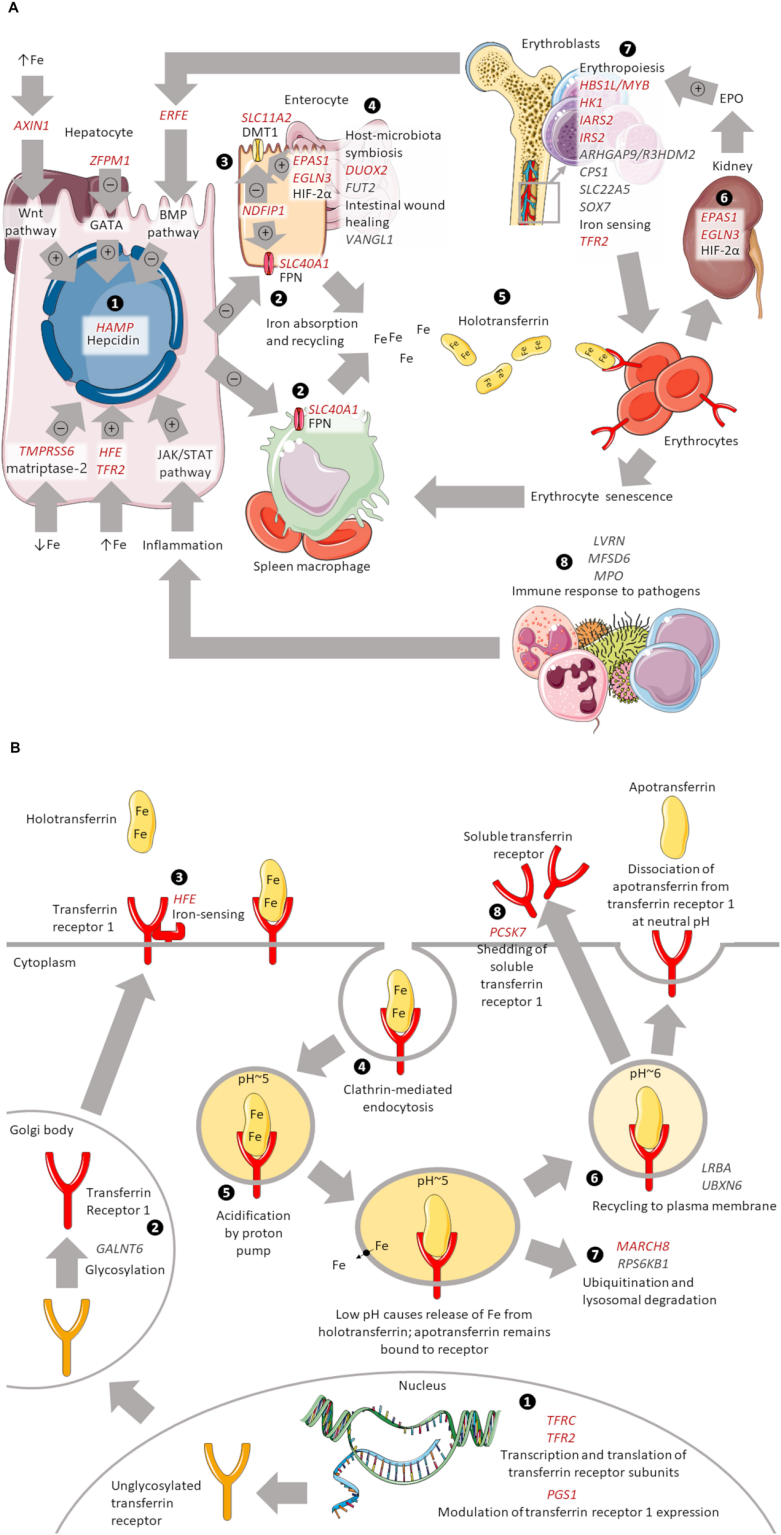
Established and potential candidate genes mapped to variants associated with hepcidin or sTfR: summary of their role and contextual information. **A** This figure summarises the genes mentioned in Table 2 of this study, as well as other iron-homoeostasis genes provided for contextual information. Genes with an established role in iron homoeostasis are shown in red and italic; genes with a potential role are presented in dark grey and italic. Relevant references to other studies are included in Supplementary Data 8 ❶ Hepcidin is tightly regulated by several pathways. *TMPRSS6, ERFE* (via the BMP pathway), and *ZFPM1* suppress hepcidin expression in hepatocytes. *HFE*, *TFR2*, the Wnt pathway, and the JAK/STAT pathway increase hepcidin expression. Activation of the Wnt pathways is observed in iron overload, with involvement of *AXIN1*. Activation of JAK/STAT signalling has been proposed as a possible link between inflammation and iron homoeostasis. ❷ In presence of iron abundance, hepcidin suppresses function of ferroportin (FPN), an iron transporter coded by *SLC40A1* that mediates dietary intestinal iron uptake and iron recycling by macrophages from senescent erythrocytes. *NDFIP1* prevents degradation of ferroportin in vitro. ❸ Hypoxia-inducible factor 2α (HIF-2 α), coded by *EPAS1* and regulated by *EGLN3*, also controls duodenal iron absorption by promoting the expression of divalent metal transporter 1 (DMT1), coded by *SLC11A2*, on the luminal side of enterocytes. *NDFIP1* regulates DMT1 expression in mice. *EGLN3* hydroxylates key prolyl residues on HIF-2α, providing a recognition motif for its degradation. ❹ Several genes appear relevant to intestinal iron absorption: (i) *DUOX2* regulates interactions between the intestinal microbiota and the mucosa to maintain immune homoeostasis in mice, which likely enables intestinal iron absorption; (ii) *FUT2* codes for fucosyltransferase 2, an enzyme responsible for maintaining host-microbiota symbiosis via fucosylation of intestinal epithelial cells; (iii) *VANGL1* encodes a protein involved in mediating intestinal trefoil factor-induced wound healing in the intestinal mucosa. ❺ Iron released through ferroportin is bound to iron carrier transferrin (referred to as apotransferrin when not bound to iron), forming iron-loaded transferrin (holotransferrin), which delivers iron to most cells, especially erythrocytes. ❻ In presence of hypoxia, raised levels of HIF-2 α result in increased erythropoietin (EPO) production. ❼ EPO stimulates erythropoiesis, which is also modulated by several genes involved in erythroblast proliferation and differentiation: (i) the *HBS1L/MYB* intergenic region regulates erythroid cell proliferation, maturation, and foetal haemoglobin expression; (ii) *HK1* mutations lead to haemolytic anaemia via hexokinase deficiency, which in turn likely affects erythropoiesis; (iii) *IRS2* expression plays a role in erythroid cell differentiation through binding to cellular receptors involved in normal haematopoiesis; (iv) *ARHGAP9* regulates adhesion of haematopoietic cells to the extracellular matrix, which can influence their localisation and differentiation potential, and *R3HDM2* has been mapped to haemoglobin and red blood cell traits in large-scale GWASs; (v) *CPS1* is directly related to glycine, which is an essential requirement for haem synthesis; (vi) *SLC22A5* is involved in the active cellular uptake of carnitine, which stimulates erythropoiesis; (vii) *SOX7* blocks differentiation of hematopoietic progenitors to erythroid and myeloid lineages. In erythroblasts, *TFR2* is a sensor of holotransferrin, and is thought to protect against excessive erythrocytosis in the presence of iron deficiency. ❽ Finally, the immune response to external pathogens, which compete for iron, may also influence overall iron availability. Among the genes identified, *LVRN* may play a role in the synthesis of defensins and defensin-like peptides such as hepcidin, potentially contributing to iron homoeostasis via immune response; (ii) *MFSD6* recognises major histocompatibility complex type I (MHC-I) molecules and mediates MHC-I restricted killing by macrophages; (iii) *MPO* catalyses the production of hypohalous acids, primarily hypochlorous acid in physiologic situations, and other toxic intermediates that greatly enhance microbicidal activity. Images from Servier Medical Art (https://smart.servier.com), licensed under a Creative Commons Attribution 3.0 Unported (CC BY 3.0) Licence. **B** This figure summarises the genes mentioned in Table 2 of this study, as well as other iron-homoeostasis genes provided for contextual information. Genes with an established role in transferrin receptor synthesis, recycling, or degradation are shown in red and italic; genes with a potential role are presented in dark grey and italic. Relevant references to other studies are included in Supplementary Data 8. ❶ *TFRC* codes for transferrin receptor 1, which is constitutively expressed in most cells, especially erythrocytes. *TFR2* codes for transferrin receptor 2, linked to iron sensing and maintenance of body iron homoeostasis. *PGS1* is involved in the synthesis of cardiolipin, a phospholipid of mitochondrial membranes implicated in the regulation of transferrin receptor expression. ❷ After O-linked glycosylation, possibly mediated by the protein product of *GALNT6*, transferrin receptor 1 is expressed on the external surface of the cytoplasmic membrane. ❸ *HFE* interactswith transferrin receptor 1, facilitating cellular iron-sensing function and playing an important part in the regulation of hepcidin expression in response to body iron status. ❹ Iron-loaded transferrin (holotranferrin) binds to the receptor and the complex is internalised through clathrin-mediated endocytosis. ❺ A proton pump acidifies the endosome, which causes release of iron from holotransferrin; iron-deprived transferrin (apotransferrin) remains bound to its receptor. ❻ The endosome is usually recycled to the plasma membrane, a process likely regulated by (i) *LRBA*, known to influence recycling of cytotoxic T-lymphocyte-associated protein 4 (CTLA-4) via the classical recycling pathway used by receptors such as transferrin and (ii) *UBXN6*, which negatively regulates the adenosine triphosphate (ATP) hydrolytic activity of valosin containing protein (VCP), an ATP-driven segregase; VCP depletion delays transferrin receptor recycling. ❼ At neutral pH, apotransferrin dissociates from transferrin receptor and is ready to bind to free iron. The transferrin receptor may also be ubiquitinated and directed to lysosomal degradation, which is mediated by *MARCH8*, a membrane-associated zinc-finger factor, and, possibly, also by *RPS6KB1*, a protein kinase involved in the mammalian target of rapamycin-protein S6 kinase (mTOR-S6K) pathway, which is implicated in the degradation of transferrin receptor 1. ❽ Finally, *PCSK7* mediates the shedding of soluble transferrin receptor (sTfR) from the transferrin receptor. When iron availability is limited, sTfR levels increase at least in part by downregulating expression of *PCSK7* or neighbouring genes. Images from Servier Medical Art (https://smart.servier.com), licensed under a Creative Commons Attribution 3.0 Unported (CC BY 3.0) Licence.

We mapped the 52 sentinel variants to 43 non-overlapping loci based on the nearest gene, of which 16 were associated with hepcidin and 27 with sTfR. We used colocalization with expression and protein quantitative trait loci to guide the selection of putative causal genes, in combination with evidence from functional studies (Supplementary Information, pp 7–8). Among the 16 candidate genes associated with hepcidin, we were able to annotate 14 putative causal genes based on either biology or a combination of colocalization and biology (‘biologically plausible genes’), one gene based on colocalization only and one gene based on vicinity to the sentinel variant (Supplementary Data 7, Supplementary Data 8). Biologically plausible genes were involved in hepcidin synthesis (*HAMP*), iron-sensing and hepcidin modulation (*AXIN1*, *HFE*, *TMPRSS6*), iron absorption and recycling (*DUOX2*, *FUT2*, *SLC11A2*, *SLC40A1*), reaction to hypoxia and haematopoiesis (*ARHGAP9/R3HDM2*, *EGLN3*, *IARS2*, *SOX7*), and immune reaction to pathogens (*LVRN*, *MPO*) (Fig. 3A). Of these, two putative causal genes (*ARHGAP9/R3HDM2* and *LVRN*) were not previously associated with iron traits. Among the 27 candidate genes annotated for sTfR, we were able to identify 19 biologically plausible genes (Supplementary Data 8), including genes involved in transferrin receptor synthesis, modulation, transport, degradation, recycling and shedding (*GALNT6*, *MARCH8*, *PCSK7*, *PGS1*, *RPS6KB1*, *TFRC*, *TFR2*, *UBXN6*), iron-sensing and hepcidin modulation (*HFE*, *TMPRSS6*, *ZFPM1*), intestinal iron absorption (*DUOX2*, *NDFIP1*), erythropoiesis (*CPS1*, *HBS1L/MYB*, *HK1*, *IRS2*, *SLC22A5*), and immune response (*MFSD6*) (Fig. 3A, B). Of these, 8 putative causal genes (*IRS2*, *MARCH8*, *MFSD6*, *NDFIP1*, *PGS1*, *SLC22A5, UBXN6* and *ZFPM1*) were at loci previously not reported in GWASs of iron traits.

### Putative causal effects of iron-related loci and iron status on disease outcomes and biomedical traits

**Fig. 4:**
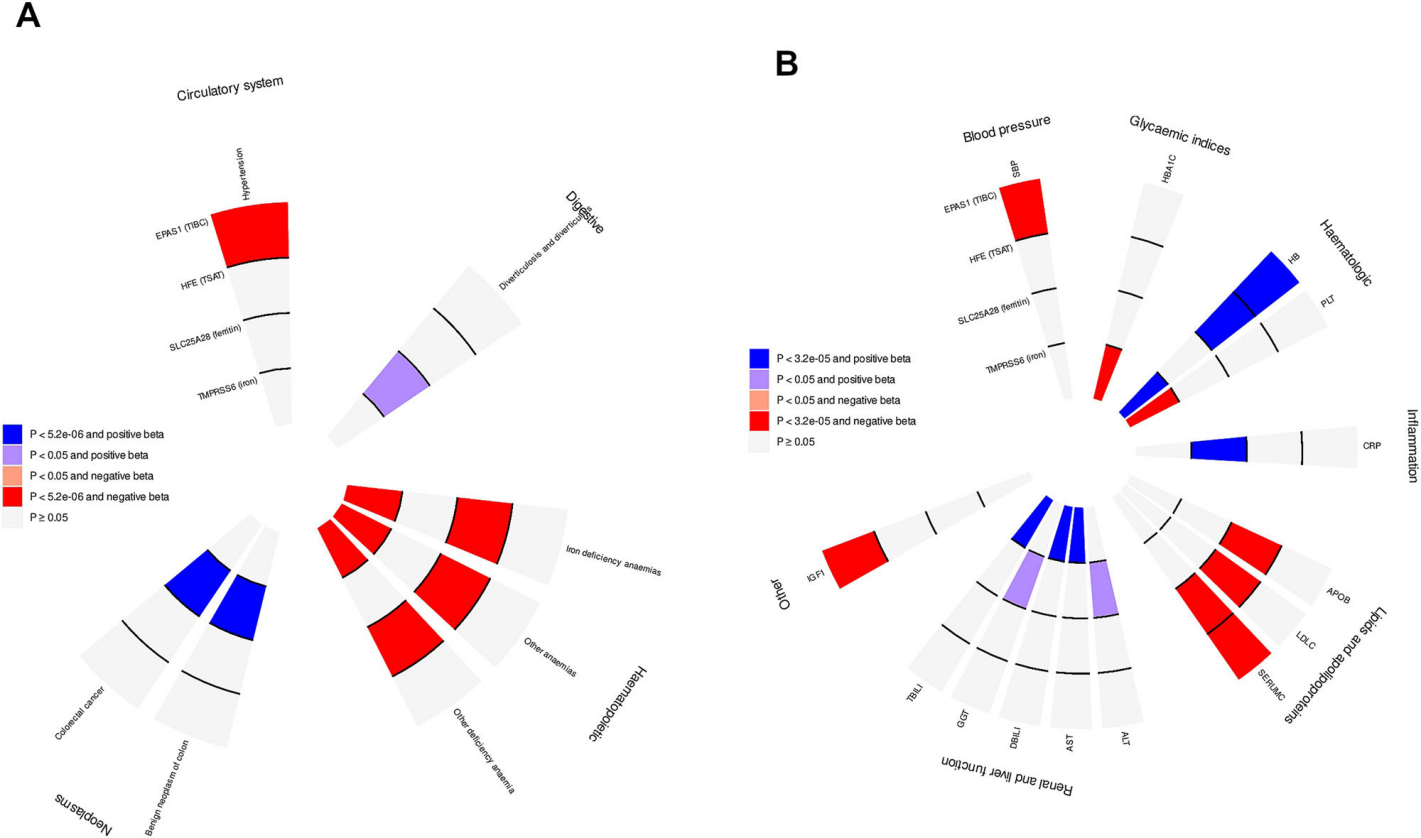
Putative causal effects of genetically predicted iron-related loci: Bonferroni-significant and nominal associations (null findings not presented). **A** Locus-based MR associations with disease outcomes in up to 1,469,361 deCODE, FinnGen, MVP, and UK Biobank participants. Only loci that are associated (*P* < 5.2 × 10^−6^) with at least one disease and have suggestive evidence of colocalization are shown. The terms in parenthesis indicate the trait that has been used for rescaling. **B** Locus-based MR associations with biomedical traits in up to 854,977 MVP and UK Biobank participants. Only loci that are associated with at least one disease outcome are shown. The terms in parenthesis indicate the trait that has been used for rescaling.

To mitigate pleiotropy and collider bias when defining instruments for MR analysis, we first collated 197 genetic variants associated with either hepcidin or sTfR in this study, and with serum iron, ferritin, TSAT or TIBC in a previous study^9^ (Fig. 1). We then removed variants affected by horizontal pleiotropy (i.e. influencing non-iron traits via pathways not mediated by iron traits, such as *ABO*), indirect vertical pleiotropy (i.e. influencing iron traits via pathways not mediated by iron traits, such as *F5*) and affected by collider bias (which can result in spurious genetic associations and invalid MR instruments) (Supplementary Data 9). For each non-pleiotropic and non-collider-biased variant associated with iron traits, we defined a 200 Kb region around its putative causal gene, selected conditionally independent variants using GCTA-COJO with summary statistics for the most strongly associated iron trait (Supplementary Data 10; Supplementary Data 11), and then performed *cis*-MR (i.e. locus-specific MR rescaled by genetic associations with iron traits) and colocalization. We found Bonferroni-significant log-linear associations of 19 loci with 47 diseases in 1,492,717 deCODE, FinnGen, MVP, and UKBB participants (Supplementary Fig. 1A; Supplementary Data 12). Of these, we found evidence of colocalization for four loci and six diseases (Fig. 4A; Supplementary Fig. 1B; Supplementary Data 12; Supplementary Data 13), highlighting the usefulness of this method in addressing residual genetic confounding. *HFE* (rescaled by TSAT) and *TMPRSS6* (iron) were strongly associated with inverse risk of iron-deficiency anaemia. We also found that *EPAS1* (TIBC) was inversely associated with hypertension and that *SLC25A28* (ferritin) was positively associated with colorectal cancer and benign neoplasm of colon; however, no other iron-related loci were associated and colocalized with these diseases, suggesting that these effects may be driven by horizontal pleiotropy. These four loci were associated with multiple biomedical traits, showing evidence of positive association of *HFE*, *TMPRSS6* and *EPAS1* with haemoglobin and inverse associations of *EPAS1* and *HFE* with total cholesterol, suggesting that iron may play a role in affecting these traits via these loci, as well as several other associations of isolated loci with glycaemic, inflammatory, hepatorenal and other traits (Fig. 4B; Supplementary Fig. 2; Supplementary Data 12).

**Fig. 5:**
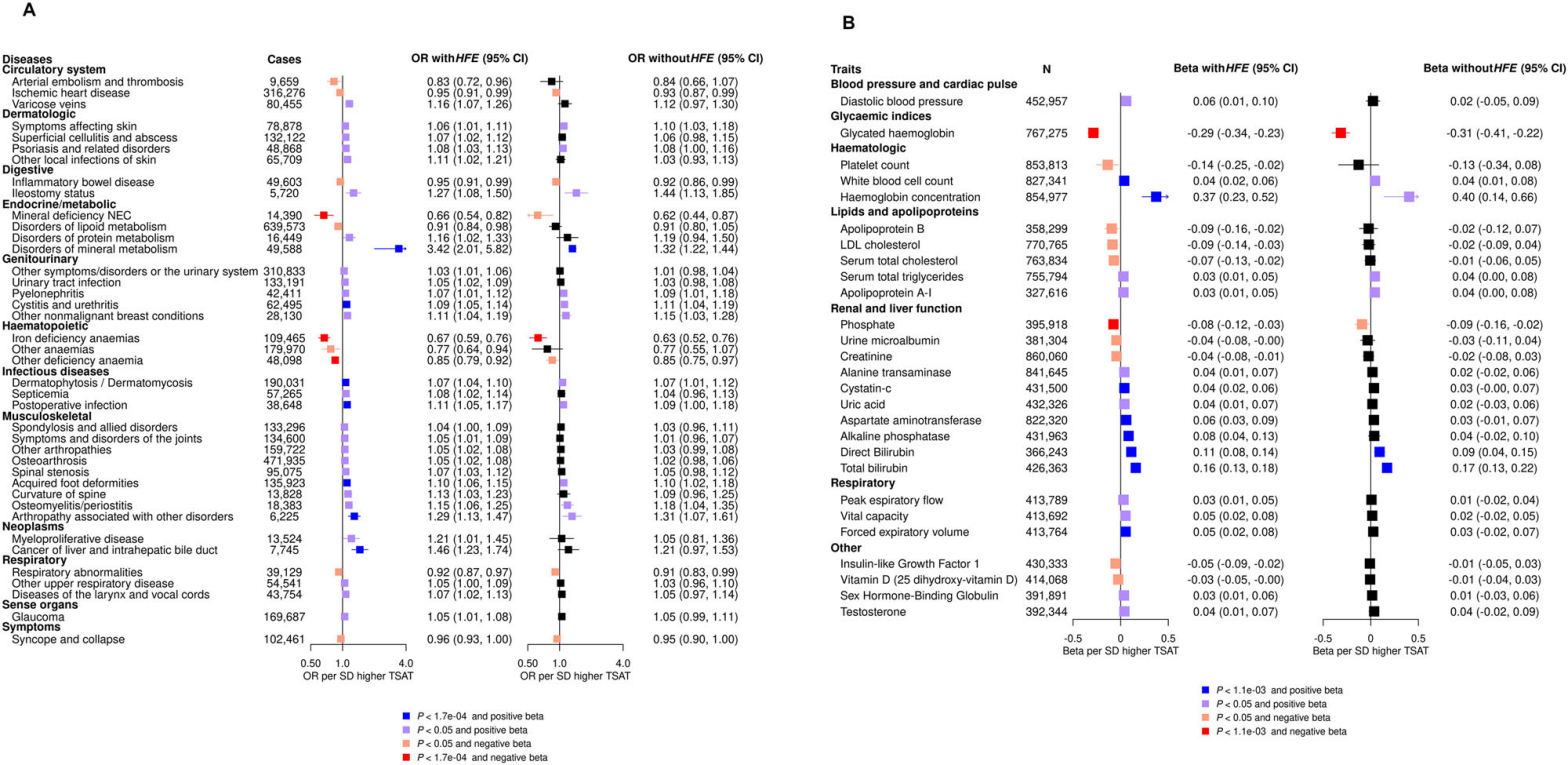
Putative causal effects of genetically predicted systemic iron status: Bonferroni-significant and nominal associations (null findings not presented). **A** MR associations of systemic iron status with disease outcomes in up to 1,492,717 deCODE, FinnGen, MVP, and UK Biobank participants. The estimates are expressed in odds ratio per one standard deviation (SD) higher transferrin saturation (TSAT) with confidence intervals shown between brackets. The plot shows estimates with the pC282Y variant in *HFE* (left-hand Forest plot) and without that variant (right-hand Forest plot), presenting diseases that have MR point estimates with the same direction in all the biobanks included in the meta-analysis. The instrument was generated using six variants mapped to *ERFE*, *HAMP*, *HFE*, *SLC25A37*, *TFR2* and *TMPRSS6* not affected by horizontal pleiotropy, indirect vertical pleiotropy, or collider bias and that: (i) were associated (*P* < 5 × 10^−8^) with at least one trait; (ii) were nominally associated (*P* < 0.05) with all the other iron traits except for hepcidin (as its levels are influenced by systemic iron status); and (iii) displayed a direction of association consistent across all traits. **B** MR associations of systemic iron status, using the same instrument, with biomedical traits in up to 860,060 MVP and UK Biobank participants. The estimates are expressed in mean change (beta) per one SD higher TSAT. The plot shows estimates with the pC282Y variant in *HFE* (left-hand Forest plot) and without that variant (right-hand Forest plot), presenting traits that have MR point estimates with the same direction in all the biobanks included in the meta-analysis.

We then generated a polygenic instrument of systemic iron status composed by six variants mapped to *ERFE*, *HAMP*, *HFE*, *SLC25A37*, *TFR2* and *TMPRSS6* (Supplementary Data 11), that were not affected by horizontal pleiotropy, indirect vertical pleiotropy, or collider bias and that: (i) were associated (*P* < 5 × 10^−8^) with at least one trait; (ii) were nominally associated (*P* < 0.05) with all the other iron traits except for hepcidin (as its levels are influenced by systemic iron status); and (iii) displayed a direction of association consistent across all traits (e.g., positive for iron, ferritin, TSAT and negative for TIBC and sTfR). To reduce the impact of study-specific estimates that may disproportionately affect meta-analytic estimates, we present Bonferroni-significant and nominal results for diseases and traits having MR estimates with the same direction (regardless of their *p* value) in all the studies included in the meta-analysis. In an agnostic analysis of 292 disease outcomes in 1,492,717 deCODE, FinnGen, MVP and UKBB participants, we found four expected Bonferroni-significant log-linear associations of genetically predicted higher iron status with lower risk of mineral deficiency (a cluster of conditions that includes iron-deficiency), iron-deficiency anaemia and other deficiency anaemia, and with higher risk of disorders of mineral metabolism (a cluster of conditions that includes haemochromatosis). We also found six Bonferroni-significant associations with higher risk of cystitis and urethritis, dermatophytosis/dermatomycosis, postoperative infection, acquired foot deformities, arthropathy associated with other disorders, and liver cancer (Fig. 5A; Supplementary Data 14). Genetically predicted systemic iron status was also nominally associated with multiple clinical outcomes spanning various domains: circulatory, dermatologic, digestive, endocrine/metabolic, genitourinary, haematopoietic, infectious-disease, musculoskeletal, neoplasms, respiratory, sense organs and symptoms. Sensitivity analyses showed general robustness of findings when using MR Egger regression and the weighted median estimator (Supplementary Fig. 3). Although some between-variant heterogeneity was present for specific outcomes (e.g., disorders of mineral metabolism and other anaemias; Supplementary Data 14), MR Egger intercepts generally showed no evidence of residual horizontal pleiotropy (Supplementary Fig. 3). However, when removing the pC282Y variant in *HFE* from the polygenic instrument, most of these associations did not reach significance, except for iron-deficiency anaemia and iron-metabolism disorders, suggesting that these associations may be largely driven by *HFE*, although reduced statistical power might play a role in widening confidence intervals (Fig. 5A; Supplementary Data 14). We also found ten Bonferroni-corrected log-linear associations of genetically predicted iron concentration with multiple biomedical traits in up to 860,060 MVP and UKBB participants across the following domains: glycaemic indices, haematologic, hepatorenal function and respiratory (Fig. 5B; Supplementary Data 14). Sensitivity analyses showed general robustness of findings when using MR Egger regression and the weighted median estimator (Supplementary Fig. 4). Three associations persisted after removing the pC282Y variant in *HFE*: an inverse association of genetically predicted iron concentration with glycated haemoglobin (HbA1c), and positive associations with direct bilirubin and total bilirubin.

## Discussion

In this meta-analysis of 12 original GWASs of over 90,000 participants, we identify 43 loci associated with hepcidin and sTfR concentrations, including 15 novel loci not previously associated with iron biomarkers. Through manual curation and colocalization, we mapped the new loci to several putative genes, suggesting potential roles in iron-trait expression (*PGS1*, *ZFPM1*), erythropoiesis (*ARHGAP9/R3HDM2*, *IRS2*, *SLC22A5*), immune response (*LVRN*, *MFSD6*) and cellular trafficking (*MARCH8*, *NDFIP1*, *UBXN6*), although functional confirmation of candidate loci and variants is required. In the first large-scale MR study, involving over 1.4 million participants from four studies, we investigated the causal effects of iron-related pathways and systemic iron status on over 292 major health outcomes and conditions. Our findings showed that higher genetically predicted systemic iron status was inversely associated with mineral deficiency and anaemia-related disorders, suggesting that, aside from anaemia, iron deficiency is unlikely to be associated with major diseases explored in this analysis. Conversely, higher systemic iron status was positively associated with a range of conditions, including genitourinary, musculoskeletal, infectious, and neoplastic diseases. These associations attenuated after using the polygenic instrument without the pC282Y variant in *HFE*, which increases the risk for iron overload and, in its homozygous form, accounts for the majority of hemochromatosis cases^10^, suggesting that they might be driven by very high iron levels.

We identified two new putative causal loci associated with hepcidin, *ARHGAP9/R3HDM2,* and *LVRN*, whose role in hepcidin metabolism is not yet fully understood. *ARHGAP9/R3HDM2* is involved in regulating adhesion of hematopoietic cells to the extracellular matrix, which can influence their localisation and differentiation potential^31^. Erythropoietic expansion, in turn, depresses hepcidin production^1^. *LVRN* codes for laeverin, an aminopeptidase cleaving N-terminal amino acids of peptides^32^. β-defensins, implicated in innate immunity, feature two sites under positive selection in the N-terminal region that may contribute to their functional diversity in primates^33^. *LVRN* may play a role in the synthesis of defensins and could affect hepcidin through an inflammation-mediated pathway. We also found biologically plausible candidate genes for 8 of the 13 new loci mapped to sTfR-associated sentinel variants. Of these, *IRS2*, *MARCH8* and *NDFIP1* appear to have a more established role in iron biology. *IRS2* is involved in erythroid cell differentiation^34^, which in turn affects iron availability and transferrin receptor presentation^1^, *MARCH8* mediates the lysosomal degradation of the transferrin receptor^35^, and *NDFIP1* regulates iron import^36,37^. Five additional genes mapped to new sTfR-associated variants likely play a role in iron homoeostasis. *MFSD6* contributes to shaping the gut microbiome^38^, possibly increasing iron availability; additionally, as it is an MHC-I receptor homologue^39^, could potentially compete with transferrin receptor 1 for interacting with *HFE*, an MHC-I homologue^40^. *PGS1* could affect expression of transferrin receptor 1 via cardiolipin^41^. *SLC22A5* is involved in the cellular uptake of carnitine^42^, which stimulates erythropoiesis^43^. *UBXN6* regulates endosome recycling to the plasma membrane^44^, likely mediating transferrin receptor presentation and sTfR concentration. Finally, *ZFPM1* suppresses GATA-mediated activation of hepcidin expression^45^, although its connection with sTfR remains unclear. It is worth noting that GWASs rely on population-level natural variation, which can lead to both overstatement and understatement of the role of individual modulators due to their natural variants being over- or underrepresented in human genomes. At the population level, the impact of common variants that have a relatively minor role in iron biology (e.g., *HFE* variants) may be overstated, whereas the impact of rarer variants with a major effect on iron homoeostasis (e.g., *HAMP* variants) may be understated.

In addition to the expected association with anaemia-related phenotypes, the only other Bonferroni-significant associations of individual biological pathways that persisted in colocalization were *EPAS1* with hypertension and *SLC25A28* with colorectal cancer and benign neoplasm of colon. However, no other loci were associated with these diseases, suggesting that mediation through pathways specific to these loci (rather than through iron-related pathways) is more likely. For example, *EPAS1* codes for hypoxia-inducible factor 2-alpha, a transcription factor that contributes to maintaining oxygen homoeostasis in response to hypoxia through activation of several biological pathways, such as raising norepinephrine levels^46^, in addition to iron absorption and transport.

The finding that multiple positive associations of systemic iron status with diseases attenuated after removing the pC282Y variant in *HFE* constitutes one of the key results of this study, suggesting that these associations may be driven by extreme iron overload and that moderate iron overload may be unlikely to affect health outcomes other than mineral metabolism disorders. In keeping with this interpretation, the strongest association with non-haematologic and non-metabolic disease outcomes was with greater risk of liver cancer, which is consistent with reports showing associations of the pC282Y variant with liver cancer^11^, and mentioning hepatocellular carcinoma as a common manifestation of hemochromatosis^10^. The second-strongest association with non-haematologic and non-metabolic disease outcomes was with arthropathy, which is also consistent with reports of associations of pC282Y with osteoarthritis^11^ and mentioning joint pain as a common symptom of hemochromatosis^10^. It is also possible, however, that the wider confidence intervals observed after removing the pC282Y variant in *HFE* may be due to reduced statistical power.

We also found positive associations of systemic iron status with greater risk of dermatophytosis/dermatomyositis, postoperative infection and cystitis/urethritis, broadly consistent with previous research that showed associations with skin^20^ and bacterial^24^ infections. It is worth noting that we did not observe associations with heart failure, which is consistent with a recent randomised trial^30^ but in disagreement with previous trials^26–29^. We did find an inverse nominal association with ischaemic heart disease, in keeping with previous MR studies^20,21^ but in disagreement with some observational evidence^12–15^. We also found a strong inverse association of genetically predicted systemic iron status with HbA1c, which may reflect greater erythrocyte turnover driven by iron excess^47^. Our findings reinforce previous warnings about interpreting HbA1c concentration in patients with iron-status imbalances^47^, leading to potential underestimation of type-2 diabetes in individuals with iron overload.

Our investigation has several strengths. Firstly, the GWASs of hepcidin and sTfR have the largest sample size collected to date for genomic studies of these traits, enabling the discovery of the first genetic loci associated with hepcidin and multiple new loci associated with sTfR. Secondly, to assess the biomedical consequences of iron-altering biological pathways and systemic iron status, we employed an MR design on a wide range of major clinical outcomes, which reduces the impact of common sources of bias present in observational studies, such as confounding and reverse causality. It is, however, possible that this analysis may not capture rarer conditions and diseases not included in the curated list of health outcomes. Finally, by leveraging the largest sample size in an iron MR conducted to date, we had very good (>90%) statistical power for the majority of the 292 outcomes included in our analysis.

However, there are also some limitations. Firstly, in our GWASs we focused on variants with MAF ≥ 0.001. Despite identifying some associations with rare variants, the role of rarer variants remains to be fully investigated. Secondly, the focus on European ancestry participants limits the generalisability of these findings, particularly in countries and ethnicities where the majority of the burden of iron deficiency lies. Thirdly, methodological differences in the GWASs, such as diverse adjustments for covariates and varying limits of detection, may have reduced homogeneity in meta-analysis, despite all GWASs adhering to the same analysis plan. Fourthly, our phenome scans demonstrated the extensive influence of genetic pleiotropy on iron traits. This study, however, utilises a systematic approach to reduce its impact on MR analyses by selecting only variants that are likely non-pleiotropic, and complementing locus-based MR with colocalization analysis to further reduce the impact of genetic confounding. Finally, this study assumes additive genetic associations of instrumental variables with iron traits, potentially missing between-variant interactions, and focuses on the linear effects of iron, potentially overlooking non-linear associations.

Taken together, this study increases knowledge of iron homoeostasis and its biomedical consequences in humans, suggesting that long-term exposure to higher iron levels is likely associated with lower risk of anaemia-related disorders and higher risk of genitourinary, musculoskeletal, infectious and neoplastic diseases.

## Methods

### Genetic discovery study of emerging iron traits

All studies included in the GWASs of hepcidin and sTfR followed the same analysis plan, described in the Supplementary Information, p 2. The characteristics of the cohorts included in this study are described in the Supplementary Information, pp 2–6 and in Supplementary Data 1.

We established a data-management and quality-check pipeline for study-specific GWAS results (Supplementary Information, p 6). We performed fixed-effect meta-analysis in METAL using the SCHEME STDERR command for all variants with MAF ≥ 0.001. After removing variants available in only one study and with a combined sample size lower than 20,000 participants, we estimated SNP-based heritability and genomic inflation factor using LDSC v. v1.0.1 (Supplementary Information, p 6).

To identify genetic variants independently associated with either hepcidin or sTfR concentration, we performed approximate conditional analysis using stepwise algorithm (‘--cojo-slct’) in gcta64 v. 1.26.0 on the whole genome. We selected all single nucleotide polymorphisms (SNPs) with *P* < 5 × 10^−8^ in the meta-analysis of each trait and we specified the same *p* value for the ‘--cojo p’ argument, to ensure that conditionally independent SNPs were still genome-wide significant. Consistently with a previous study^48^, we then clumped all resulting GWAS variants using PLINK v1.9 to include only independent variants not in linkage disequilibrium (LD) with one another within a 1 Mb window (*r*^2^ < 0.01). We performed both GCTA and clumping using LD information from 41,845 unrelated participants in the INTERVAL study.

We provisionally mapped conditionally independent (sentinel) variants to their nearest gene using PhenoScanner v.2, a phenome scan tool that includes mapping to nearest gene retrieved from BEDOPS v. 2.4.26, with additional manual verification using the Ensembl genome browser (https://grch37.ensembl.org/). We assessed the novelty of association using two approaches. Firstly, we defined a ‘novel variant’ as any SNP (or its *r*^2^ ≥ 0.7 proxy) not associated with any iron traits in previous genome-wide studies^6–9^. Secondly, we defined as ‘novel locus’ any genomic locus (within 500 Kb window from each independent variant) not including one or more variants discovered in previous studies^6–9^.

We used Ensembl Variant Effect Prediction to obtain information for several measures of functional consequence for each sentinel variant and their proxy variants (*r*^2^ > 0.7) (Supplementary Information, pp 6–7)^49^. We conducted phenome scans drawing on the curated database of >65 billion genetic summary statistics available in PhenoScanner v.2 (Supplementary Information, p 7). We estimated genetic correlation using summary statistics from the present study (meta-analysis of hepcidin and sTfR concentration) and from a previous GWAS of conventional iron traits^9^, using LDSC with the ‘--rg’ argument. We estimated phenotypic Pearson correlation and its precision in up to 40,197 INTERVAL participants.

To map sentinel variants to candidate genes, we used a combination of manual curation and colocalization with expression and protein quantitative data. We first mapped the above-defined conditionally independent and uncorrelated GWAS signals to their nearest gene and then collapsed overlapping genes within 200 Kb from each other. The process was performed independently for hepcidin- and sTfR-associated variants. This led to the definition of 43 non-overlapping loci. Of these, 21 already had a biologically plausible candidate gene (e.g., *HFE*, *TMPRSS6*, *HAMP*, *TFRC*). For the remaining 22 loci, we performed conditional colocalization in Sum of Single Effects (SuSiE) v. 0.11.92 and Coloc v. 5.1.0, following the procedure described in Supplementary Information, pp 7–8. Locus-specific information on our candidate gene mapping process, including a summary of our manual curation, is available in Supplementary Data 8.

### Locus-based and polygenic phenome-wide MR analysis

We collated 197 genetic variants (189 after deduplication) associated with iron traits, of which 52 associated with either hepcidin or sTfR (in the present study) and 145 associated with serum iron, ferritin, TSAT and TIBC (reported previously^9^). Because iron is involved in multiple biological processes, genetic variants associated with iron traits are often associated also with other traits (pleiotropy). This may lead to biased MR associations if genetic associations with iron traits are distinct (horizontal pleiotropy) or mediated by a non-iron trait (indirect vertical pleiotropy). To reduce the impact of horizontal and indirect vertical pleiotropy in our analysis, we performed phenome scans in MR Base and retained 57 genetic variants mapped to genes that (i) included only sentinel variants associated with iron traits or iron-related traits (such as haemoglobin concentration and erythrocyte count); (ii) affected iron homoeostasis directly and not via a non-iron phenotype (e.g., variants mapped to *HFE*, *TMPRSS6* and *HAMP*). We then assessed potential collider bias by comparing the genetic associations with and without adjustment for covariates that may result in collider bias (e.g., body mass index, smoking and others) in up to *N* = 40,197 INTERVAL participants (Supplementary Information, p 8). This analysis showed very high correlation (*r*^2^ ≈ 1.00) between estimates of these two approaches. All effect estimates had the same direction in the two models, apart from two variants (rs79694859 and rs10804630) that we removed from our list of MR instruments, leaving 55 variants for further analysis (Supplementary Data 9).

To select locus-based MR instruments, firstly, we mapped these 55 variants to their most plausible or nearest genes as defined in their source GWAS, leading to 39 non-overlapping loci. To ensure better generalisability of associations with clinical outcomes, we further selected 33 (out of 39) loci including at least one variant available in all the studies involved in the MR (Supplementary Data 10). We performed stepwise approximate conditional analysis for the 200 Kb region around each variant’s mapped candidate gene (*P* < 10^−5^, *r*^2^ < 0.1) in gcta64 v. 1.26.0 using genetic summary statistics for the most strongly associated iron trait at each locus. This returned locus-based instruments for 33 loci with variance explained between <0.1%–4.1% (Supplementary Information, p 8; Supplementary Data 10; Supplementary Data 11). To select the polygenic MR instrument of systemic iron status, we filtered the above-mentioned 55 variants and included those that were: (i) associated (*P* < 5 × 10^−8^) with at least one iron trait, (ii) nominally associated (*P* < 0.05) with all the other iron traits considered; and (iii) with a direction consistent across all traits (e.g., positive for iron, ferritin, TSAT and negative for TIBC and sTfR; or the other way round) (Supplementary Fig. 5; Supplementary Data 11). Because hepcidin is influenced by systemic iron status and therefore it is difficult to disentangle whether genetic associations with hepcidin affect this trait directly or through other iron traits, we did not consider hepcidin associations in the definition of the polygenic instrument. We selected six variants for the polygenic instrument of systemic iron status mapped to *ERFE*, *HAMP*, *HFE*, *SLC25A37*, *TFR2* and *TMPRSS6*. In MR analysis, we rescaled the polygenic instrument by TSAT as it had the highest variance explained, 4.4%. We performed sensitivity analyses for key MR results utilising more liberal sets of polygenic instruments, illustrating the value of the 6-variant instrument in mitigating pleiotropy and heterogeneity (Supplementary Data 15). We estimated statistical power for this instrument, showing ≥90% power for ≥50% disease outcomes while assuming an OR of 1.5 (Supplementary Information, p 8; Supplementary Fig. 6). Because variants in *HFE* had the strongest genetic associations across all traits analysed (Supplementary Fig. 7), we performed a sensitivity analysis using a polygenic instrument without the *HFE* variant to identify MR association driven by *HFE*.

Before performing MR analysis, we estimated genetic associations of all instruments with health outcomes from deCODE, FinnGen data freeze 10 (R10), MVP and UKBB and with biomedical traits from MVP and UKBB in European-ancestry participants. We adjusted for age, sex (for non-sex-specific outcomes) and either the first 10 principal components of ancestry (FinnGen, MVP and UKBB) or county (deCODE). Information on the deCODE^50^, FinnGen^51^, MVP^52^ and UKBB^53^ cohorts is available elsewhere. We meta-analysed study-specific genetic associations using fixed-effects models in the ‘metafor’ R package. We defined 292 binary disease outcomes available in all four studies using a curated list of major phecodes available in the ‘PheWAS’ R package. To restrict our analysis to major health outcomes of interest, we discarded any sub-categories (i.e. phecodes with four or more characters), removed hereditary/poisoning-related/accident-related outcomes and those with less than 100 events in each study. The disease outcomes were grouped in the following domains: circulatory system, dermatologic, digestive, endocrine/metabolic, genitourinary, haematopoietic, infectious diseases, mental disorders, musculoskeletal, neoplasms, neurological, pregnancy complications, respiratory, sense organs, symptoms. We grouped biomedical traits in the following domains: blood pressure and cardiac pulse, glycaemic indices, haematologic, inflammation, lipids and apolipoproteins, renal and liver function, respiratory, other.

We performed univariable MR using the inverse-variance weighted method for each locus-based and polygenic instrument while accounting for between-variant correlation estimated in INTERVAL. We performed sensitivity analyses using MR Egger regression and weighted median estimator. We used fixed-effect models in locus-based analyses and random-effects models in polygenic analyses. We quantified between-variant heterogeneity using the I-squared statistic. To account for multiple testing, we used Bonferroni-corrected thresholds for all analyses. For locus-based analyses, these were *P* < 0.05/(33 × 292) (5.2 × 10^−6^) for diseases and *P* < 0.05/(33 × 47) (3.2 × 10^−5^) for traits. For polygenic analyses, the thresholds were *P* < 0.05/292 (1.7 × 10^−4^) for diseases and *P* < 0.05/47 (1.1 × 10^−3^) for traits. To reduce the impact of individual study-specific estimates that may disproportionately affect meta-analytic estimates, in the main figures we present Bonferroni-significant and nominal results for diseases and traits that have MR estimates in the same direction (regardless of their *p* value) in all the biobanks included in the meta-analysis, although all results are available in the Supplementary Data. Associations with *p* values below 0.05 but above the Bonferroni thresholds are described as ‘nominal associations’. For locus-based MR nominal associations, we performed colocalization analysis to remove associations chiefly driven by genetic confounding (Supplementary Information, p 8).

### Reporting summary

Further information on research design is available in the Nature Portfolio Reporting Summary linked to this article.

## Supplementary information

Supplementary Information

Description of Additional Supplementary Files

Supplementary Data 1–15

Reporting Summary

## Data Availability

GWAS summary statistics are publicly available through the NHGRI-EBI GWAS Catalogue (hepcidin: accession number GCST90451683; soluble transferrin receptor: accession number GCST90451684).
