# Supplementary material for: Novel loci and biomedical consequences of iron homoeostasis variation
Source: Commun Biol. 2024 Dec 6;7:1631. doi: 10.1038/s42003-024-07115-3 (PMC11624196; doi:10.1038/s42003-024-07115-3)
Supplement: Supplementary file 1 — Supplementary Information [file 42003_2024_7115_MOESM1_ESM.docx]

Supplementary Information

Contents

[Supplementary Methods 2](#_Toc184286116)

[Analysis plan of genome-wide association studies of hepcidin and sTfR 2](#_Toc184286117)

[Characteristics of individual genome-wide association studies of hepcidin and sTfR 2](#_Toc184286118)

[Data cleaning and quality checks 6](#_Toc184286119)

[Genome-wide summary statistics 6](#_Toc184286120)

[Variant annotation and effect prediction 6](#_Toc184286121)

[Phenome scans 7](#_Toc184286122)

[Colocalization and candidate gene mapping 7](#_Toc184286123)

[Assessing potential collider bias in MR instruments 8](#_Toc184286124)

[Variance explained by MR instruments 8](#_Toc184286125)

[Power calculations 8](#_Toc184286126)

[Colocalization analysis after locus-based MR 8](#_Toc184286127)

[Supplementary Results 9](#_Toc184286128)

[Genome-wide statistics, variance explained and genetic/phenotypic correlation 9](#_Toc184286129)

[Genetic associations of the 52 lead variants with iron traits 9](#_Toc184286130)

[Sensitivity GWASs adjusted for age and sex 9](#_Toc184286131)

[Sensitivity GWASs adjusted for C-reactive protein 9](#_Toc184286132)

[Supplementary Figures 11](#_Toc184286133)

[**Supplementary Figure 1**. Putative causal effects of iron-related loci on disease outcomes 12](#_Toc184286134)

[**Supplementary Figure 2**. Putative causal effects of iron-related loci on biomedical traits 13](#_Toc184286135)

[**Supplementary Figure 3**. Polygenic MR of disease outcomes, sensitivity analyses 14](#_Toc184286136)

[**Supplementary Figure 4**. Polygenic MR of biomedical traits, sensitivity analyses 16](#_Toc184286137)

[**Supplementary Figure 5**. Generation of polygenic MR instrument 18](#_Toc184286138)

[**Supplementary Figure 6**. Power estimation for the polygenic MR instrument 19](#_Toc184286139)

[**Supplementary Figure 7**. Association of locus-based scores with iron traits 20](#_Toc184286140)

[**Supplementary Figure 8**. Comparison of betas to investigate potential collider bias 21](#_Toc184286141)

[**Supplementary Figure 9**. Quantile-quantile plots and key genomic statistics for hepcidin and sTfR 22](#_Toc184286142)

[**Supplementary Figure 10**. Heatmap presenting genetic associations of hepcidin- and sTfR associated variants with iron traits 23](#_Toc184286143)

[**Supplementary Figure 11**. Sensitivity analyses adjusted for age and sex 24](#_Toc184286144)

[**Supplementary Figure 12**. Sensitivity analyses adjusted for C-reactive protein 25](#_Toc184286145)

[References 26](#_Toc184286146)

# Supplementary Methods

## Analysis plan of genome-wide association studies of hepcidin and sTfR

All studies included in the GWASs of hepcidin and sTfR followed the same analysis plan that required study investigators to account for covariates associated with variation in iron traits and followed a multi-step approach described previously.^1^ In the first step, sTfR and hepcidin were log-transformed as appropriate and all data points lying more than 4.5 interquartile range from the median were removed, based on the predicted number of outliers when assuming a normal marginal univariate distribution. In the second step, hepcidin and sTfR were regressed on as many covariates as possible from the following: age, sex and postmenopausal status, health and lifestyle (body mass index, alcohol consumption, smoking, ABO blood group, iron supplements, medications that may affect iron homeostasis, number of whole blood donations), seasonal (date of examination) and diurnal variation (time of blood draw), as well as technical variables (time between blood draw and processing, centre in multicentre studies, batch/plate number). In the final step, a GWAS was conducted on the inverse normalised residuals of the iron-related traits, while adjusting for the first five principal components of ancestry.

## Characteristics of individual genome-wide association studies of hepcidin and sTfR

The characteristics of the genetic cohorts included in this study and the methods of genome-wide association analyses are described in **Supplementary Table 1** and summarised below. Overall, two studies (FinDonor_1 and FinDonor_2) were conducted in Finnish participants, one in Icelandic participants (deCODE) and the rest in more admixed European-ancestry populations. The mean age ranged between 40 and 67 years, and the percentage of female participants ranged between 47% and 61%. Seven studies had hepcidin measurements (N=91,675) and six had sTfR measurements (N=45,330). Sample call rate thresholds ranged between 0.95 and 1.00. The minor allele frequency (MAF) threshold chosen by most studies was 0.01, except for DBDS and INTERVAL that had a lower threshold (0.001). Single nucleotide polymorphism (SNP) call rate thresholds ranged between 0.90 and 0.99, and the p-value for Hardy-Weinberg Equilibrium filtering ranged between 1.00E-04 and 1.00E-We232,571-654,966), imputation panels (most frequent: 1000G and HRC), imputation software (most common: Impute) and INFO cut-off (range: 0.4-0.9). Adjustment variable varied considerably, but all studies adjusted for age and sex, and all minus two (CROATIA_Vis and deCODE) adjusted for principal components of ancestry. The SNPs analysed ranged between 7,103,553-21,743,737 and the most frequently used analysis software was BOLT-LMM. Although GWASs followed a single analysis plan, variations in covariate adjustment may have contributed to between-study heterogeneity in the meta-analysis. Hepcidin was most frequently measured with ELISA kits and sTfR with immunoturbidimetric assays. These assays had detection limits, leading to not-missing-at-random censored values in phenotypes and potentially reducing statistical power. Additionally, variations in these detection limits across studies may have contributed to between-study heterogeneity.

**CHRIS (hepcidin).** The Cooperative Health Research In South Tyrol (CHRIS) study is a longitudinal, population-based study established in 2011 to investigate the genetic basis of common chronic conditions associated with human ageing, and their interaction with lifestyle and environmental factors in the general population of South Tyrol.^2^ The CHRIS study was approved by the Ethical Committee of the Healthcare System of the Autonomous Province of Bolzano (Südtiroler Sanitätsbetrieb/Azienda Sanitaria dell’Alto Adige), protocol no. 21/2011 (19 Apr 2011). After genotyping with the Illumina HumanOmniExpressExome platform, we imputed 654,042 variants using the Minimac / Michigan Imputation Server (<https://imputationserver.sph.umich.edu/index.html>), resulting in 17M variants available for association analysis. Hepcidin-25 was measured by liquid chromatography with tandem mass spectrometry using the following procedure: after treating samples by solid-phase extraction using Oasis hydrophilic-lipophilic balanced reversed-phase cartridges (Waters, Milan, Italy), high-pressure liquid chromatography was performed using an X-Terra MS C182.5m (Waters), and detection was obtained using a Triple Quad LC-MS/MS (Agilent Technologies, Santa Clara, CA, USA). Association analysis was performed using the EPACTS pipeline (Efficient and Parallelizable Association Container Toolbox) using the EMMAX algorithm (<https://genome.sph.umich.edu/wiki/EPACTS>).

**CROATIA_Vis (sTfR).** The CROATIA_Vis study includes 1008 Croatians, aged 18-93 years, who were recruited from the villages of Vis and Komiza on the Dalmatian island of Vis between 2003 and 2004.^3^ Participants underwent a medical examination and interview, and had their fasting blood drawn and stored for future analyses. We performed multiple biochemical and physiological measurements and collected participant medical history, as well as lifestyle and environmental exposures. All participants were volunteers and gave informed consent. The study received approval from the relevant ethics committees in Scotland and Croatia and complied with the tenets of the Declaration of Helsinki. We performed genotyping using Illumina HumanHap300v1 arrays and the Beadstudio - Gencall v3.0 calling algorithm. Imputation of 289,827 variants with Shapeit v2.r873 + duohmm software and HRC v1.1 reference panel yielded 11,489,434 variants available for association analysis. We measured sTfR using particle-enhanced immunonephelometry (Siemens, Germany). We performed association analysis using BOLT-LMM v 2.3.^4^

**DBDS (hepcidin).** The Danish Blood Donor Study (DBDS) is a cohort of 110,000 blood donors with mean age 41 (interquartile range: 29-50) recruited via the Danish blood banks infrastructure.^5^ The overarching purpose of the study is to enhance knowledge on factors influencing health, including the interaction between environmental and genetic factors. The project is approved by the Research Ethics Committees in relation to the following three protocols: The DBDS II (SJ-740), Genetics of healthy ageing (CVK-1700407), and the Family study on the genetics of healthy ageing (NVK-1803847). The project is approved by the Danish Data Protection Agency under the combined approval for health care research at The Capital Region of Denmark (P-2019-99). We measured hepcidin in plasma samples of 10,029 DBDS participants. After removing participants with measurements below the detection level, participants without sufficient covariate data and those without genotype data, 8985 participants were used in the analysis. Genotyping with the Illumina Global Screening Array platform resulted in 518,653 variants available for imputation. We performed imputation (i) using a reference panel consisting of the UK 1000G phase 3 and HapMap reference to predict non-genotyped variants with minor allele frequency (MAF) > 1%, and an in-house dataset consisting of >6,000 Danish whole-genome sequences to improve the prediction of variations with a MAF down to around 0.01% and (ii) employing software developed at deCODE genetics (Iceland) based on the IMPUTE HMM model.^6^ These procedures resulted in 15,493,405 variants available for association analysis. We measured hepcidin in its 25-amino acids, active isoform using an ELISA kit (DRG® Hepcidin 25 HS ELISA).^7^ We performed association analysis using BOLT-LMM v 2.3.2.^4^

**deCODE (hepcidin).** Between 2000-2019 deCODE collected 40,004 plasma samples through two main projects: the Icelandic Cancer Project (52% of participants; samples collected between 2001-2005) and various genetic programs at deCODE genetics, Reykjavík, Iceland (48%). In the Icelandic Cancer Project, we invited all Icelanders with prevalent and newly diagnosed cancer and their relatives to participate in a comprehensive study of cancer, along with a control population, randomly selected from the National Registry. The samples collected at deCODE genetics were mainly collected through the population-based deCODE Health study, and the rest were collected through various programs at deCODE. All participants who donated samples gave informed consent, and the National Bioethics Committee of Iceland approved the study, which was conducted in agreement with conditions issued by the Data Protection Authority of Iceland (VSN_14-015). Personal identities for the participant’s data and biological samples were encrypted by a third-party system (Identity Protection System), approved and monitored by the Data Protection Authority. We included 35,559 participants in the analysis. Genotyping with the Illumina HumanHap300 and OmniExpress resulted in 619,525 variants available for imputation. We performed imputation using an in-house reference panel consisting of 63,118 WGS Icelanders and employing software developed at deCODE genetics based on the IMPUTE HMM model.^6^ These procedures resulted in 19,315,382 variants available for association analysis. We measured hepcidin using the SomaScan version 4 assay (SomaLogic), which contains 5,284 aptamers providing measurement of relative binding of the plasma sample to each of the aptamers in relative fluorescence units. We performed association analysis using BOLT-LMM.^4^

**FinDonor_1 & FinDonor_2 (sTfR).** FinDonor 10000 (FinDonor) is a prospective study observing blood donor iron stores and genetic and lifestyle factors associated with iron stores.^8^ We obtained ethical approval from the Ethical Board of Helsinki University Hospital, Helsinki. While FinDonor is a single cohort, for practical reasons it was genotyped and imputed in two parts with different methods; these are referred as FinDonor_1 and FinDonor_2 in this paper. In FinDonor_1, we performed genotyping with Illumina HumanCoreExome-24v1-1_A BeadChip and called genotypes with GenomeStudio v. 2011.1. Of 551,839 genotyped variants, we discarded 6471 (1.2%) due to low call rates, bad cluster separation, low signal intensity, quality scores and heterozygote excess. We imputed the remaining variants using the Impute 2.3.2 software^9^ with 1000G Phase 3 reference panel, returning 7,103,553 variants available for association analysis. We measured sTfR using Li-Hep tubes, Tina-quant sTfR assay (Roche Modular) and Tina-quant sTfR assay (Abbott Architect). We performed association analysis using PLINK v1.90b4.5 (<https://www.cog-genomics.org/plink/>). In FinDonor_2, we performed genotyping with FinnGen ThermoFisher Axiom custom array,^10,11^ and called genotypes with GenCall and zCall algorithms for Illumina and AxiomGT1 algorithm for Affymetrix data, yielding 540,008 genotyped variants after filtering out those with low call rates, minor allele count < 3, and heterozygosity excess. We imputed the remaining variants using the Beagle 4.1 software^9^ with Finnish-specific SISu v3 reference panel, returning 11,326,233 variants available for association analysis. We measured sTfR using Li-Hep tubes, Tina-quant sTfR assay (Roche Modular) and Tina-quant sTfR assay (Abbott Architect). We performed association analysis using SAIGE version 0.39.1.^12^

**InCHIANTI (sTfR).** Invecchiare in Chianti (InCHIANTI) is a population-based study of 1453 participants aimed at evaluating the factors that influence mobility in the older population living in the Chianti region of Tuscany, Italy.^13^ The study protocol was approved by the Italian National Institute of Research and Care of Aging Institutional Review and Medstar Research Institute (Baltimore, MD). We extracted DNA from overnight fasted blood samples and used Illumina Infinium HumanHap 550K SNP arrays for genotyping and BeadStudio v.3 for genotype calling.^14^ Imputation of 498,838 variants using MaCH v1.0.18^15^ with 1000G ALL phase1 integrated variant set release v3 (march 2012) returned 19,100,024 variants available for association analysis. We measured sTfR with chemiluminescent immunoassay (Abbott Diagnostics, Abbott Park, Ill, and Nichols Institute Diagnostics, San Clemente, Calif). We carried out association analysis with Mach2qtl (<https://hpc.nih.gov/apps/mach2qtl.html>) in 1097 participants.

**INGI-VB (hepcidin).** The INGI‐Val Borbera (INGI-VB) study comprises 1,785 healthy participants enrolled in the Val Borbera Valley, a geographically isolated region located within the Apennine Mountains in Northwest Italy.^16^ Participants were selected among those having at least one grandfather living in the valley. The laboratory of ASL 22 - Novi Ligure (Alessandria, Italy) performed biochemical measurements on fasted blood samples collected in the morning. The project was approved by the Ethical committee of the San Raffaele Hospital and of the Piemonte Region and all participants signed a consent form.^17^ After genotyping with the Illumina 370K and Illumina OmniExpress 700K platforms, we imputed 648,130 variants using Impute 2.2.2, resulting in 88,637,726 variants available for association analysis. We measured hepcidin using liquid chromatography tandem-mass spectrometry. We performed association analysis using the Gemma 0.98 software.^18^

**INTERVAL (hepcidin and sTfR).** The INTERVAL study is a randomised trial and bioresource of ~45,000 blood donors enrolled between 2012-2014 in the United Kingdom.^19,20^ The INTERVAL study was approved by Cambridge (East) Research Ethics Committee (reference 11/EE/0538). We genotyped DNA extracted from the buffy coat using the Affymetrix Axiom UKBB array at Affymetrix (Santa Clara, California, US).^1^ We excluded samples with sex mismatches, low call rates, duplication, extreme heterozygosity and non-European descent. We carried out imputation 654,966 variants, using a combined 1000 Genomes Phase 3-UK10K imputation panel on the Sanger Imputation Server (<https://imputation.sanger.ac.uk>). We measured hepcidin in its 25-amino acids, active isoform using an ELISA kit (DRG® Hepcidin 25 HS ELISA) and we measured sTfR directly using an immunoturbidimetric assay (Tina-quant sTfR) with a Roche/Hitachi 902 analyser. We removed all data points lying more than 4.5 interquartile range from the median of log-transformed measurements. We rank-based inverse normally transformed raw residuals of multivariable generalised additive models and performed association analyses in BOLT-LMM v. 2.3.^4^

**KORA_F3 (sTfR).** Cooperative Health Research in the Region of Augsburg (KORA; speaker A. Peters) is a research platform of independent population-based health surveys and subsequent follow-up examinations of participants of German nationality resident in the region of Augsburg, southern Germany.^21,22^ All participants gave informed consent and the study has been approved by the local ethics committee (Ethik-Kommission der Bayerische Landesärztekammer). The S3/F3 cohort started in 1994 and comprised 4856 and 4261 participants aged 25 to 74 years. We performed genotyping using Illumina HumanOmniExpress/HumanOmni2.5M, resulting in 588,307 genetic variants. We imputed these with IMPUTE v2.3.0, using 1000G phase 1 as the reference panel, resulting in 12,008,542 variants available for association analysis. We measured sTfR was using a Tina-quant, Photometric immunoassay (Cobas, Roche). We carried out association analysis using SNPTEST v2.

**NBS (hepcidin).** The Nijmegen Biomedical Study Radboudumc (NBS) is a population-based survey conducted by the Department for Health Evidence and the Department of Laboratory Medicine of the Radboud University Medical Centre, Nijmegen, The Netherlands. The study has been described before.^23^ Briefly, in 2002, 22,451 age and sex-stratified randomly selected adult inhabitants of Nijmegen, a city located in the eastern part of the Netherlands, received an invitation to fill out a postal questionnaire (QN) including questions about lifestyle, health status, and medical history, and to donate a blood sample for DNA isolation and biochemical studies. A total of 9350 (43%) persons filled out the QN, of which 6468 (69%) donated blood samples. Blood was sampled between 8 AM and 9 PM; not fasting. The first phase of NBS has been followed by four additional phases across the period 2002-16.^23^ Approval to conduct the NBS was obtained from the Radboud university medical center Institutional Review Board. All participants gave written informed consent for participation in the NBS. For this study, we used the subset of 5111 NBS participants. Serum hepcidin was measured in February 2010 in 2576 genotyped samples with an in house developed and validated competitive enzyme-linked immunosorbent assay as described before.^24,25^ Detection limit: 0.18 nmoles/L (number of samples below detection limit=9).

**PREVEND (hepcidin).** Prevention of REnal and Vascular ENd stage Disease (PREVEND) is a prospective study investigating the natural course of increased levels of urinary albumin excretion and its relation to both renal and cardiovascular disease.^26^ The study includes participants aged 28-75 residing in the city of Groningen, The Netherlands, with a urinary albumin concentration of at least 10 mg/L (N=7768) and a randomly selected control group with a urinary albumin concentration less than 10 mg/L (N=3395). For the current GWAS, we used data from the second survey, which took place between 2001 and 2003, as hepcidin measurements were only available from this time period (N=2817 participants with hepcidin and genetic information). We performed genotyping using the Illumina CytoSNP12 v2 chip, a whole-genome scanning panel that includes up to 220,000 common genetic variants. We excluded samples based on call rates below 0.95, gender mismatch, duplicate discordance and genetic similarity. We performed imputation with SHAPEIT (v2.11) and IMPUTE2 (v2), using 1,000 Genomes haplotypes Phase I integrated variant set release (v3, March 2012) in NCBI build 37 (GRCh37) as the reference panel, which yielded 11,427,983 variants available for association analysis. We measured serum hepcidin concentrations with a competitive enzyme-linked immunosorbent assay (ELISA), as described previously.^25^ We performed the GWAS on the inverse rank log-normalized residuals of hepcidin using an additive genetic model in SNPTEST (v2.4.1),^27^ in 1,965 participants with complete covariate information.

## Data cleaning and quality checks

We established a data-management and quality-check pipeline for study-specific GWAS results that included the following steps: (i) harmonisation of file names and column labels; (ii) removal of duplicated variants, variants with alleles that could not be mapped to dbSNP and variants with invalid values (e.g. negative standard errors); (iii) estimation of study-specific genomic inflation factor lambda which for hepcidin was between 1.001-1.036 and for sTfR was between 0.989-1.040, indicating low genetic inflation; (iv) generation of study-specific Manhattan plots that were broadly consistent with the Manhattan plot based on the meta-analytic estimates. We added rsID to the meta-analysis file using dbSNP v. 154.

## Genome-wide summary statistics

We estimated SNP-based heritability and genomic inflation factor using LDSC v. 1.0.1^28^ with the 1000G EUR reference panel. Briefly, heritability is defined as the proportion of between-individual variation in a phenotypic trait that is explained by common genetic variants in a population.^29^ The inflation factor is the ratio between the median of all observed GWAS test statistics and the median of the expected statistics under the null hypothesis.^30^ In a population without sources of genetic bias (such as population stratification, cryptic relatedness and genotyping errors), it is expected that only a few variants have large test statistics indicating strong association with the trait of interest. In that ideal situation, the inflation factor would be 1 or very close to 1. Using LDSC, we also performed LD score regression to assess whether the distribution of test statistics was due to polygenicity or sources of bias such as cryptic relatedness and population stratification. This approach assesses if variants that tag more of the genome (i.e. have high LD score) have a greater opportunity to tag causal variants and therefore have higher test statistics on average than variants that have low LD score.

## Variant annotation and effect prediction

We used Ensembl Variant Effect Prediction (VEP) to obtain information for several measures of functional consequence for each sentinel variant and their proxy variants. VEP places each variant into one of four impact categories: (i) high-impact variants, predicted to ablate or truncate transcripts, alter the reading frame or alter splicing; (ii) moderate-impact variants, predicted to alter protein sequence through addition, loss or change in amino acids, or to result in loss of regulatory regions; (iii) low-impact variants that are unlikely to alter function (synonymous changes, start/stop retained), and (iv) modifier variants that may alter expression levels without substantially changing the protein product. First, for each sentinel variant we selected all proxy variants with r^2^>0.7 using linkage disequilibrium information from the INTERVAL study.^1^ Second, we performed VEP for each proxy. Third, for each sentinel variant we identified the proxy variant(s) most likely to affect the gene product (e.g. the most correlated missense variant or the most correlated variant with a high or moderate impact on the gene product). If no such variant(s) could be identified, we selected the sentinel variant itself. We present all VEP results in tabular format (**Supplementary Table 5**) with the option to show results only for the proxy variant(s) identified as most likely to impact the gene product for each sentinel variant.

## Phenome scans

We conducted phenome scans drawing on the curated database of >65 billion genetic summary statistics available in Phenoscanner v.2.^31^ For each independent variant and its r^2^≥0.8 proxy, we retrieved all genome-wide significant (*P* < 5×10^-8^) associations with phenotypic traits, expression quantitative trait loci (eQTL), methylation quantitative trait loci (methQTL), metabolite quantitative trait loci (mQTL) and protein quantitative trait loci (pQTL). We collapsed and tabulated results so that only the association with the lead variant or its most correlated proxy was presented for each trait. For each trait, we displayed information using the following format: Phenotype −log 10 of p-value, chromosome:position (GRCh37) and PMID of relevant article.

## Colocalization and candidate gene mapping

To define the genomic regions for colocalization, we first mapped the above-defined conditionally independent and uncorrelated GWAS signals to their nearest gene and then collapsed overlapping genes within 200 Kb from each other. The positional window was based on previous research in eight populations establishing that only a few *cis*-eQTLs were extending beyond +/- 200 Kb from the transcription start site.^32^ The process was performed independently for hepcidin- and sTfR-associated variants. This led to the definition of 43 non-overlapping loci. Of these, 21 already had a biologically plausible candidate gene (e.g. *HFE*, *TMPRSS6*, *HAMP*, *TFRC*; identified by the label ‘Biology’ in column E of **Supplementary Table 8**).

For the remaining 22 loci that did not have an obvious candidate gene, we extracted all meta-analytic GWAS associations with the relevant iron trait (either hepcidin or sTfR). To ensure that highly correlated variants had consistent genetic associations, we restricted our panel of regional GWAS associations to variants estimated from at least 80% of the maximum meta-analytic sample. For each locus, we also extracted (i) expression quantitative trait loci (eQTL) data across 49 tissues for genes that had at least one significant and independent association in the GTEx Consortium v.8 study^33^ (ii) protein quantitative trait loci (pQTL) data for all aptamers measured in INTERVAL that had at least one genome-wide association in Sun et al.’s study^34^ (N=1927), to enable colocalization for variants that act through regulation at the protein level rather than via gene expression levels (e.g. missense variants), and (iii) an LD matrix from the 1000G study (phase 3 release).^35^ For each locus, we retained only variants available in all datasets (hepcidin or sTfR GWAS, eQTL, pQTL and LD). We allowed for more than one signal to be identified in each region across each tissue/gene, using Sum of Single Effects (SuSiE) v. 0.11.92 and Coloc v. 5.1.0 with linkage disequilibrium information from 1000G.^36,37^ Coloc uses the SuSiE framework to simultaneously estimate credible sets of variants and therefore to estimate the distinct signals in a genomic region while accounting for the correlation between variants. Coloc then computes an approximate Bayes factor with both GWAS and eQTL summary statistics to estimate the posterior probabilities (PP) of each signal. Whenever the SuSiE algorithm was unable to complete successfully, we assumed a single colocalizing variant in each genomic region. In either case (multiple-variant or single-variant colocalization), Coloc leads to five scenarios for each combination of lead variant_GWAS_ vs variant_QTL_: PP0, PP1, PP2, PP3 and PP4, with PP3 indicating the probability of both traits being associated with different causal variants and PP4 indicating the probability of both traits being associated with a shared causal variant. We defined strong evidence of colocalization as PP3 + PP4 ≥ 0.99 and PP4/PP3 ≥ 5 as described previously.^38,39^ For 12/22 loci in which more than one colocalizing gene was available, we assigned the most likely causal gene based on the gene’s biological function (‘Colocalization and potential biology’ in column E of **Supplementary Table 8**). For 5/22 loci in which it was not possible to identify a biologically-plausible candidate gene, we included all colocalizing genes as candidate genes (‘Colocalization’ in column E of **Supplementary Table 8**). For the 5/22 loci lacking strong colocalizing signals, we assigned the nearest gene as the candidate gene (‘Nearest gene’ in column E of **Supplementary Table 8**).

## Assessing potential collider bias in MR instruments

Because some of the health and lifestyle covariates included in our GWAS analysis plan (body mass index, alcohol consumption, smoking, ABO blood group, iron supplements, medications that may affect iron homeostasis, number of whole blood donations) may potentially lead to collider bias^40,41^ and it would have been impracticable to re-run the GWASs in all the cohorts contributing to this study, we compared the betas estimated using the full model specified in our analysis plan with a model that excluded health and lifestyle covariates while retaining all the other covariates (**Supplementary Figure 8**). Both analyses were conducted in up to N=40,197 participants which contributes to the GWASs presented in this and a previous study.^42^ This analysis showed very high correlation between the betas estimated with these two models (r^2^≈1). All betas had the same sign in the two models, apart from two variants (rs10804630 and rs79694859, that we removed from our list of MR instruments.

## Variance explained by MR instruments

For both locus-based and polygenic instruments, we calculated the variance explained in up to 40,228 INTERVAL participants by subtracting the variance explained by a covariate-only linear regression model (including the following parameters: age, sex, first 10 principal components) from the variance explained by the full model (including the above covariates and instrument-specific genetic variants). We estimated both models in the same individuals. For each locus-based instrument, we fitted the covariate-only and full models using the trait most strongly associated with the locus sentinel variants (**Supplementary Table 10**). For the polygenic instrument, we performed these analyses using transferrin saturation.

## Power calculations

We conducted power calculations of MR analysis assuming a 5% significance level and a 4.4% variance explained, corresponding to the variance explained by the polygenic MR instrument. We performed these analysis using the ‘results_binary’ function available in the ‘mRnd’ R script.^43^ We found ≥90% power to detect an OR of 1.5 for outcomes with a case count equal to or greater than the median (N≥38,974) (**Supplementary Figure 6**).

## Colocalization analysis after locus-based MR

To remove locus-based MR associations driven by genetic confounding, we performed colocalization analysis for all associations below the Bonferroni-corrected p-value threshold, using the following procedure. Firstly, we selected all the genetic variants available in UKBB (with INFO≥0.8 and MAF≥1) and the meta-analysis of the most strongly associated iron trait in the locus-specific genomic region with +/- 200 Kb flanking (**Supplementary Table 10**). Secondly, for all genetic variants included in the region, (i) we extracted summary statistics from the relevant iron trait meta-analysis, (ii) we estimated genetic associations with MR-associated diseases and traits, while adjusting for age, sex (for non-sex-specific outcomes) and the first 10 principal components; (iii) we extracted a locus-specific linkage disequilibrium matrix from UKBB. Thirdly, we performed multiple-variant (SuSiE) or, if this failed, single-variant colocalization analysis as described above in the ‘Colocalization and candidate gene mapping’ section of the **Supplementary Information**. We defined as suggestive evidence all signals with PP3 + PP4 ≥ 0.90 and PP4/PP3 ≥ 3, as described previously.^38,39^

# Supplementary Results

## Genome-wide statistics, variance explained and genetic/phenotypic correlation

Genomic inflation factors (λ) were 1.08 for sTfR and 1.06 for hepcidin (**Supplementary Figure 9**), indicating little inflation of test statistics. LD score intercepts were low for both traits, suggesting that most of the inflation was due to polygenicity rather than population structure.

SNP-based heritability estimates were 4.1% for hepcidin and 16.5% for sTfR, suggesting greater susceptibility of hepcidin to environmental stimuli compared to sTfR, and genetic associations were typically stronger for sTfR compared to hepcidin despite the smaller sample size (**Figure 2A**).

Genetic and phenotypic correlations between hepcidin, sTfR and other iron traits (serum iron, ferritin, transferrin saturation, TIBC) were broadly concordant (**Figure 2B**, **Supplementary Table 2**). One exception was the correlation between sTfR and TIBC – the phenotypic correlation was 0.60 (±0.004) whereas the genetic correlation was 0.05 (±0.065), which indicates that non-genetic factors may be strongly influencing the relationship between the two traits.

## Genetic associations of the 52 lead variants with iron traits

Genetic associations of this study’s 52 lead variants with iron traits (serum iron, ferritin, TSAT, TIBC, hepcidin, sTfR) are presented graphically in **Supplementary Figure 10**. The plot shows that variants in loci coding for hepcidin (*HAMP*) and transferrin receptor (*TFRC*, *TFR2*) have stronger genetic associations with hepcidin and sTfR, respectively, compared to other iron traits. This appears in keeping with the direct biological role of these loci. Similar remarks can be made for *PCSK7* (Proprotein Convertase Subtilisin/Kexin Type 7), involved in sTfR shedding. By contrast, variants in iron sensing *HFE* seem to have stronger associations with transferrin saturation (TSAT) and TIBC compared to hepcidin and sTfR. This suggests that the effects of these variants on hepcidin and sTfR may be indirect, downstream to their effects on traits reflecting circulating iron levels.

## Sensitivity GWASs adjusted for age and sex

We performed sensitivity GWASs adjusted only for age, sex and the first 10 principal components of ancestry in INTERVAL, which is the largest study contributing to our meta-analysis for both hepcidin (N=37,705; 40% of the meta-analytic sample size of the main analysis) and sTfR (N=40,091; 88%). As shown in **Supplementary Figure 11**, Miami plots comparing associations estimated with the original model included in the paper (**Supplementary Information,** **page 2**) (top half of the plot) with associations estimated with the model adjusted for age, sex and the first 10 principal components (bottom half of the plot), show no visual impression of major differences between the two analyses. Additionally, we noted a genetic correlation, estimated with LDSC v. 1.0.1^28^ using the 1000G reference panel, of ≈1.00 for both hepcidin and sTfR.

## Sensitivity GWASs adjusted for C-reactive protein

We performed sensitivity GWASs adjusted for C-reactive protein, in addition to the covariates included in the main model (**Supplementary Information,** **page 2**) in three studies with hepcidin and available C-reactive protein data (PREVEND, deCODE, INTERVAL: N=74,855; 82% of the meta-analytic sample size of the main analysis) and in two studies with sTfR and C-reactive protein data (InCHIANTI and INTERVAL: N=41,188; 91%). The results are presented in **Supplementary Figure 12**. From the Miami plots, there was no visual impression of substantial differences between the genetic associations generated from the statistical model included in our initial submission and the model additionally adjusted for C-reactive protein. We noted a genetic correlation, estimated with LDSC v. 1.0.1^28^ using the 1000G reference panel, of ≈0.93 for hepcidin and ≈0.98 for sTfR, suggesting little to no substantial genome-wide differences between the estimates of the two models. Detailed comparisons for the 52 main sentinel variants presented in the paper show no substantial differences between the two models (**Supplementary Figure 12**). The maximum absolute difference was 0.085 for rs104894696 among the 20 hepcidin-associated sentinel variants and 0.016 for rs200307986 and rs187669805 among the 32 sTfR-associated sentinel variants, suggesting that inflammation is unlikely to influence the main genetic associations reported in the present study.

# Supplementary Figures

## **Supplementary Figure 1**. Putative causal effects of iron-related loci on disease outcomes

MR associations of 33 loci with 292 disease outcomes in up to 1,492,737 deCODE, FinnGen, MVP and UK Biobank participants, before (**A**) and after (**B**) colocalization analysis.

| **A** Locus-based MR associations before colocalization | **B** Locus-based MR associations after colocalization |
| --- | --- |
| **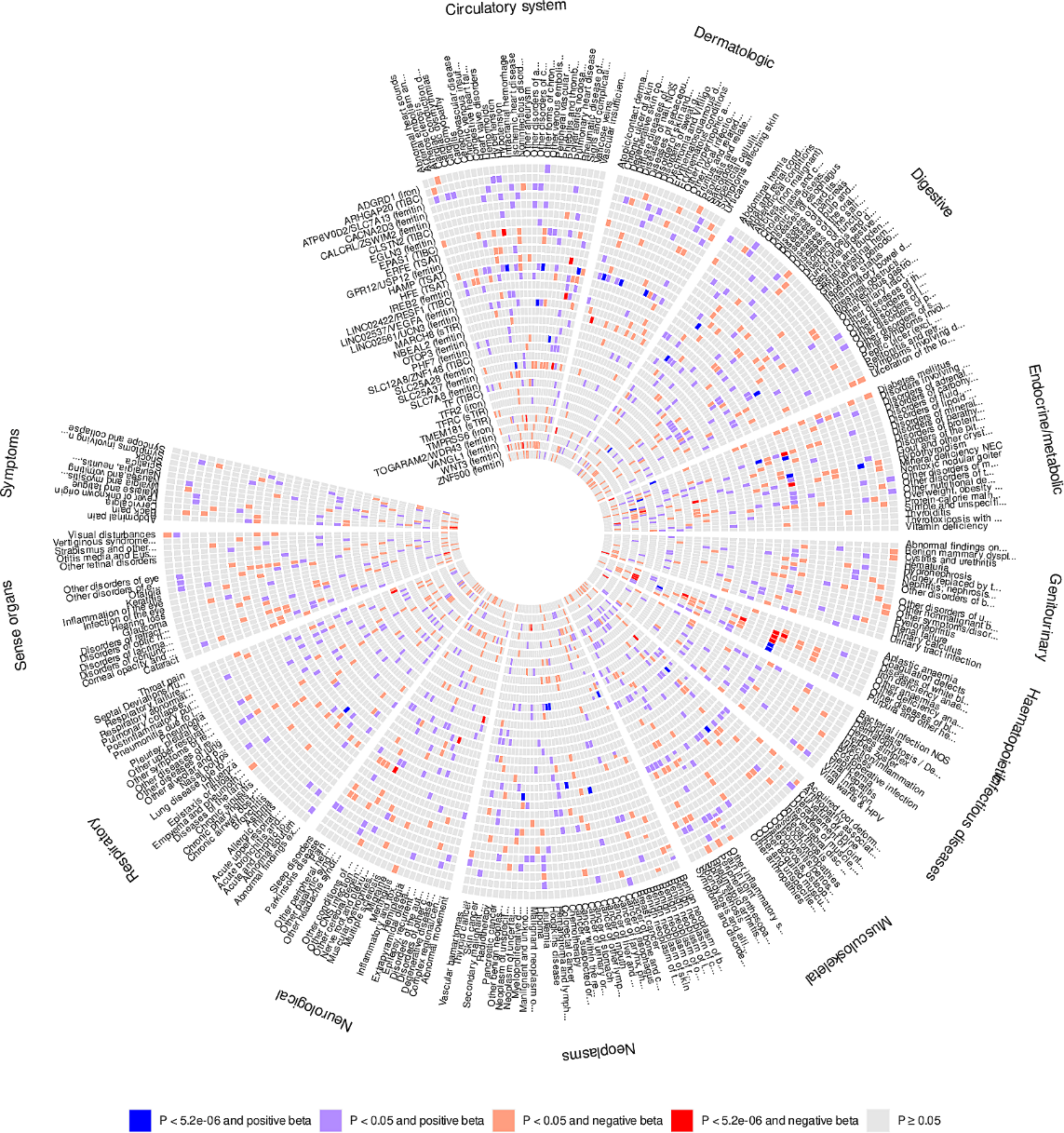** | **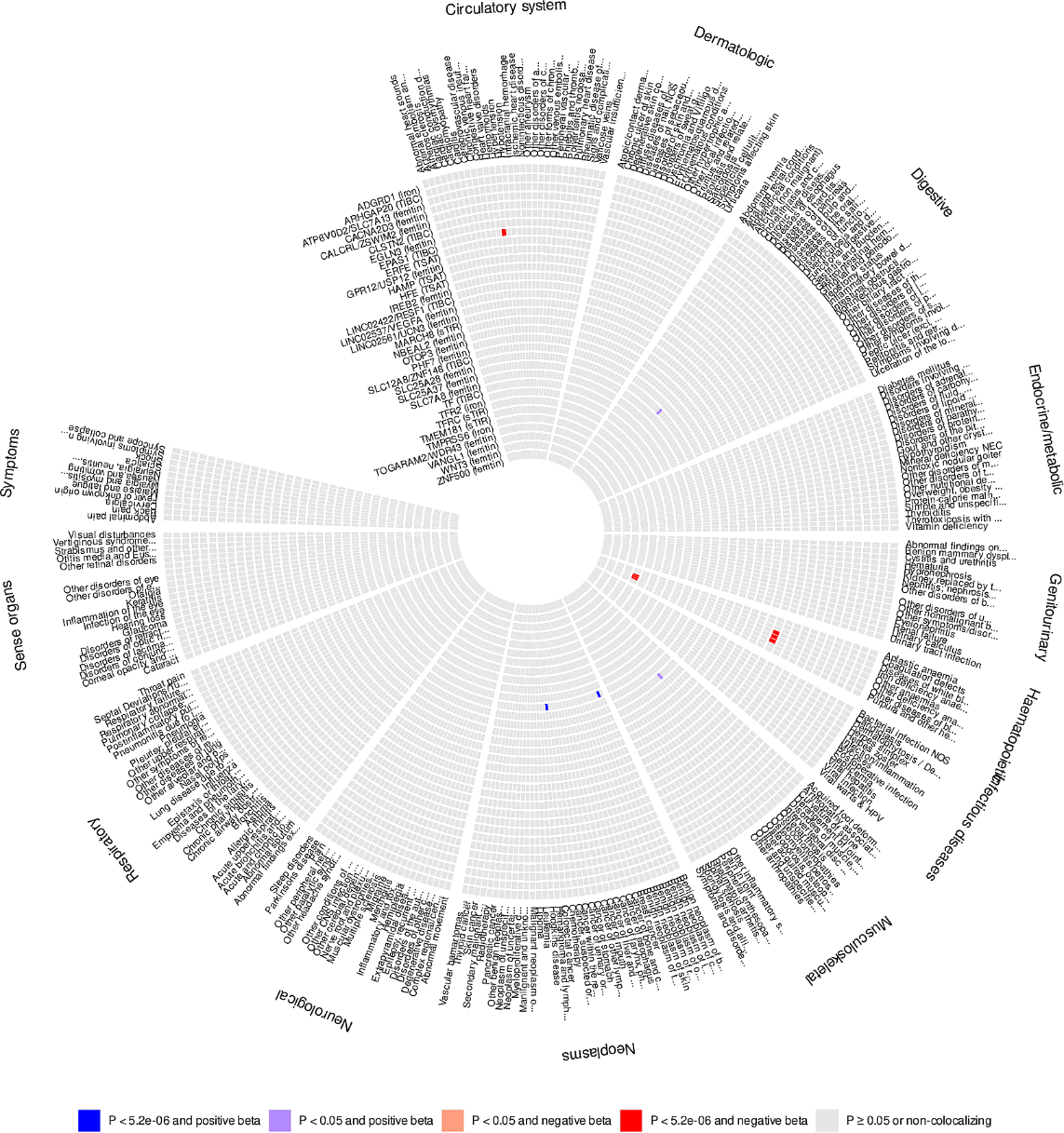** |

## **Supplementary Figure 2**. Putative causal effects of iron-related loci on biomedical traits

MR associations of 33 loci with 47 biomedical traits in up to 860,060 MVP and UK Biobank participants, before (**A**) and after (**B**) colocalization analysis.

| **A** Locus-based MR associations before colocalization | **B** Locus-based MR associations after colocalization |
| --- | --- |
| **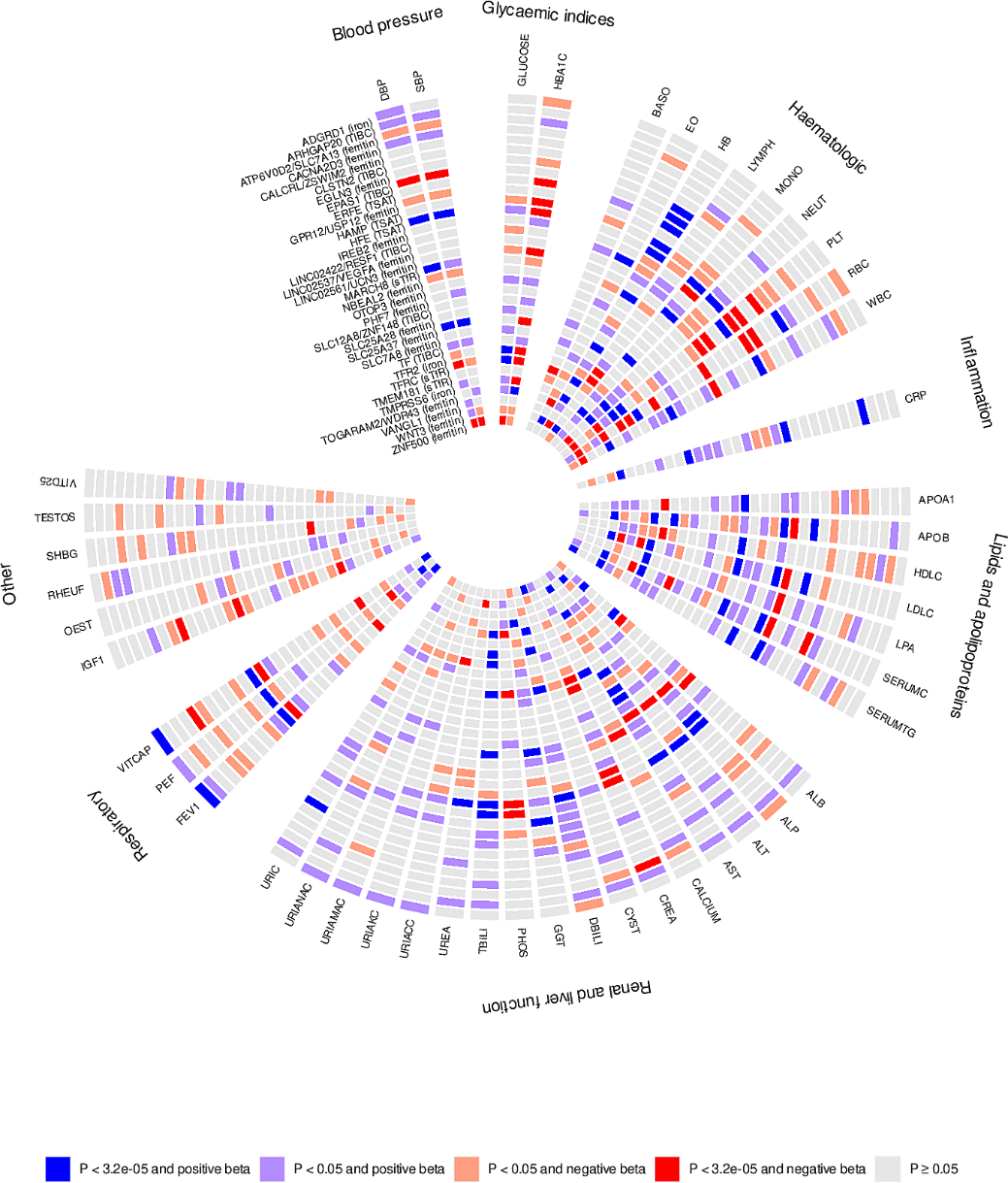** | **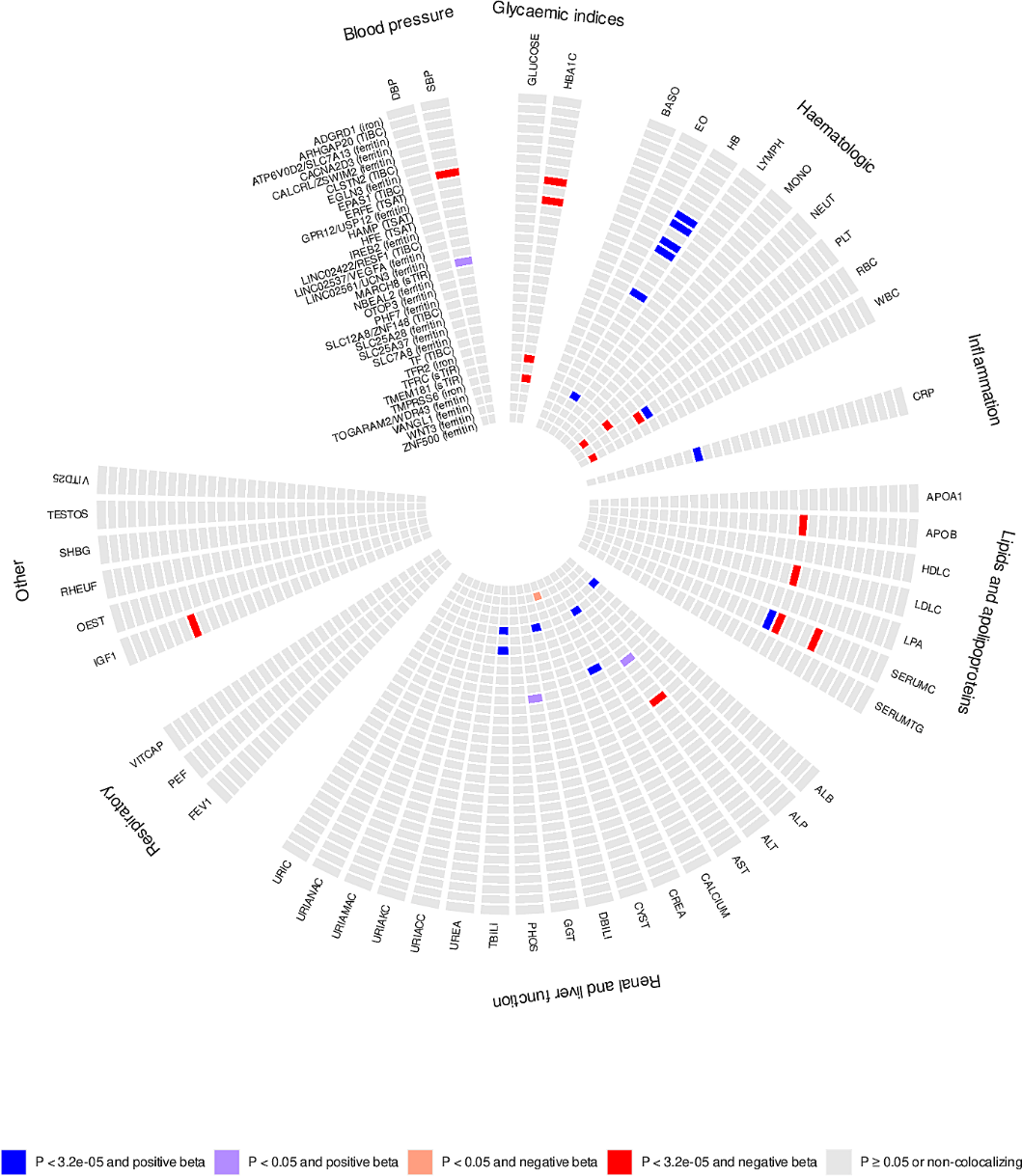** |

## **Supplementary Figure 3**. Polygenic MR of disease outcomes, sensitivity analyses

| ■ *P*<1.7e-04 and positive beta | ■ *P*<0.05 and positive beta | ■ *P*<0.05 and negative beta | ■ *P*<1.7e-04 and negative beta |
| --- | --- | --- | --- |

Diseases ordered and coloured as in Figure 5A:


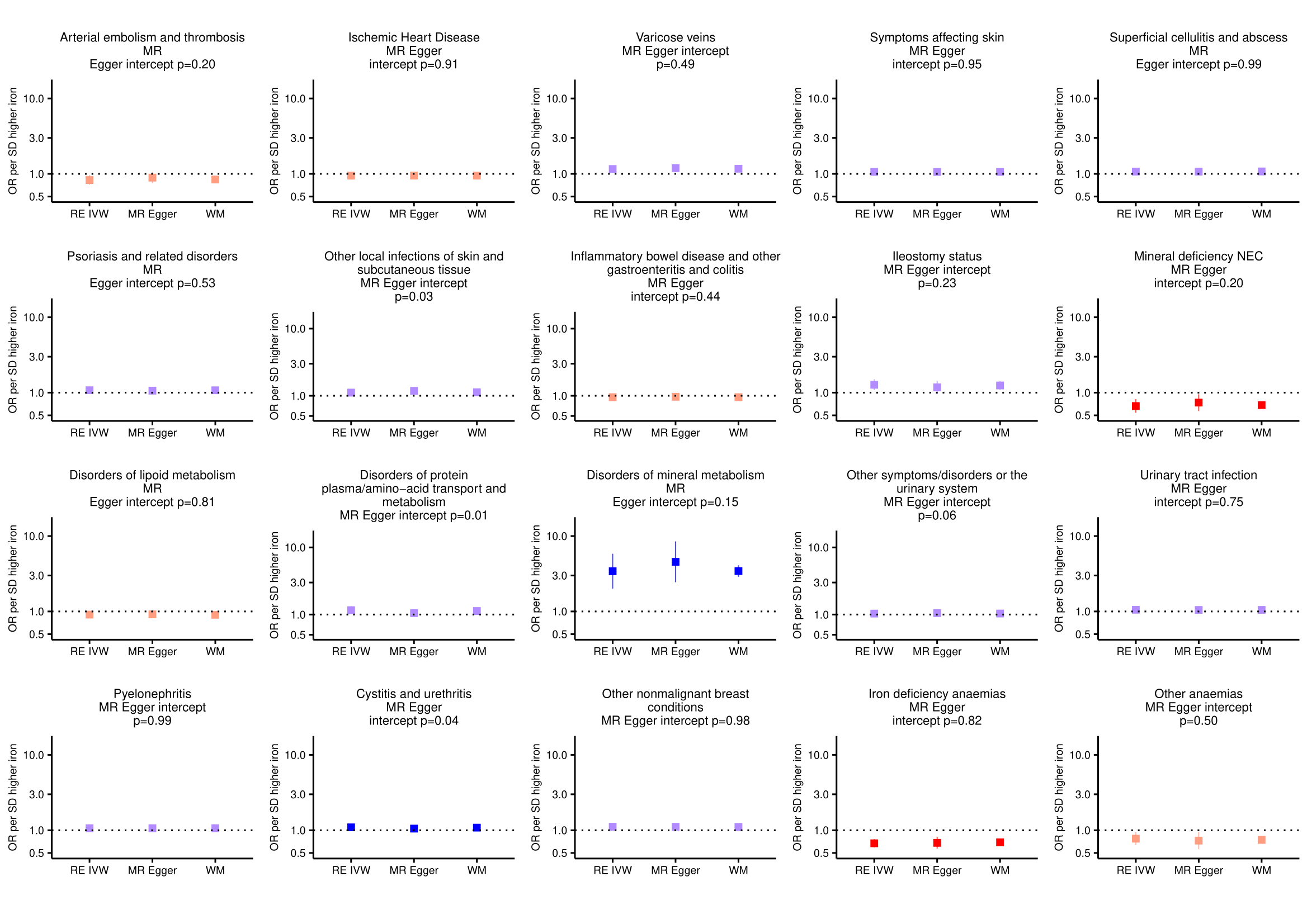


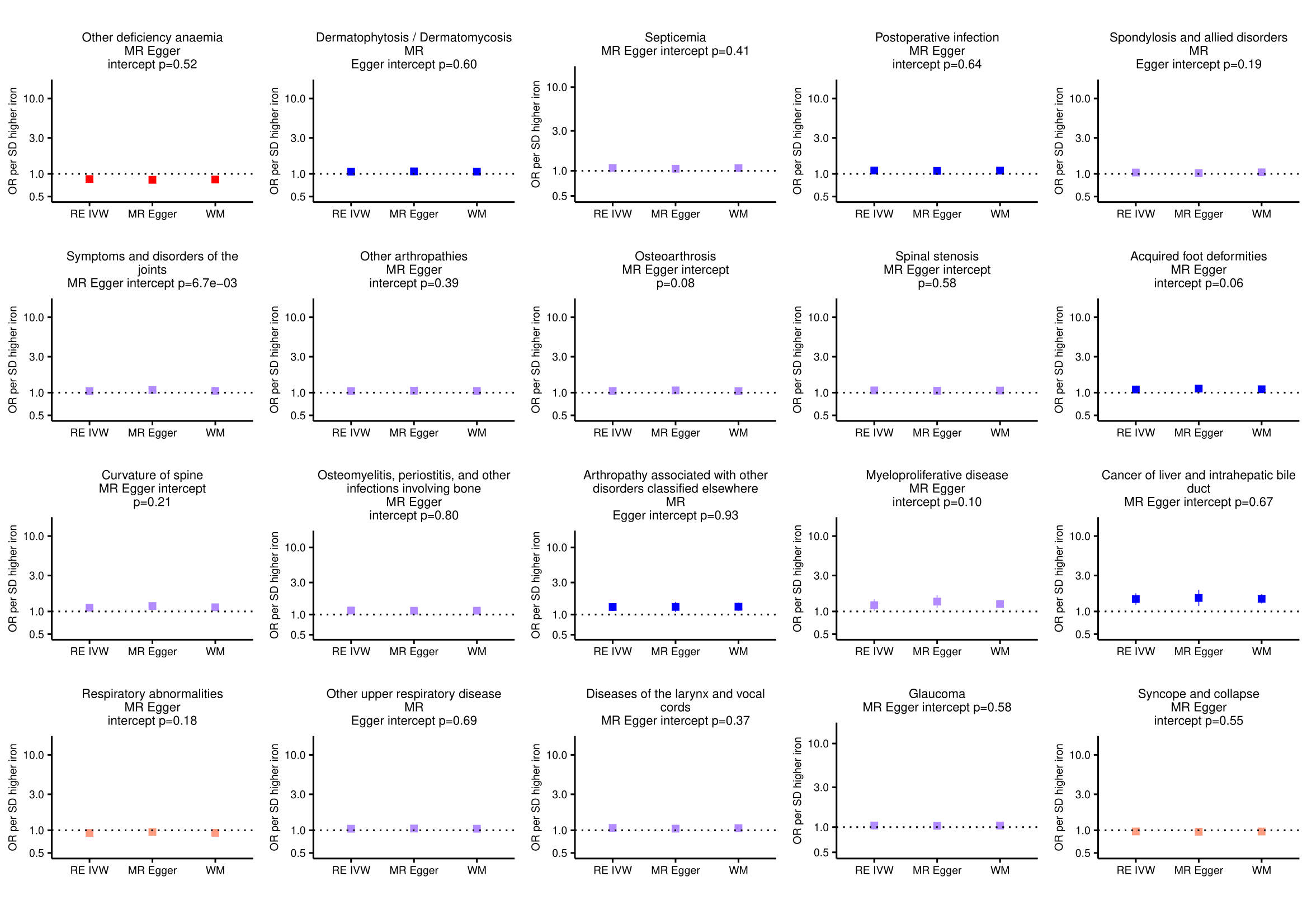


## **Supplementary Figure 4**. Polygenic MR of biomedical traits, sensitivity analyses

| ■ *P*<1.1e-03 and positive beta | ■ *P*<0.05 and positive beta | ■ *P*<0.05 and negative beta | ■ *P*<1.1e-03 and negative beta |
| --- | --- | --- | --- |

Traits ordered and coloured as in Figure 5B:


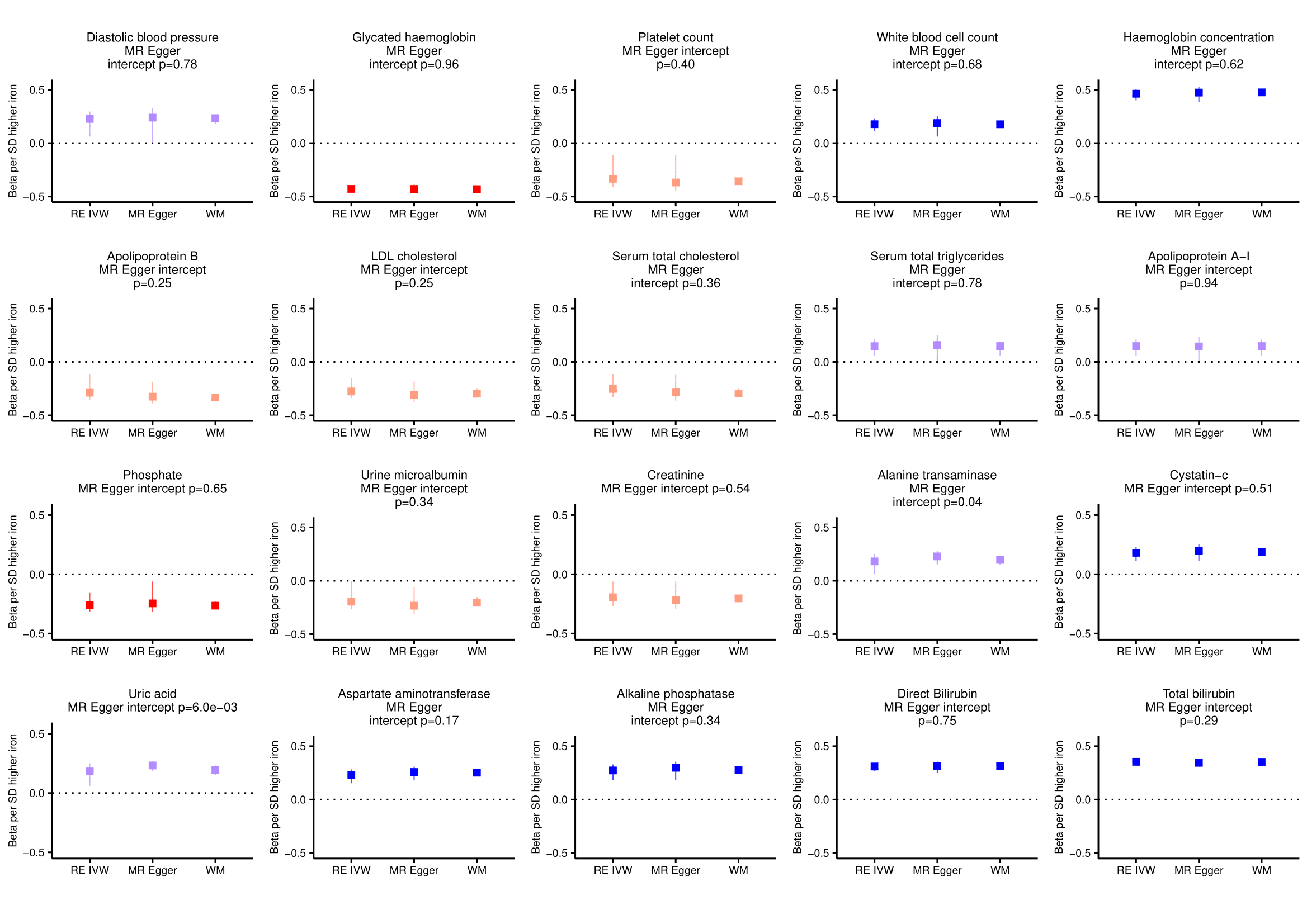


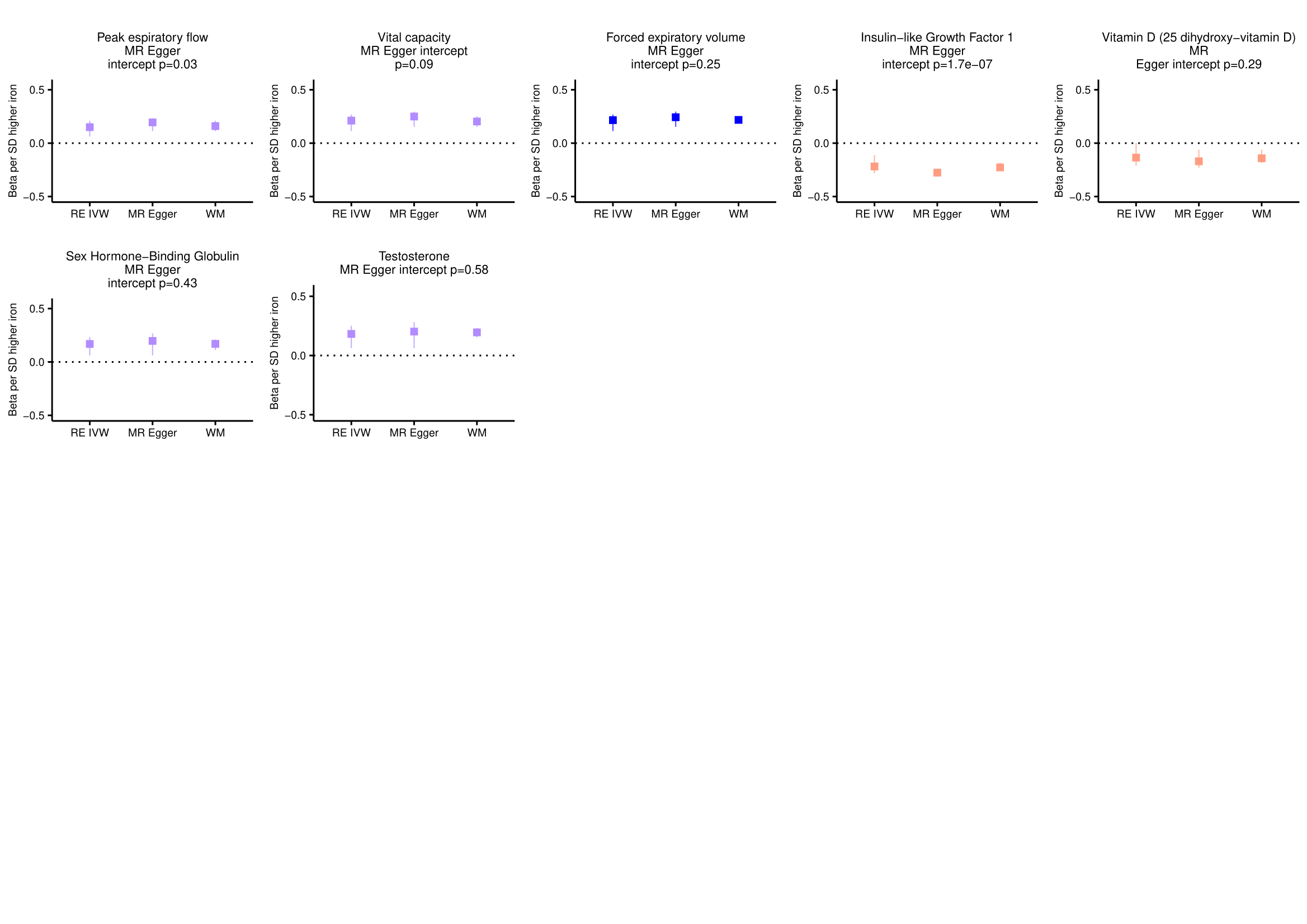


## **Supplementary Figure 5**. Generation of polygenic MR instrument


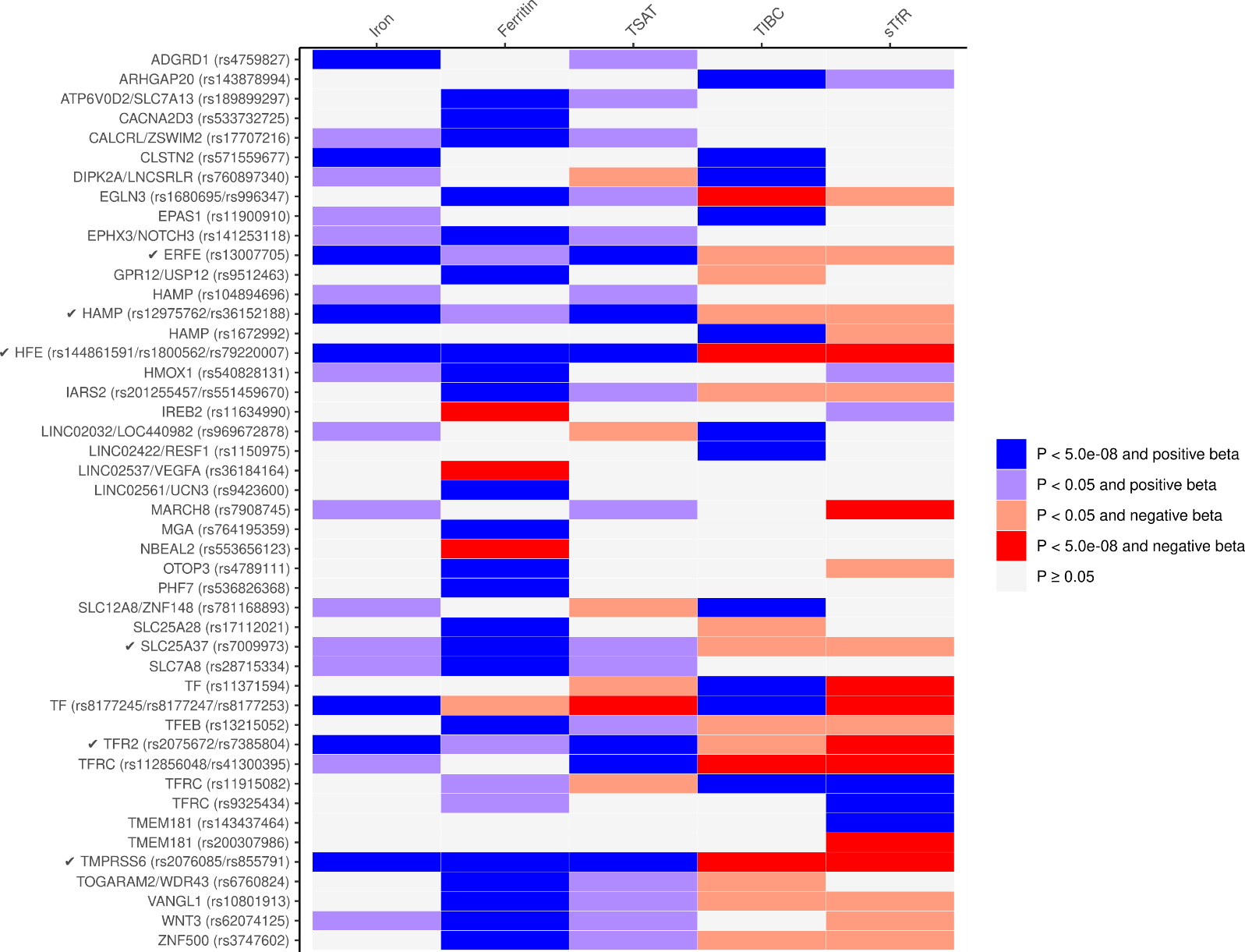


This figure summarises genetic associations across key and common iron-related traits. Genetic variants were included in the polygenic MR instrument of systemic iron status if they were: (i) associated (*P*<5×10^-8^) with at least one iron trait, (ii) nominally associated (*P*<0.05) with all the other iron traits considered; and (iii) with a direction consistent across all traits (e.g. positive for iron, ferritin, TSAT and negative for TIBC and sTfR; or the other way round). Variants selected for polygenic MR instruments are denoted with a ✓ mark. Because hepcidin is influenced by systemic iron status,^44^ associations with hepcidin levels have not been considered in the definition of the polygenic instrument of iron status.

## **Supplementary Figure 6**. Power estimation for the polygenic MR instrument


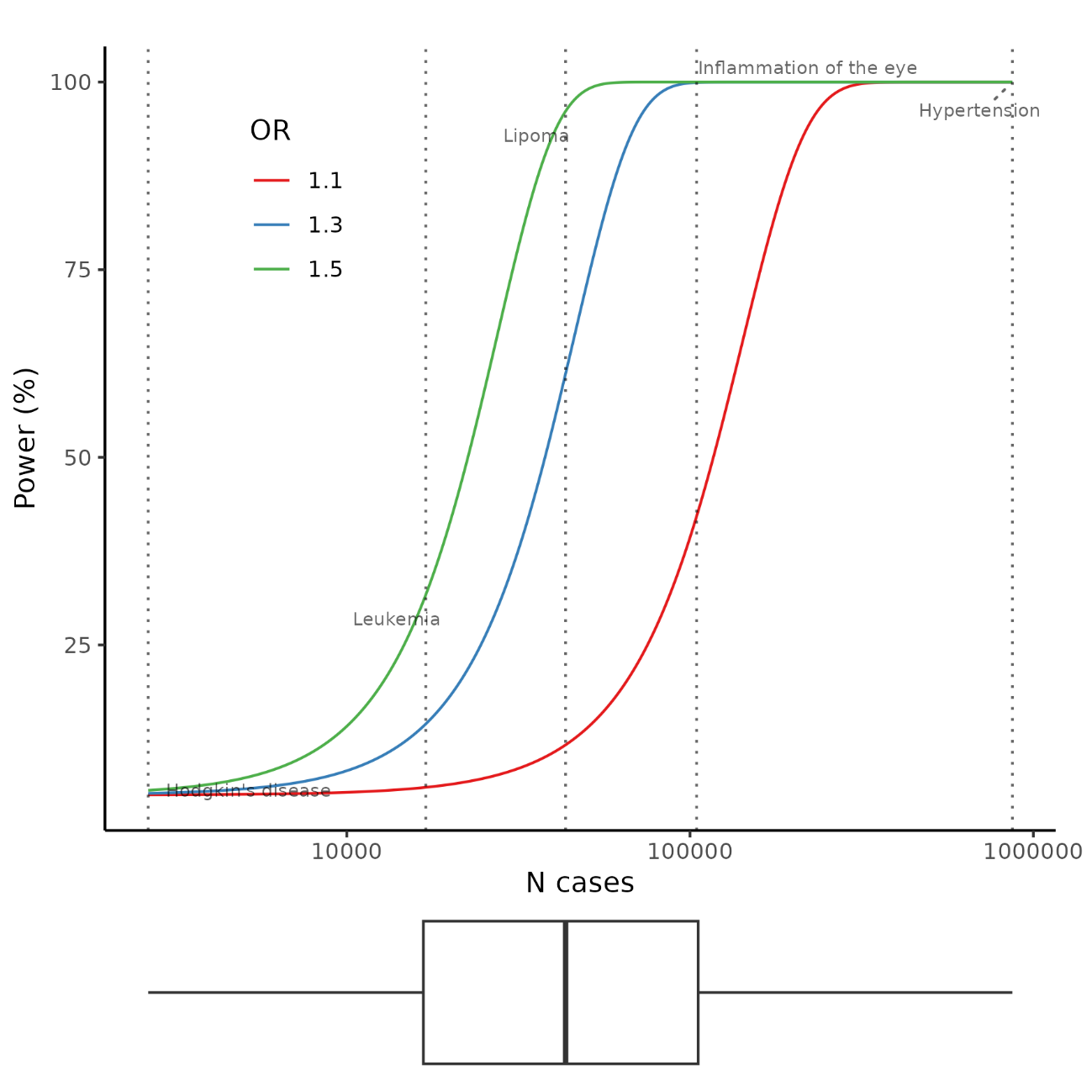


Annotated diseases reflect the distribution of case counts (shown in the above box plot): minimum number of cases (N=2644, Hodgkin's disease), bottom quartile (N=16989, leukemia), median (N=43398, lipoma), upper quartile (N=104538, inflammation of the eye), and maximum (N=868970, hypertension). The line chart and the box plot share the same x-axis. OR, odds ratio.

## **Supplementary Figure 7**. Association of locus-based scores with iron traits


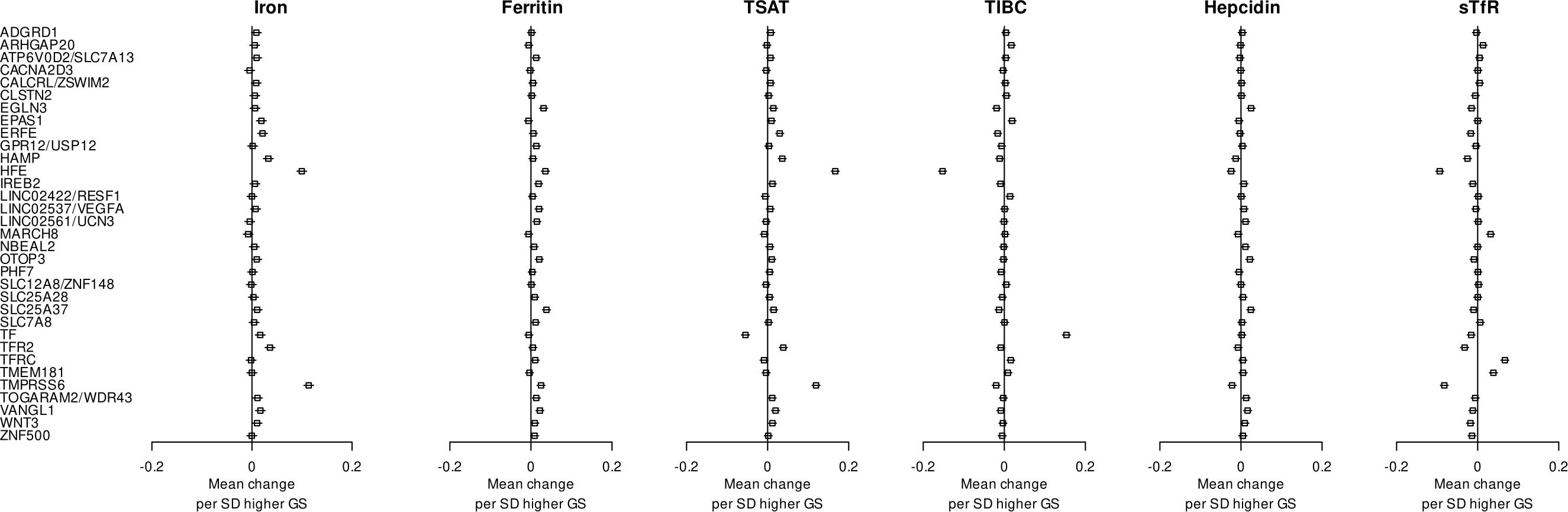


## **Supplementary Figure 8**. Comparison of betas to investigate potential collider bias


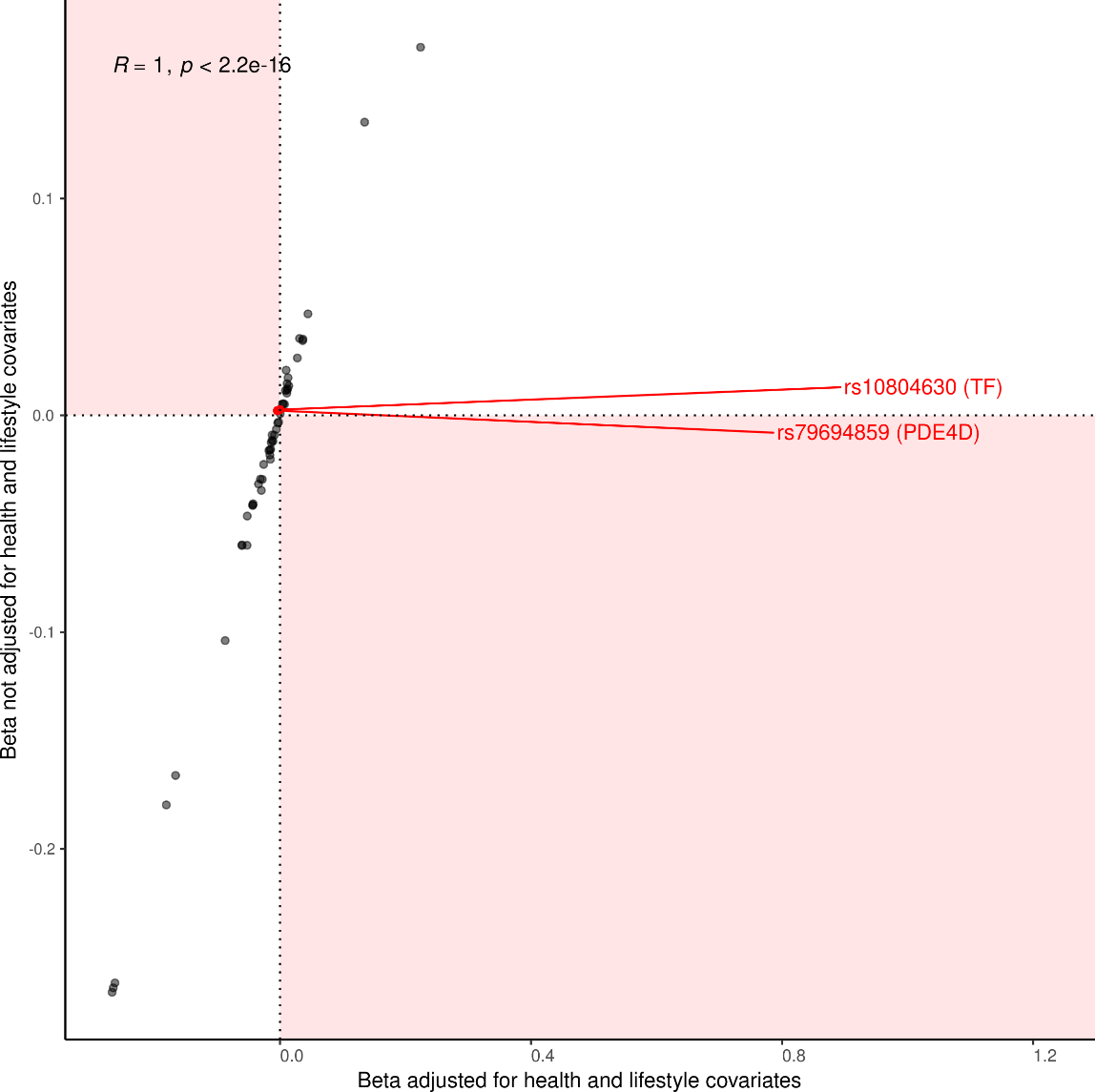


Betas of genetic associations are obtained from the INTERVAL study (up to N=40,197).

## **Supplementary Figure 9**. Quantile-quantile plots and key genomic statistics for hepcidin and sTfR

| **A.** Quantile-quantile plots for hepcidin  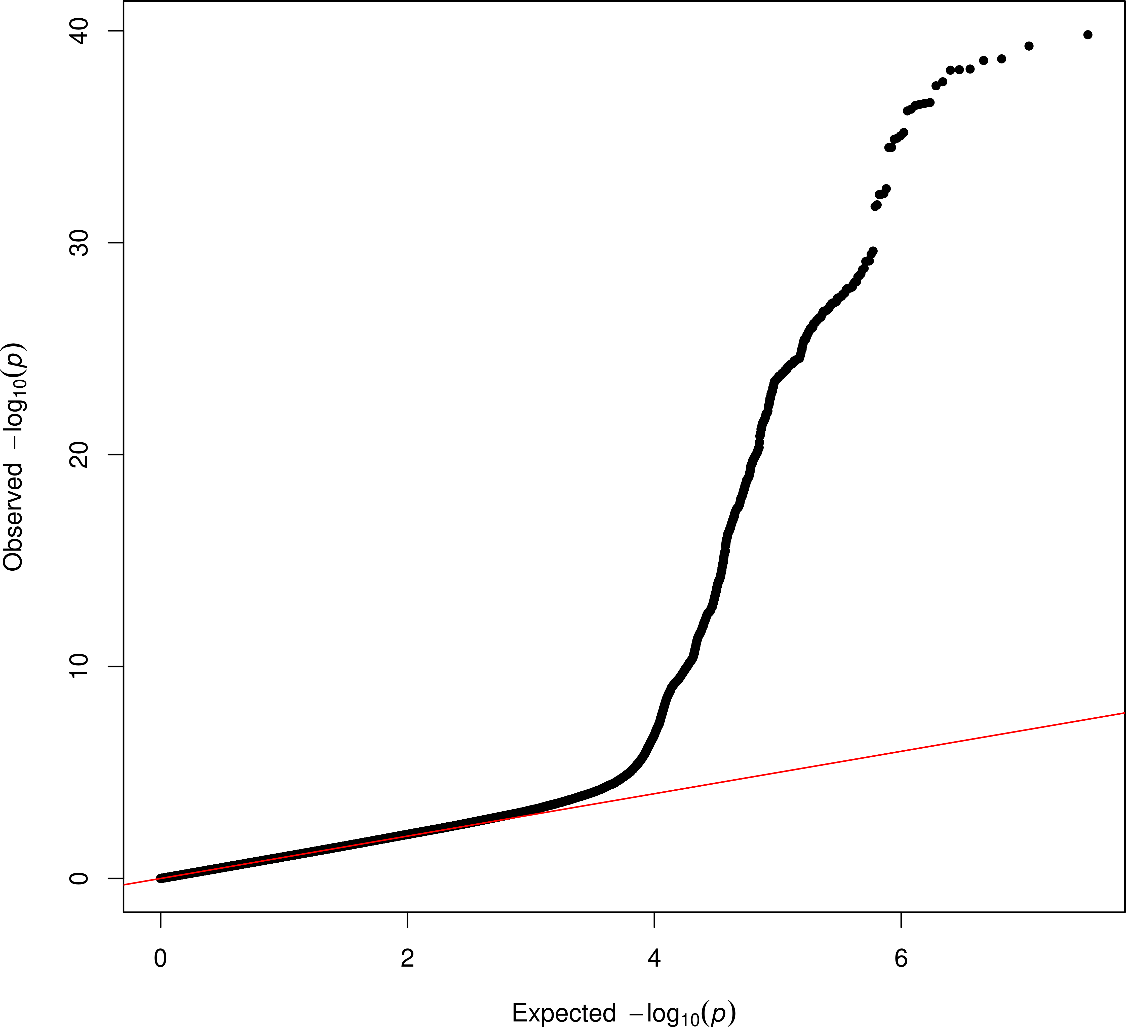 | **B.** Quantile-quantile plots for sTfR  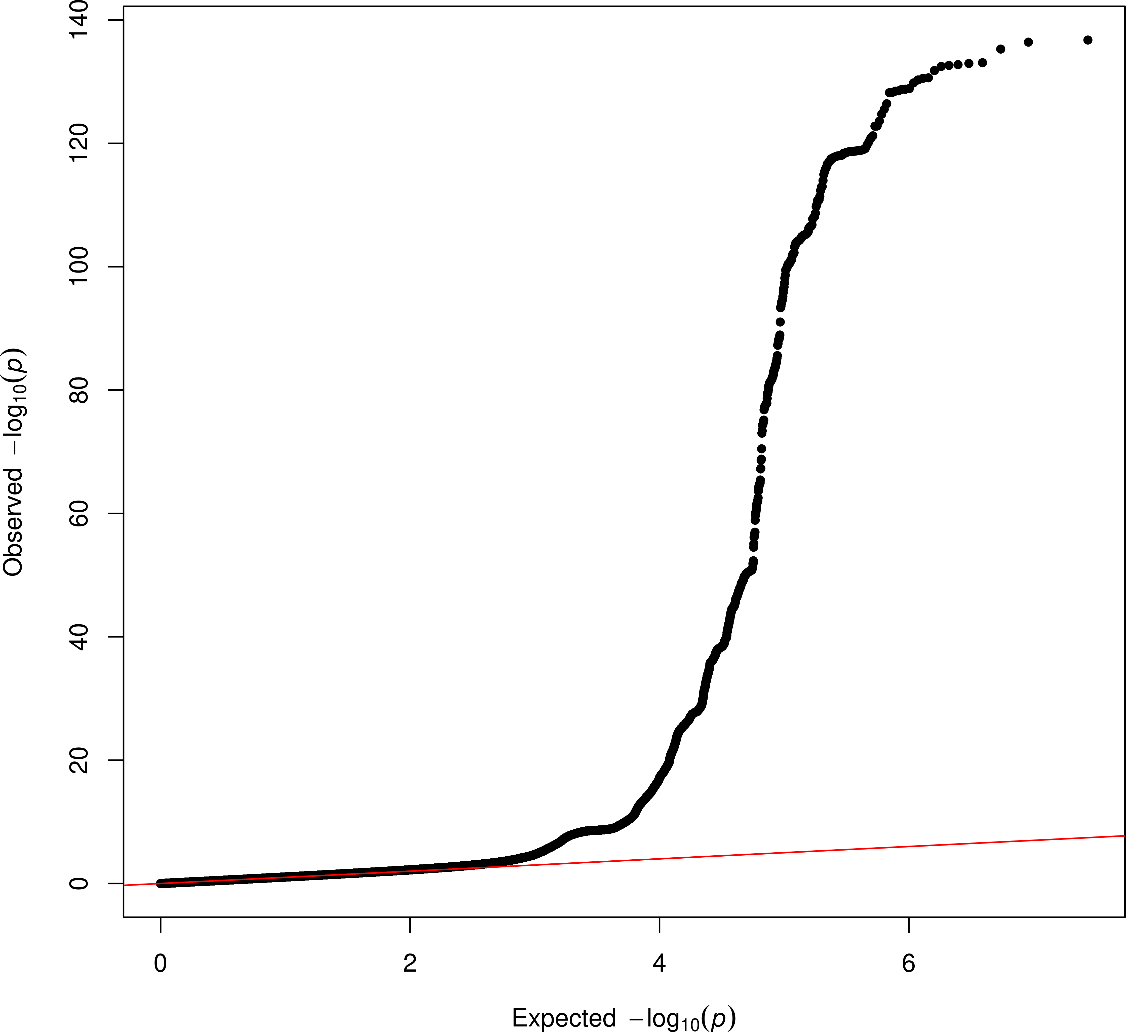 |
| --- | --- |

**C** Key genomic statistics

| **Trait** | **H^2^ (SE)** | **λ** | **Mean χ^2^** | **Intercept (SE)** |
| --- | --- | --- | --- | --- |
| Hepcidin | 0.0411 (0.0072) | 1.0557 | 1.0792 | 1.0030 (0.007) |
| sTfR | 0.1647 (0.0332) | 1.0833 | 1.1685 | 1.0183 (0.008) |

H^2^, heritability. SE, standard error. λ, genomic control. Intercept of linkage disequilibrium score regression

## **Supplementary Figure 10**. Heatmap presenting genetic associations of hepcidin- and sTfR associated variants with iron traits


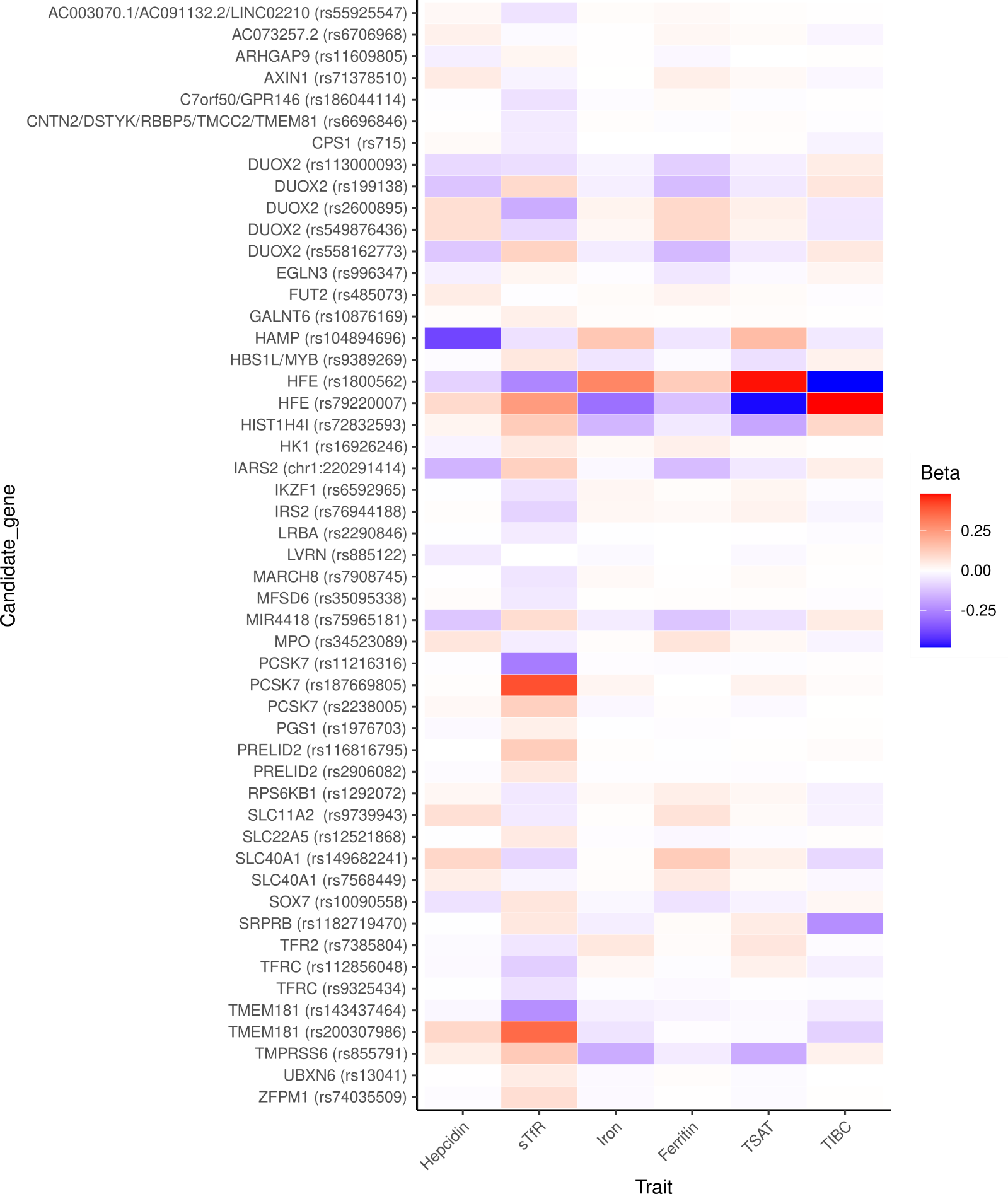


Betas of genetic associations with hepcidin and sTfR are obtained from the present study. Betas of genetic associations with the other iron traits are obtained from Moksnes et al. 2022.

## **Supplementary Figure 11**. Sensitivity analyses adjusted for age and sex

**A.** Hepcidin: Comparison of genetic estimates adjusted for age and sex vs those on the original model in INTERVAL (N=37,705). Miami plot (left) and scatter plot with genetic correlation (right)


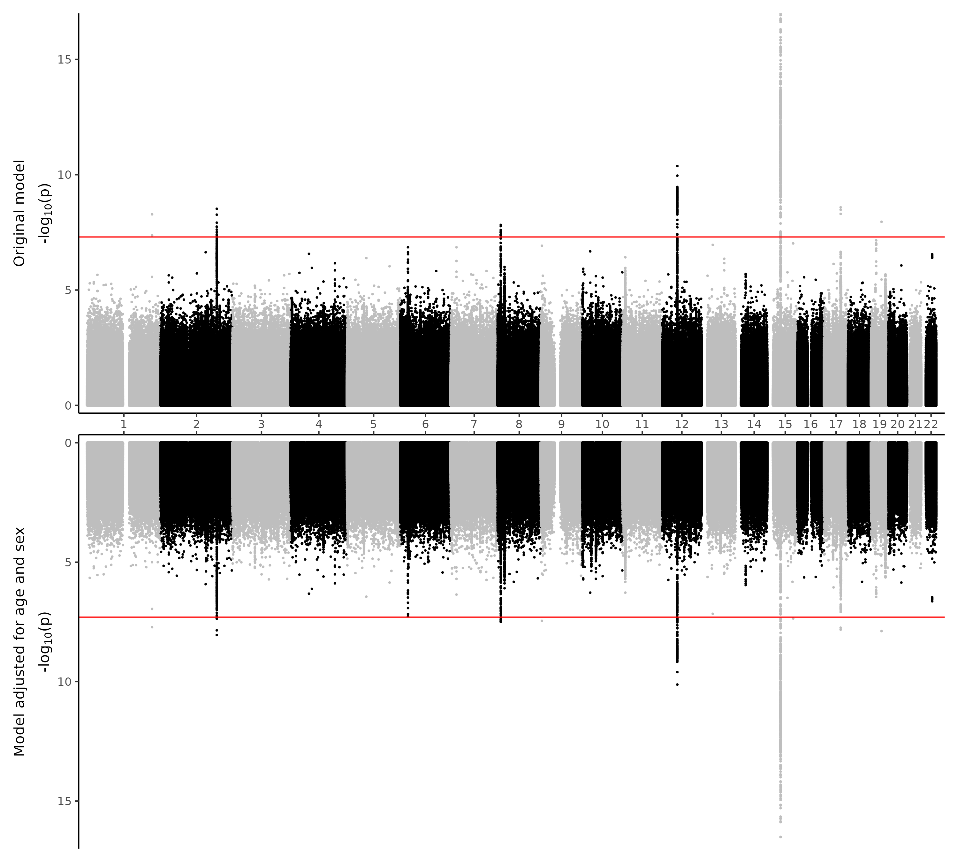


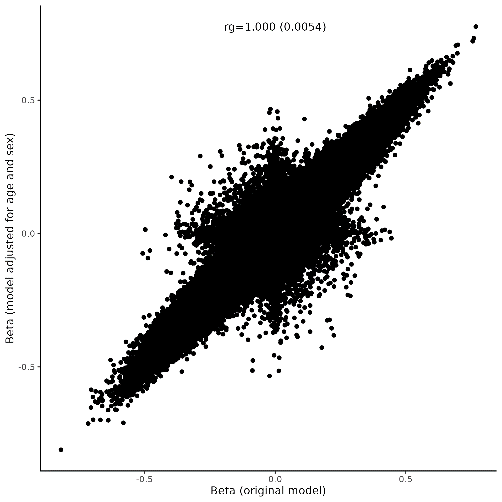


**B.** sTfR: Comparison of genetic estimates adjusted for age and sex vs those on the original model in INTERVAL (N=40,091). Miami plot (left) and scatter plot with genetic correlation (right)


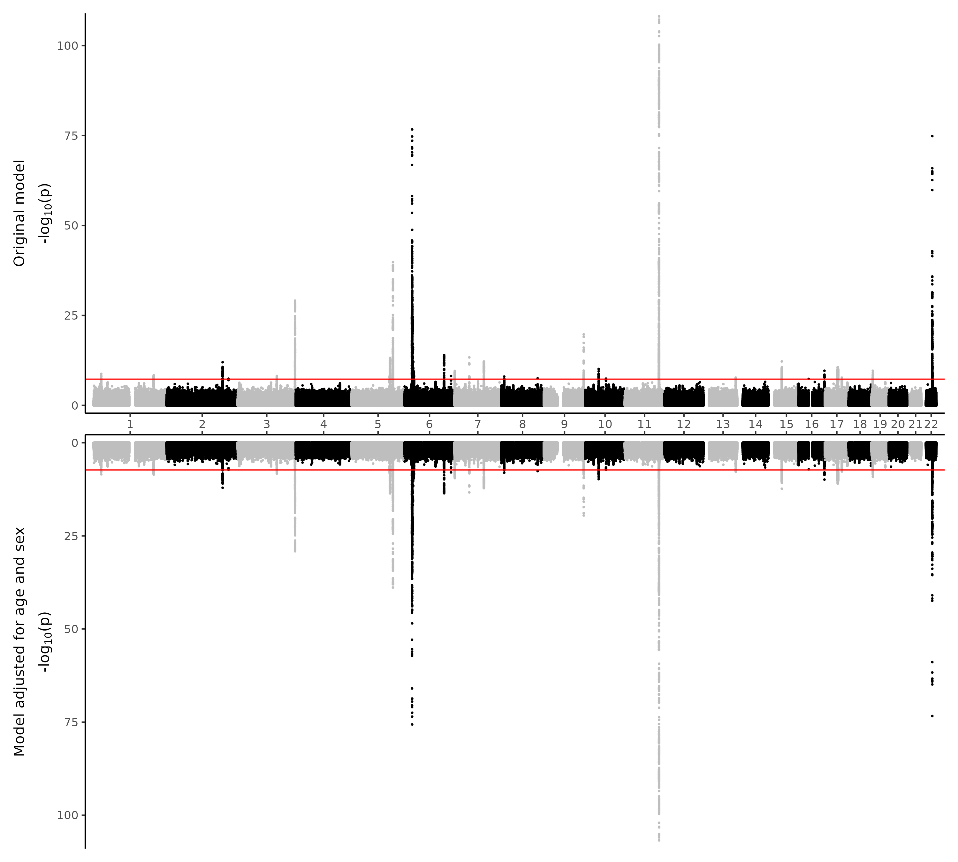


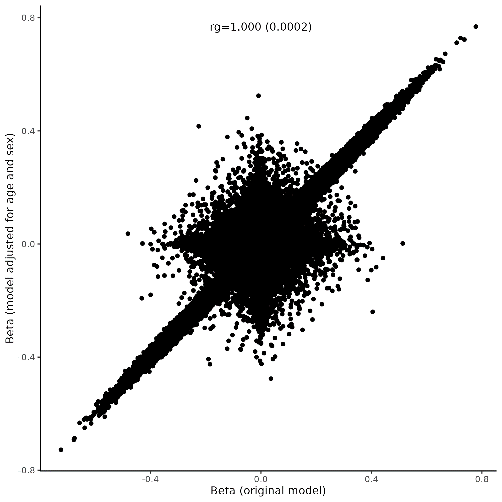


## **Supplementary Figure 12**. Sensitivity analyses adjusted for C-reactive protein

**A.** Miami plots and comparison of main sentinel variants (N=20) for genetic associations with hepcidin unadjusted and adjusted for C-reactive protein

| 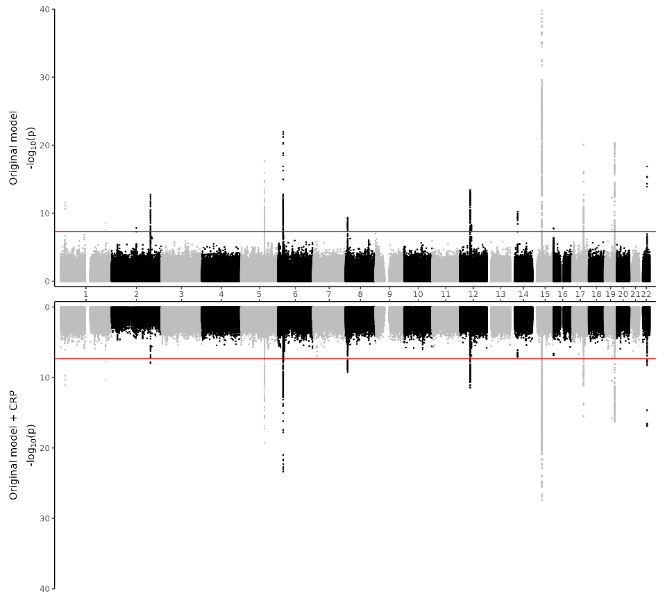  **B.** Miami plots and comparison of main sentinel variants (N=32) for genetic associations with sTfR unadjusted and adjusted for C-reactive protein | \| **CHR** \| **BP** \| **RSID** \| **A1** \| **A2** \| **Original model (unadjusted for C-reactive protein)** \| \| \| \| \| **Original model adjusted for C-reactive protein** \| \| \| \| \| **Beta absolute difference** \| \| --- \| --- \| --- \| --- \| --- \| --- \| --- \| --- \| --- \| --- \| --- \| --- \| --- \| --- \| --- \| --- \| \| **A1FREQ** \| **BETA** \| **SE** \| **P** \| **N** \| **A1FREQ** \| **BETA** \| **SE** \| **P** \| **N** \| \| 1 \| 22,584,002 \| rs75965181 \| A \| T \| 0.023 \| -0.118 \| 0.017 \| 2.76E-12 \| 91675 \| 0.024 \| -0.125 \| 0.018 \| 9.31E-12 \| 74855 \| 0.007 \| \| 1 \| 220,291,414 \| rs201255457 \| A \| ATC \| 0.989 \| -0.156 \| 0.026 \| 3.15E-09 \| 75972 \| 0.989 \| -0.173 \| 0.026 \| 5.48E-11 \| 74855 \| 0.017 \| \| 2 \| 121,310,269 \| rs6706968 \| A \| C \| 0.424 \| 0.030 \| 0.005 \| 1.43E-08 \| 82690 \| 0.422 \| 0.026 \| 0.006 \| 3.73E-06 \| 74855 \| 0.004 \| \| 2 \| 190,390,963 \| rs149682241 \| C \| G \| 0.026 \| 0.101 \| 0.015 \| 4.33E-11 \| 91675 \| 0.024 \| 0.105 \| 0.018 \| 2.04E-09 \| 74855 \| 0.003 \| \| 2 \| 190,521,054 \| rs7568449 \| T \| C \| 0.762 \| 0.043 \| 0.006 \| 1.88E-13 \| 89099 \| 0.762 \| 0.043 \| 0.006 \| 1.48E-11 \| 74855 \| 0.000 \| \| 5 \| 115,331,335 \| rs885122 \| A \| G \| 0.598 \| -0.043 \| 0.005 \| 2.28E-18 \| 91675 \| 0.598 \| -0.051 \| 0.006 \| 5.52E-20 \| 74855 \| 0.007 \| \| 6 \| 26,098,474 \| rs79220007 \| T \| C \| 0.930 \| 0.094 \| 0.010 \| 1.12E-22 \| 91675 \| 0.928 \| 0.105 \| 0.010 \| 4.59E-24 \| 74855 \| 0.012 \| \| 8 \| 10,577,987 \| rs10090558 \| A \| G \| 0.918 \| -0.060 \| 0.010 \| 4.64E-10 \| 78548 \| 0.918 \| -0.060 \| 0.010 \| 1.66E-09 \| 74855 \| 0.000 \| \| 12 \| 51,439,858 \| rs9739943 \| T \| C \| 0.059 \| 0.079 \| 0.010 \| 3.78E-14 \| 91675 \| 0.059 \| 0.081 \| 0.012 \| 3.64E-12 \| 74855 \| 0.002 \| \| 12 \| 57,735,045 \| rs11609805 \| A \| G \| 0.270 \| -0.032 \| 0.006 \| 5.24E-09 \| 91675 \| 0.276 \| -0.029 \| 0.006 \| 1.52E-06 \| 74855 \| 0.003 \| \| 14 \| 34,410,892 \| rs996347 \| T \| C \| 0.645 \| -0.033 \| 0.005 \| 6.06E-11 \| 91675 \| 0.647 \| -0.030 \| 0.006 \| 8.67E-08 \| 74855 \| 0.003 \| \| 15 \| 45,274,530 \| rs549876436 \| T \| C \| 0.907 \| 0.082 \| 0.011 \| 7.30E-15 \| 83099 \| 0.907 \| 0.078 \| 0.012 \| 1.44E-11 \| 72997 \| 0.004 \| \| 15 \| 45,319,959 \| rs2600895 \| A \| G \| 0.913 \| 0.082 \| 0.012 \| 2.73E-11 \| 88174 \| 0.910 \| 0.073 \| 0.014 \| 1.10E-07 \| 72997 \| 0.008 \| \| 15 \| 45,320,493 \| rs113000093 \| C \| G \| 0.089 \| -0.078 \| 0.013 \| 7.01E-10 \| 85598 \| 0.091 \| -0.068 \| 0.014 \| 1.11E-06 \| 72997 \| 0.010 \| \| 15 \| 45,387,550 \| rs199138 \| A \| G \| 0.076 \| -0.122 \| 0.009 \| 1.55E-40 \| 91675 \| 0.076 \| -0.110 \| 0.010 \| 1.21E-27 \| 74855 \| 0.012 \| \| 16 \| 353,122 \| rs71378510 \| A \| G \| 0.907 \| 0.050 \| 0.009 \| 1.62E-08 \| 91675 \| 0.905 \| 0.050 \| 0.010 \| 2.50E-07 \| 74855 \| 0.000 \| \| 17 \| 56,436,109 \| rs34523089 \| T \| C \| 0.162 \| 0.063 \| 0.007 \| 8.15E-21 \| 89817 \| 0.161 \| 0.057 \| 0.007 \| 1.93E-14 \| 72997 \| 0.006 \| \| 19 \| 35,775,902 \| rs104894696 \| A \| G \| 0.003 \| -0.387 \| 0.054 \| 4.90E-13 \| 76690 \| 0.003 \| -0.472 \| 0.057 \| 1.70E-16 \| 72997 \| 0.085 \| \| 19 \| 49,207,255 \| rs485073 \| A \| G \| 0.453 \| 0.046 \| 0.005 \| 4.64E-21 \| 91675 \| 0.434 \| 0.046 \| 0.006 \| 1.19E-16 \| 74855 \| 0.001 \| \| 22 \| 37,462,936 \| rs855791 \| A \| G \| 0.429 \| 0.042 \| 0.005 \| 1.34E-17 \| 91675 \| 0.424 \| 0.046 \| 0.005 \| 2.33E-17 \| 74855 \| 0.004 \| |
| --- | --- | --- | --- | --- | --- | --- | --- | --- | --- | --- | --- | --- | --- | --- | --- | --- | --- | --- | --- | --- | --- | --- | --- | --- | --- | --- | --- | --- | --- | --- | --- | --- | --- | --- | --- | --- | --- | --- | --- | --- | --- | --- | --- | --- | --- | --- | --- | --- | --- | --- | --- | --- | --- | --- | --- | --- | --- | --- | --- | --- | --- | --- | --- | --- | --- | --- | --- | --- | --- | --- | --- | --- | --- | --- | --- | --- | --- | --- | --- | --- | --- | --- | --- | --- | --- | --- | --- | --- | --- | --- | --- | --- | --- | --- | --- | --- | --- | --- | --- | --- | --- | --- | --- | --- | --- | --- | --- | --- | --- | --- | --- | --- | --- | --- | --- | --- | --- | --- | --- | --- | --- | --- | --- | --- | --- | --- | --- | --- | --- | --- | --- | --- | --- | --- | --- | --- | --- | --- | --- | --- | --- | --- | --- | --- | --- | --- | --- | --- | --- | --- | --- | --- | --- | --- | --- | --- | --- | --- | --- | --- | --- | --- | --- | --- | --- | --- | --- | --- | --- | --- | --- | --- | --- | --- | --- | --- | --- | --- | --- | --- | --- | --- | --- | --- | --- | --- | --- | --- | --- | --- | --- | --- | --- | --- | --- | --- | --- | --- | --- | --- | --- | --- | --- | --- | --- | --- | --- | --- | --- | --- | --- | --- | --- | --- | --- | --- | --- | --- | --- | --- | --- | --- | --- | --- | --- | --- | --- | --- | --- | --- | --- | --- | --- | --- | --- | --- | --- | --- | --- | --- | --- | --- | --- | --- | --- | --- | --- | --- | --- | --- | --- | --- | --- | --- | --- | --- | --- | --- | --- | --- | --- | --- | --- | --- | --- | --- | --- | --- | --- | --- | --- | --- | --- | --- | --- | --- | --- | --- | --- | --- | --- | --- | --- | --- | --- | --- | --- | --- | --- | --- | --- | --- | --- | --- | --- | --- | --- | --- | --- | --- | --- | --- | --- | --- | --- | --- | --- | --- | --- | --- | --- | --- | --- | --- | --- | --- | --- | --- | --- | --- | --- | --- | --- | --- | --- | --- | --- | --- | --- | --- | --- | --- | --- | --- | --- | --- | --- | --- | --- | --- | --- | --- | --- | --- | --- | --- | --- |
| 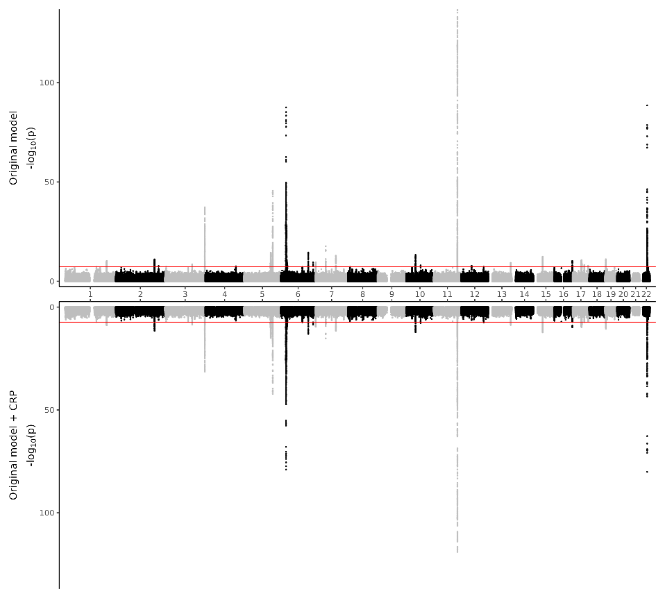 | \| **CHR** \| **BP** \| **RSID** \| **A1** \| **A2** \| **Original model (unadjusted for C-reactive protein)** \| \| \| \| \| **Original model adjusted for C-reactive protein** \| \| \| \| \| **Beta absolute difference** \| \| --- \| --- \| --- \| --- \| --- \| --- \| --- \| --- \| --- \| --- \| --- \| --- \| --- \| --- \| --- \| --- \| \| **A1FREQ** \| **BETA** \| **SE** \| **P** \| **N** \| **A1FREQ** \| **BETA** \| **SE** \| **P** \| **N** \| \| 1 \| 205,041,952 \| rs6696846 \| T \| C \| 0.484 \| -0.043 \| 0.007 \| 6.22E-11 \| 45,330 \| 0.486 \| -0.042 \| 0.007 \| 1.45E-09 \| 41,188 \| 0.002 \| \| 2 \| 191,357,694 \| rs35095338 \| A \| T \| 0.556 \| -0.046 \| 0.007 \| 9.67E-12 \| 44,341 \| 0.556 \| -0.047 \| 0.007 \| 1.55E-11 \| 41,188 \| 0.001 \| \| 2 \| 211,543,055 \| rs715 \| T \| C \| 0.688 \| -0.041 \| 0.007 \| 2.28E-08 \| 45,330 \| 0.687 \| -0.038 \| 0.008 \| 5.98E-07 \| 41,188 \| 0.003 \| \| 3 \| 133,539,500 \| rs11371594 \| G \| GA \| 0.845 \| 0.058 \| 0.010 \| 2.54E-09 \| 40,837 \| 0.844 \| 0.059 \| 0.010 \| 1.76E-09 \| 40,091 \| 0.001 \| \| 3 \| 195,795,618 \| rs112856048 \| A \| G \| 0.243 \| -0.100 \| 0.008 \| 5.93E-38 \| 45,330 \| 0.243 \| -0.096 \| 0.008 \| 5.38E-32 \| 41,188 \| 0.005 \| \| 3 \| 195,921,311 \| rs9325434 \| A \| G \| 0.141 \| -0.062 \| 0.010 \| 2.27E-10 \| 45,330 \| 0.142 \| -0.061 \| 0.010 \| 2.10E-09 \| 41,188 \| 0.001 \| \| 4 \| 151,199,080 \| rs2290846 \| A \| G \| 0.277 \| -0.041 \| 0.007 \| 3.11E-08 \| 45,330 \| 0.281 \| -0.039 \| 0.008 \| 2.75E-07 \| 41,188 \| 0.001 \| \| 5 \| 131,784,393 \| rs12521868 \| T \| G \| 0.427 \| 0.052 \| 0.007 \| 4.41E-15 \| 45,330 \| 0.432 \| 0.055 \| 0.007 \| 3.68E-15 \| 41,188 \| 0.002 \| \| 5 \| 141,482,333 \| rs116816795 \| T \| C \| 0.168 \| 0.127 \| 0.009 \| 3.13E-46 \| 45,330 \| 0.168 \| 0.128 \| 0.009 \| 6.92E-43 \| 41,188 \| 0.000 \| \| 5 \| 141,602,204 \| rs2906082 \| T \| C \| 0.783 \| 0.057 \| 0.008 \| 5.34E-12 \| 44,584 \| 0.782 \| 0.059 \| 0.009 \| 5.15E-12 \| 41,188 \| 0.002 \| \| 6 \| 25,957,426 \| rs72832593 \| T \| C \| 0.897 \| 0.130 \| 0.011 \| 8.09E-33 \| 45,330 \| 0.898 \| 0.131 \| 0.011 \| 9.18E-31 \| 41,188 \| 0.002 \| \| 6 \| 26,093,141 \| rs1800562 \| A \| G \| 0.075 \| -0.253 \| 0.013 \| 2.88E-88 \| 45,330 \| 0.077 \| -0.247 \| 0.013 \| 1.05E-79 \| 41,188 \| 0.006 \| \| 6 \| 135,427,159 \| rs9389269 \| T \| C \| 0.729 \| 0.059 \| 0.007 \| 3.10E-15 \| 45,330 \| 0.732 \| 0.058 \| 0.008 \| 9.43E-14 \| 41,188 \| 0.001 \| \| 6 \| 159,020,121 \| rs143437464 \| A \| G \| 0.009 \| -0.234 \| 0.037 \| 3.81E-10 \| 43,595 \| 0.009 \| -0.225 \| 0.038 \| 3.24E-09 \| 41,188 \| 0.009 \| \| 6 \| 159,026,327 \| rs200307986 \| A \| G \| 0.003 \| 0.358 \| 0.065 \| 2.69E-08 \| 42,671 \| 0.003 \| 0.374 \| 0.065 \| 8.45E-09 \| 41,188 \| 0.016 \| \| 7 \| 1,080,897 \| rs186044114 \| T \| C \| 0.852 \| -0.061 \| 0.010 \| 4.19E-10 \| 41,188 \| 0.852 \| -0.061 \| 0.010 \| 4.19E-10 \| 41,188 \| 0.000 \| \| 7 \| 50,427,982 \| rs6592965 \| A \| G \| 0.458 \| -0.058 \| 0.007 \| 2.43E-18 \| 45,330 \| 0.462 \| -0.056 \| 0.007 \| 6.71E-16 \| 41,188 \| 0.002 \| \| 7 \| 100,235,970 \| rs7385804 \| A \| C \| 0.627 \| -0.051 \| 0.007 \| 1.49E-13 \| 45,330 \| 0.629 \| -0.050 \| 0.007 \| 3.17E-12 \| 41,188 \| 0.001 \| \| 10 \| 45,953,767 \| rs7908745 \| A \| G \| 0.687 \| -0.054 \| 0.007 \| 5.61E-14 \| 45,330 \| 0.690 \| -0.054 \| 0.008 \| 6.79E-13 \| 41,188 \| 0.000 \| \| 10 \| 71,093,392 \| rs16926246 \| T \| C \| 0.133 \| 0.056 \| 0.010 \| 1.05E-08 \| 45,330 \| 0.135 \| 0.057 \| 0.010 \| 2.05E-08 \| 41,188 \| 0.001 \| \| 11 \| 117,021,097 \| rs187669805 \| C \| G \| 0.994 \| 0.407 \| 0.046 \| 1.94E-18 \| 43,487 \| 0.994 \| 0.391 \| 0.048 \| 2.98E-16 \| 40,091 \| 0.016 \| \| 11 \| 117,081,500 \| rs11216316 \| A \| C \| 0.892 \| -0.273 \| 0.011 \| 1.77E-137 \| 45,330 \| 0.894 \| -0.266 \| 0.012 \| 9.77E-118 \| 41,188 \| 0.007 \| \| 11 \| 117,088,082 \| rs2238005 \| T \| C \| 0.061 \| 0.118 \| 0.014 \| 2.71E-17 \| 45,330 \| 0.058 \| 0.117 \| 0.015 \| 1.97E-15 \| 41,188 \| 0.001 \| \| 12 \| 51,783,420 \| rs10876169 \| A \| T \| 0.410 \| 0.039 \| 0.007 \| 1.85E-08 \| 44,584 \| 0.409 \| 0.034 \| 0.007 \| 1.23E-06 \| 41,188 \| 0.004 \| \| 13 \| 110,401,304 \| rs76944188 \| T \| C \| 0.942 \| -0.090 \| 0.015 \| 5.45E-10 \| 45,330 \| 0.943 \| -0.089 \| 0.015 \| 5.86E-09 \| 41,188 \| 0.001 \| \| 15 \| 45,395,901 \| rs75922593 \| G \| GT \| 0.066 \| 0.112 \| 0.014 \| 3.64E-15 \| 40,837 \| 0.066 \| 0.113 \| 0.014 \| 3.36E-15 \| 40,091 \| 0.001 \| \| 16 \| 88,567,333 \| rs74035509 \| T \| C \| 0.083 \| 0.083 \| 0.013 \| 6.36E-11 \| 44,584 \| 0.083 \| 0.084 \| 0.013 \| 1.91E-10 \| 41,188 \| 0.001 \| \| 17 \| 43,556,807 \| rs55925547 \| T \| C \| 0.801 \| -0.057 \| 0.009 \| 2.46E-11 \| 43,244 \| 0.800 \| -0.056 \| 0.009 \| 1.84E-10 \| 40,091 \| 0.001 \| \| 17 \| 57,925,649 \| rs1292072 \| A \| G \| 0.794 \| -0.048 \| 0.008 \| 7.13E-09 \| 45,330 \| 0.795 \| -0.047 \| 0.009 \| 5.86E-08 \| 41,188 \| 0.001 \| \| 17 \| 76,401,328 \| rs1976703 \| T \| C \| 0.493 \| 0.039 \| 0.007 \| 1.57E-08 \| 41,826 \| 0.489 \| 0.036 \| 0.007 \| 2.68E-07 \| 40,091 \| 0.003 \| \| 19 \| 4,502,282 \| rs13041 \| T \| C \| 0.518 \| 0.048 \| 0.007 \| 8.53E-12 \| 44,584 \| 0.521 \| 0.047 \| 0.007 \| 1.62E-10 \| 41,188 \| 0.001 \| \| 22 \| 37,462,936 \| rs855791 \| A \| G \| 0.434 \| 0.134 \| 0.007 \| 2.70E-89 \| 45,330 \| 0.438 \| 0.133 \| 0.007 \| 7.57E-81 \| 41,188 \| 0.001 \| |

The meta-analyses of hepcidin GWASs include up to 91,675 participants (original model) and up to 74,855 participants (original model adjusted for C-reactive protein). The meta-analyses of sTfR GWASs include up to 45,330 participants (original model) and up to 41,188 participants (original model adjusted for C-reactive protein).

# References

1. Astle WJ, Elding H, Jiang T, et al. The Allelic Landscape of Human Blood Cell Trait Variation and Links to Common Complex Disease. *Cell* 2016; **167**(5): 1415-29 e19.

2. Pattaro C, Gogele M, Mascalzoni D, et al. The Cooperative Health Research in South Tyrol (CHRIS) study: rationale, objectives, and preliminary results. *J Transl Med* 2015; **13**: 348.

3. Vitart V, Rudan I, Hayward C, et al. SLC2A9 is a newly identified urate transporter influencing serum urate concentration, urate excretion and gout. *Nat Genet* 2008; **40**(4): 437-42.

4. Loh PR, Tucker G, Bulik-Sullivan BK, et al. Efficient Bayesian mixed-model analysis increases association power in large cohorts. *Nat Genet* 2015; **47**(3): 284-90.

5. Hansen TF, Banasik K, Erikstrup C, et al. DBDS Genomic Cohort, a prospective and comprehensive resource for integrative and temporal analysis of genetic, environmental and lifestyle factors affecting health of blood donors. *BMJ Open* 2019; **9**(6): e028401.

6. Gudbjartsson DF, Sulem P, Helgason H, et al. Sequence variants from whole genome sequencing a large group of Icelanders. *Sci Data* 2015; **2**: 150011.

7. Dowsett J, Didriksen M, Larsen MH, et al. No association between plasma hepcidin levels and restless legs syndrome - results from the Danish Blood Donor Study. *Sleep Med* 2021; **88**: 68-73.

8. Lobier M, Niittymaki P, Nikiforow N, et al. FinDonor 10 000 study: a cohort to identify iron depletion and factors affecting it in Finnish blood donors. *Vox Sang* 2020; **115**(1): 36-46.

9. Howie BN, Donnelly P, Marchini J. A flexible and accurate genotype imputation method for the next generation of genome-wide association studies. *PLoS Genet* 2009; **5**(6): e1000529.

10. Kurki MI, Karjalainen J, Palta P, et al. FinnGen: Unique genetic insights from combining isolated population and national health register data. 2022: 2022.03.03.22271360.

11. Sun BB, Kurki MI, Foley CN, et al. Genetic associations of protein-coding variants in human disease. *Nature* 2022; **603**(7899): 95-102.

12. Zhou W, Nielsen JB, Fritsche LG, et al. Efficiently controlling for case-control imbalance and sample relatedness in large-scale genetic association studies. *Nat Genet* 2018; **50**(9): 1335-41.

13. Ferrucci L, Bandinelli S, Benvenuti E, et al. Subsystems contributing to the decline in ability to walk: bridging the gap between epidemiology and geriatric practice in the InCHIANTI study. *J Am Geriatr Soc* 2000; **48**(12): 1618-25.

14. Melzer D, Perry JR, Hernandez D, et al. A genome-wide association study identifies protein quantitative trait loci (pQTLs). *PLoS Genet* 2008; **4**(5): e1000072.

15. Li Y, Willer CJ, Ding J, Scheet P, Abecasis GR. MaCH: using sequence and genotype data to estimate haplotypes and unobserved genotypes. *Genet Epidemiol* 2010; **34**(8): 816-34.

16. Colonna V, Pistis G, Bomba L, et al. Small effective population size and genetic homogeneity in the Val Borbera isolate. *Eur J Hum Genet* 2013; **21**(1): 89-94.

17. Traglia M, Sala C, Masciullo C, et al. Heritability and demographic analyses in the large isolated population of Val Borbera suggest advantages in mapping complex traits genes. *PLoS One* 2009; **4**(10): e7554.

18. Zhou X, Stephens M. Genome-wide efficient mixed-model analysis for association studies. *Nat Genet* 2012; **44**(7): 821-4.

19. Moore C, Sambrook J, Walker M, et al. The INTERVAL trial to determine whether intervals between blood donations can be safely and acceptably decreased to optimise blood supply: study protocol for a randomised controlled trial. *Trials* 2014; **15**: 363.

20. Di Angelantonio E, Thompson SG, Kaptoge S, et al. Efficiency and safety of varying the frequency of whole blood donation (INTERVAL): a randomised trial of 45 000 donors. *Lancet* 2017; **390**(10110): 2360-71.

21. Holle R, Happich M, Lowel H, Wichmann HE, Group MKS. KORA--a research platform for population based health research. *Gesundheitswesen* 2005; **67 Suppl 1**: S19-25.

22. Wichmann HE, Gieger C, Illig T, Group MKS. KORA-gen--resource for population genetics, controls and a broad spectrum of disease phenotypes. *Gesundheitswesen* 2005; **67 Suppl 1**: S26-30.

23. Galesloot TE, Vermeulen SH, Swinkels DW, et al. Cohort Profile: The Nijmegen Biomedical Study (NBS). *Int J Epidemiol* 2017; **46**(4): 1099-100j.

24. Galesloot TE, Vermeulen SH, Geurts-Moespot AJ, et al. Serum hepcidin: reference ranges and biochemical correlates in the general population. *Blood* 2011; **117**(25): e218-25.

25. Kroot JJ, Laarakkers CM, Geurts-Moespot AJ, et al. Immunochemical and mass-spectrometry-based serum hepcidin assays for iron metabolism disorders. *Clin Chem* 2010; **56**(10): 1570-9.

26. Hillege HL, Fidler V, Diercks GF, et al. Urinary albumin excretion predicts cardiovascular and noncardiovascular mortality in general population. *Circulation* 2002; **106**(14): 1777-82.

27. Marchini J, Howie B. Genotype imputation for genome-wide association studies. *Nat Rev Genet* 2010; **11**(7): 499-511.

28. Bulik-Sullivan BK, Loh PR, Finucane HK, et al. LD Score regression distinguishes confounding from polygenicity in genome-wide association studies. *Nat Genet* 2015; **47**(3): 291-5.

29. Tenesa A, Haley CS. The heritability of human disease: estimation, uses and abuses. *Nat Rev Genet* 2013; **14**(2): 139-49.

30. Yang J, Weedon MN, Purcell S, et al. Genomic inflation factors under polygenic inheritance. *Eur J Hum Genet* 2011; **19**(7): 807-12.

31. Kamat MA, Blackshaw JA, Young R, et al. PhenoScanner V2: an expanded tool for searching human genotype-phenotype associations. *Bioinformatics* 2019; **35**(22): 4851-3.

32. Stranger BE, Montgomery SB, Dimas AS, et al. Patterns of cis regulatory variation in diverse human populations. *PLoS Genet* 2012; **8**(4): e1002639.

33. Consortium GT. The GTEx Consortium atlas of genetic regulatory effects across human tissues. *Science* 2020; **369**(6509): 1318-30.

34. Sun BB, Maranville JC, Peters JE, et al. Genomic atlas of the human plasma proteome. *Nature* 2018; **558**(7708): 73-9.

35. Genomes Project C, Auton A, Brooks LD, et al. A global reference for human genetic variation. *Nature* 2015; **526**(7571): 68-74.

36. Wallace C. A more accurate method for colocalisation analysis allowing for multiple causal variants. *PLoS Genet* 2021; **17**(9): e1009440.

37. Wang G, Sarkar A, Carbonetto P, Stephens M. A simple new approach to variable selection in regression, with application to genetic fine mapping. 2020; **82**(5): 1273-300.

38. Codd V, Wang Q, Allara E, et al. Polygenic basis and biomedical consequences of telomere length variation. *Nat Genet* 2021; **53**(10): 1425-33.

39. Dorajoo R, Chang X, Gurung RL, et al. Loci for human leukocyte telomere length in the Singaporean Chinese population and trans-ethnic genetic studies. *Nat Commun* 2019; **10**(1): 2491.

40. Holmes MV, Davey Smith G. Problems in interpreting and using GWAS of conditional phenotypes illustrated by 'alcohol GWAS'. *Mol Psychiatry* 2019; **24**(2): 167-8.

41. Hartwig FP, Tilling K, Davey Smith G, Lawlor DA, Borges MC. Bias in two-sample Mendelian randomization when using heritable covariable-adjusted summary associations. *Int J Epidemiol* 2021; **50**(5): 1639-50.

42. Bell S, Rigas AS, Magnusson MK, et al. A genome-wide meta-analysis yields 46 new loci associating with biomarkers of iron homeostasis. *Commun Biol* 2021; **4**(1): 156.

43. Brion MJ, Shakhbazov K, Visscher PM. Calculating statistical power in Mendelian randomization studies. *Int J Epidemiol* 2013; **42**(5): 1497-501.

44. Camaschella C. Iron-deficiency anemia. *N Engl J Med* 2015; **372**(19): 1832-43.
