# Supplementary material for: Novel loci and biomedical consequences of iron homoeostasis variation
Source: Commun Biol. 2024 Dec 6;7:1631. doi: 10.1038/s42003-024-07115-3 (PMC11624196; doi:10.1038/s42003-024-07115-3)
Supplement: Supplementary file 2 — Description of Additional Supplementary Files [file 42003_2024_7115_MOESM2_ESM.pdf]

Description of Additional Supplementary Files

File name: Supplementary Data 1.
Description: Characteristics of genetic cohorts with hepcidin and sTfR measurements

File name: Supplementary Data 2.

Description: Genetic and phenotipic correlations between hepcidin, sTfR, and conventional iron biomarkers (ferritin, iron, TIBC, TSAT)

File name: Supplementary Data 3.

Description: Full annotation of 55 conditionally independent variants associated with hepcidin (N=23) and sTfR (N=32)

File name: Supplementary Data 4.

Description: Study-specific associations of genetic variants associated to hepcidin and sTfR

File name: Supplementary Data 5.

Description: Variant effect prediction

File name: Supplementary Data 6.

Description: Phenome scans for the 62 conditionally-independent variants associated with hepcidin or sTfR

File name: Supplementary Data 7.

Description: Results of colocalization analysis for hepcidin and sTfR - signals with strong evidence of colocalization

File name: Supplementary Data 8.

Description: Candidate-gene mapping process

File name: Supplementary Data 9.

Description: Genetic pleiotropy

File name: Supplementary Data 10.

Description: Loci analysed in locus-based Mendelian randomization and colocalization analyses

File name: Supplementary Data 11.

Description: Genetic variants used in MR

File name: Supplementary Data 12.

Description: Locus-based MR results

File name: Supplementary Data 13.

Description: Post-MR colocalization

File name: Supplementary Data 14.

Description: Iron status MR results

File name: Supplementary Data 15.

Description: Sensitivity analysis for key iron-status MR results using more liberal sets of polygenic instruments
